# Allosteric binding sites in Rab11 for potential drug candidates

**DOI:** 10.1371/journal.pone.0198632

**Published:** 2018-06-06

**Authors:** Ammu Prasanna Kumar, Suryani Lukman

**Affiliations:** Department of Chemistry, College of Arts and Sciences, Khalifa University of Science and Technology, Abu Dhabi, United Arab Emirates; Consejo Superior de Investigaciones Cientificas, SPAIN

## Abstract

Rab11 is an important protein subfamily in the RabGTPase family. These proteins physiologically function as key regulators of intracellular membrane trafficking processes. Pathologically, Rab11 proteins are implicated in many diseases including cancers, neurodegenerative diseases and type 2 diabetes. Although they are medically important, no previous study has found Rab11 allosteric binding sites where potential drug candidates can bind to. In this study, by employing multiple clustering approaches integrating principal component analysis, independent component analysis and locally linear embedding, we performed structural analyses of Rab11 and identified eight representative structures. Using these representatives to perform binding site mapping and virtual screening, we identified two novel binding sites in Rab11 and small molecules that can *preferentially* bind to different conformations of these sites with high affinities. After identifying the binding sites and the residue interaction networks in the representatives, we computationally showed that these binding sites may *allosterically* regulate Rab11, as these sites communicate with switch 2 region that binds to GTP/GDP. These two allosteric binding sites in Rab11 are also similar to two allosteric pockets in Ras that we discovered previously.

## Introduction

The largest member of the Ras superfamily is the Rab family of small GTPases, which contains almost 70 proteins. Rab proteins are important regulators of intracellular membrane trafficking, from the assembly of transport vesicles to their fusion with membranes. Rab proteins cycle between an inactive GDP-bound conformation and an active GTP-bound conformation. GTP-bound Rab proteins can recruit distinct set of downstream effectors to membranes; these effectors are essential for the formation, trafficking, tethering and fusion of transport vesicles.

Rabs have been grouped into different subfamilies based on their distinct sequence motifs. The three members of Rab11 subfamily (Rab11a, Rab11b and Rab11c/Rab25) are closely related, evolutionary conserved, and differentially expressed. Rab11a is ubiquitously expressed, Rab11b is enriched in brain, heart, and testes [1], and Rab25 is only expressed in epithelial cells[2]. Rab11a and Rab11b proteins share 89% sequence identity, whereas Rab11a or Rab11b share 61% and 66% identity with Rab25, respectively [3]. In this study, we have excluded Rab25 as there were only few Rab25 crystallographic structures available in the Research Collaboratory for Structural Bioinformatics (RCSB) Protein Data Bank (PDB) as of 7^th^ July 2017, and these PDB entries have no references. Therefore, we have focused on Rab11a and Rab11b isoforms of Rab11. We detailed their subcellular localizations and molecular function in the S1 File.

Rab11 interacts with diverse families of interacting proteins (FIPs) and these interactions regulate different transport pathways such as recycling of transferrin, cytokinesis, epidermal growth factor receptor, etc [4][5][6][7][8]. Rab11 interacts with Myosin 5a and Myosin 5b motor proteins to recruit these motor proteins to their cellular cargo [9]. Rab11 also interacts with ecotropic viral integration site 5 (EVi5) protein to regulate vesicle trafficking, cytokinesis and cell cycle [10][11]. Rab11 interacts with TBC1D14 protein [12]and regulates the formation of autophagosomes. Rab11 interacts with Sec15 protein [13][14][11]and this interaction is thought to facilitate Rab11 function during cytokinesis. There are many other proteins with which Rab11 interacts such as type II cGMP dependent protein kinase (PKGII) [15], phoshatidylinositol 4-kinase III beta (P14KB)[16], β_2_-adrenergic receptor [17], brain-derived neurotrophic factor-dependent TrkB (TrkB-FL) receptors[18],b-isoform of the thromboxane A_2_ receptor (TPb) [19], GRAB[20], TRPV5 and TRPV6 Ca^2+^ channels [21][22], etc.

Rab11 has been related to a number of diseases. Rab11 has roles in hypoxia-stimulated cell invasion in breast carcinoma [23]. Yoon *et al*. have shown that hypoxia stimulates carcinoma invasion by modulating Rab11 [23]. Chung *et al*. have shown that Rab11 together with E-cadherin protein might be potential markers for colorectal cancer progression and treatment [24]. Rab11 has implications in influenza A virus morphogenesis and budding. In infected cells, reduction of Rab11 leads to a significant reduction in the release of influenza viral particles [25]. Rab11 is also associated with the inclusions of various *Chlamydia* bacteria and they are important regulators of *Chlamydia* infections [26].*Chlamydia pneumoniae* is an intracellular pathogen that belongs to the *Chlamydial* species, which is a common cause of upper respiratory infections and pneumonia and has been associated with chronic inflammatory conditions such as atherosclerosis, chronic obstructive pulmonary disease, and asthma. Cortes *et al*. have shown that Rab11 interact with Cpn0585, a *Chlamydia pneumonia* inclusion membrane protein [27].Later, Lipinski *et al*. have shown that reduction of Rab11 inhibited *Chlamydia-*induced fragmentation of the Golgi apparatus, together with reductions in bacterial progeny and lipid transport [28]. Rab11 proteins are also implicated in many other diseases such as Huntington’s disease [29], Alzheimer’s disease [30], Parkinson's disease [31], type 2 diabetes[32] andskin cancer [33][11].

The involvement of Rab11 in multiple diseases renders it a medically important target. It is highly desirable to design small molecules drugs that can regulate Rab11. However, no effective modulator that can regulate Rab11a or Rab11b is known currently. In fact, only few selectivemodulators/inhibitors have been designed to regulate the Rab proteins. The first inhibitor designed to regulate the Rab proteins was'CID1067700', which targets the active site of Rab7[34]. Later design of a Rab25 modulator was a stapled peptide 'RFP14' that selectively blocks the interaction between Rab25 and its effectors, by mimicking a common interaction motif present in the effectors[35].To design an effective therapeutic strategy for regulating a protein, it is necessary to identify druggable sites/regions/pockets on the protein surface, where small molecules can bind to and modulate the aberrant protein function [36][37]. Some binding pockets on the protein surface may be formed transiently and are often not revealed in a single static structure of the protein resolved using methods such as X-ray crystallography [38][37]. However, accounting for such transient pockets in structure-based drug design has shown to be critical by a number of studies [39][36][40]. For example, Raltegravir (Isentress), the first FDA-approved drug targeting the HIV-1 integrase protein, was discovered using a transient pocket on the protein surface [41][42][37]. Inspired by the success of this study, we have previously identified novel binding sites in Ras[39]and Rab1 proteins[37], their molecular details and associations with diseases are available from S1 File.

In this study, we have analyzed multiple Rab11 structures from the PDB using a unique combination of clustering approaches and successfully identified eight representative structures. Employing these representatives, we identified two novel binding sites in Rab11 and identified several lead compounds that can bind to these binding sites. Through computational analyses, we have shown that the two novel binding sites can be allosteric. We have also compared Rab11 with Rab1 and Ras proteins, which we have studied previously [37][39], to examine the differences and similarities in their dynamics and structures.

## Results

We first retrieved Rab11 structures from the PDB and identified Rab11 representative structures, through an ensemble of methods comprising principal component analysis (PCA), independent component analysis (ICA) and locally linear embedding (LLE). Next, we examined the flexibility of Rab11 residues, identified potentially allosteric and/or transient binding sites, and screened potential binders to these sites. We compared the interactions shown by Rab1 (which we have examined in a previous study [37]) and Rab11 with interacting partners. We have also compared binding sites in Rab11 with that of Rab1 and Ras proteins.

### Rab11 representative structures

We used Basic Local Alignment Search Tool (BLAST) to form an ensemble of 27 Rab11 structures (S1 Table; See Methods and S1 File). We observed clustering of Rab11a and Rab11b isoforms in the ensemble. The sequence similarity (89%) of these Rab11 isoforms explains why they are clustered together. In a comparative analysis of human Rab proteins, Stein *et al*. have shown that these Rab11 isoforms cluster together based on not only the similarity of their sequence but also their molecular interaction fields [43]. They suggested that Rab isoforms that are very close in primary sequence, exhibited similar molecular interaction field around their surfaces, and are thus expected to bind to the same effector proteins and perform similar functions.

Interpretation of high dimensional data can be difficult, and may often require the employment of multiple analyses. For the analyses of Rab11 dataset, we employed Dynamical Cross Correlation Matrix (DCCM) analysis, PCA [44], ICA [45]and LLE [46]. DCCM analysis helped us to identify correlated movements between different regions of Rab11 proteins. PCA, ICA and LLE were used for mapping the Rab11 ensemble to low dimensional spaces, and they act complementarily in dimensionality reduction (see S1 File). We performed hierarchical clustering in the low dimensional spaces to identify representative structures from Rab11 structures.

In the DCCM analysis(S1 File), we observed correlated motions withinswitch 1 (E39-V46), switch 2 (A68-A79) and interswitch (E47-T67) regions. We also observed some correlated movements between different regions of Rab11 that can provide important clues about long range communications in Rab11. It is notable that the interswitch region (E47-T67), switch 2 (A68-A79) and the adjacent region (residues Y80-K107) have strong correlated movements with the P-loop (residues G18-S25) that also contribute to nucleotide binding. Residues E100-V122 has correlated motions with residues Y80-W105. Residues R140-N160 has strong anti-correlated movements with the interswitch region and strong correlated movements with residues E100-V122. We have seen in the later stages of our study that these correlations are important and could play an important role in allosteric communication for Rab11 (see subsequent sections).

Next, we performed dimensionality reduction on the ensemble of 27 structures. First, we performed PCA [44] on the ensemble. Almost 70% of the variance is captured in the first three principal components (PCs) (S1 Fig). We projected the structures in the Rab11 ensemble onto the first two PCs, and to the first and third PCs (S1 Fig). We identified clusters in the PC spaces based on dendrograms (S2 Fig). In the ensemble, there are many Rab11 structures that are co-crystallized with interacting partners such as Myosin 5a, Myosin 5b, family-interacting protein 2 (FIP2), family-interacting protein 3 (FIP3), Rabin8, P14KB and PKGII. We observed separate clustering of most of the Rab11structures co-crystallized with distinct interacting partners in PCA.

We identified 7 clusters along PC1 and PC2 (Panel A in S2 Fig). Rab11a structures co-crystallized with different other proteins such as Myosin 5a (PDB entries 5JCZ_A and 5JCZ_D), FIP2 (PDB entries 2GZH, 2GZD and 4C4P) and Rabin8 (PDB entry 4UJ5_B) clustered separately in different groups. Rab11b structure co-crystallized PKGII (PDB entry 4OJK_A) clustered separately. Among the Rab11 structures that do not have any interacting partners, GDP-bound Rab11a structures (PDB entries 1OIV_A and 1OIV_B) and GTP analog-bound Rab11 structures (see S1 Table) formed two separate clusters.

We identified 6 clusters along PC1 and PC3 (Panel B in S2 Fig). In these two PCs, Rab11a structures co-crystallized with Myosin 5b (PDB entries 4LX0_A and 4LX0_C) and P14KB (PDB entries 5C46_F, 4D0L_B, 4D0L_D and 4D0L_F) formed two separate clusters. GDP-bound and GTP-analog bound Rab11 structures clustered separately as in the first two PCs.

We next employed ICA [45]for the analysis of Rab11 structures. We identified 5 clusters in the subspace formed by the first two independent components (S3 Fig). ICA co-clustered Rab11a structures co-crystallized with P14KB and Myosin 5b together in a separate group. The FIP2-bound and FIP3-bound Rab11a structures clustered together separate from others. Rab11a co-crystallized with Rabin8 clustered separately. GTP analog-bound Rab11 structures without interacting partners clustered together in a separate group.

We then employed LLE [46]analysis on the ensemble of 27 structures. After mapping the Rab11 structural data to two-dimensional space using LLE, we observed 7 clusters (S4 Fig). Rab11 structures co-crystallized with interacting partners such as Myosin 5a, Myosin 5b, P14KB and Rabin8 clustered separately in separate groups. The FIP2-bound and FIP3-bound Rab11 structures clustered together in a separate group. All the GTP analog-bound Rab11 structures without interacting partners clustered together in a separate group.

**Fig 1:**
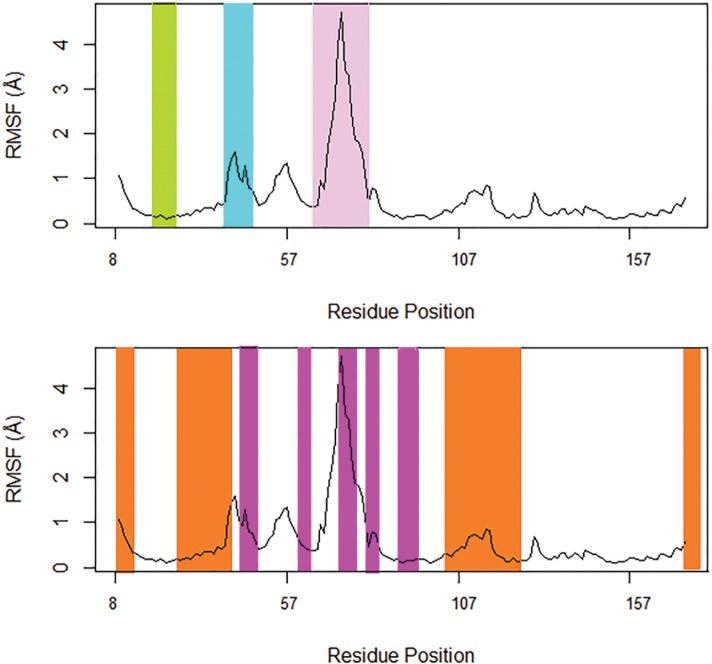
Structural dynamics of Rab11 protein as quantified by residual flexibility. Switches 1 and 2 are colored in cyan and pink, respectively. P-loop is highlighted in green. RabF and RabSF regions are colored in magenta and orange, respectively.

To quantify the residual flexibility of Rab proteins employed in our study, we performed Root Mean Square Fluctuation (RMSF) analysis of Rab structures in the ensemble. Regionally, Rab conserved residues can be clustered into two types of motifs: RabF and RabSF[47]. While RabF motifs differentiate Rab proteins from the other members of Ras superfamily, RabSF motifs differentiate a member of Rab family from the other members of Rab family (S5 Fig). RabF motifs overlap with switch 1 and switch 2 (Fig 1); the switches adopt distinct conformations when GDP/GTP binds to Rab. We observed that in the Rab11 structures employed in our study, switch 1 region (residues E39-V46) and switch 2 region (residues A68-A79), which encompasses the RabF3 region (residues R72-T77), are the most flexible regions. Among the switch regions, switch 2 region has the highest flexibility.

We also performed Root Mean Square Deviation (RMSD) analysis on the Rab11 structures based on their all residues, and the switch 1, interswitch and switch 2 residues, for which high flexibility is observed during the RMSF analysis. During RMSD analysis using all residues, we could observe that structures co-crystallized PKGII, Myosin 5a, Myosin 5b, Rabin8 and P14KB were separated from others; i.e. they formed separate clusters (S6 Fig), even though the inter-cluster RMSDs were small. On RMSD analysis of switch 1, GDP-bound Rab11a structures (PDB entries 1OIV_A and 1OIV_B) and Rab11 structures co-crystallized with different other proteins such as Myosin 5b and PKGII clustered separately in different clusters (S7 Fig). However, the switch 1 region of Rab11 structures in overall show high similarity (all RMSD values close to or less than 2 Å). Interswitch region separated Rab11 structures with different interacting partners such as Myosin 5b and PKGII in different clusters based on their RMSD values (S8 Fig). The interswitch regions of Rab11 structures are also found to be highly similar (RMSD values less than 1 Å). Switch 2 separated the GDP-bound Rab11a structures and Rab11 structures with different interacting partners such as Myosin 5a, Myosin 5b, P14KB and PKGII in different clusters based on their RMSDs (S9 Fig). Switch 2 regions of Rab11 structures show more deviations as compared to the switch 1 and the interswitch regions, with RMSD values lying in the range of 0–5 Å.

### Comparison of PCA, ICA and LLE

We have performed PCA, ICA and LLE on Rab11 structural ensemble with the objective of identifying representative structures. In these different analyses, we observed that Rab11 structures that are co-crystallized with different interacting partners have different structural dynamics. We also observed separate clustering of Rab11 structures that are crystallized without interacting partners. Among such structures, GTP analog-bound and GDP-bound Rab11 structures shows different structural dynamics. Although we obtained similar results in general using the different approaches, we could also observe some differences among them.

In PCA, the first two PCs could only capture 30% of the variance, so we needed to employ the first three PCs (that capture almost 70% of the variance) to get a better understanding of the Rab11 structural data. With the first three PCs, we observed separate clustering of the Rab11 structures co-crystallized with different interacting partners. PCA also clustered GDP-bound Rab11 structures and GTP analog-bound Rab11 structures, which are crystallized without interacting partners, in separate groups. Although ICA gave many results similar to PCA, it could not separate all Rab11 structures co-crystallized with different interacting partners and the GDP-bound Rab11a structures. As in ICA, GDP-bound Rab11 structures also didn't cluster separately in LLE. Interestingly, in LLE, by mapping the Rab11 structures to a two-dimensional space, we could see separate clustering of most of the structures with different interacting partners except PDB entry 4OJK_A. Thus, LLE could represent the relationship among Rab11 structures using few dimensions better than the other methods.

The comparison of results from PCA, ICA and LLE proves that multiple analyses on high dimensional data are beneficial for better interpretation of such data. Since LLE could separate most of the structures with interacting partners, we have chosen the clustroids of clusters observed during LLE analysis as the representative structures. Since LLE could not separate PDB entry 4OJK_A (Rab11b), which was separated by PCA from other structures, we have included it in our list of clustroids for further study.

### Comparison of interactions exhibited by Rab1 and Rab11 with interacting partners

We have compared the mode of interaction of Rab11 with that observed in Rab1, for which novel binding sites were previously identified [37]. There are many Rab1 (Rab1a and Rab1b) structures available in PDB that are co-crystallized with interacting partners such as guanine nucleotide exchange factor (GEF) SidM/DrrA[48], LidA[49], GTPase activating proteins (GAPs) such as LepB[50][51][52],VirA[53], and ESPG [53], that are from human pathogens, and TBC1D20, which is a Rab1- and Rab2 specific GAP that is from humans [54]. The mode of interaction exhibited by Rab1 with these partners is similar to that of Rab11 in that mostly residues in and near the switch 1, the switch 2 and the interswitch regions are involved in interactions, and subsequently structural changes are induced in these regions. In Rab1, in addition to these regions, residues from P-loop (residues G18-S25 in Rab1a) also interact with partners. While the residues in switch 2 and P-loop regions are mostly conserved in Rab1 and Rab11, the residues in and near the switch 1 region (residues F31-Y40 in Rab1a) are poorly conserved (S2 and S3 Tables). These residues are part of the RabSF2 (residues 26–42) region. The interactions of P14KB and Rabin8 with Rab11a are also different from that observed between other effectors and Rab1/Rab11. P14KB and Rabin8 make only few contacts with Rab11 (residues L38, E39, H130, L131 that are not conserved in Rab1) and the switch 2 is not involved in the interactions. From all these, we infer that these variable residues may confer specificity for Rab1 and Rab11 for interacting with their respective partners.

In visual examination of the superposed structures of Rab1, we observed that the same Rab isoforms (i.e. Rab1a or Rab1b) that interact with same effector proteins have similar conformational changes induced in their switch 1 region and they are different from that observed in Rab11 (S10 Fig). For example, in Rab1a structures co-crystallized with LidA (PDB entries 3SFV_A and 3TKL_A), it was interesting to find that the structural changes induced are similar (S11 Fig), even when the same effector interacted with Rab1 in different nucleotide-bound states [49] (GDP-bound and GTP-bound states). Another difference is that, the Rab1 switch 2 region adopts a well ordered conformation following effector binding, while the switch 2 in Rab11 is disordered and shows high flexibility [49].

In all Rab11a structures that interact with effectors, except structures in complex with P14KB, we observed that residue S40 that is pointing away from GTP-bound Rab11 (without any interacting partners) [55]adopts a different conformation (see Discussion). In Rab1, however this Serine residue is not conserved, instead they possess a Tyrosine at this position. When we compared PDB entries 3TKL_A (Rab1a-GTP structure co-crystallized with LidA) [49] and 3SFV_A (Rab1a-GDP that adopts a GTP like conformation after interacting with LidA) [49], we observed that this Tyrosine residue also interacts with the nucleotide through hydrogen bond and adopts a different conformation from that observed in Rab11 (S11 Fig).

### Rab11 binding sites identified by FTMap

We used FTMap[56]to identify residues that form binding sites, through both hydrophobic and electrostatic (hydrogen bond) interactions, in Rab11 representative structures (S4 Table and S12 Fig). Residues forming the active site of Rab11 (i.e. G1-G5 loops, S2 File) and the residues in and near the switch 1 and switch 2, that are involved in interactions with other proteins (S2 Table), are identified by FTMap in Rab11 representative structures, suggesting the reliability of the method in detecting known binding sites.

FTMap also identified some novel binding sites in the representative structures. Residues of RabF1 (residues 44–48), RabF2 (residues 61–65), RabF3 (residues 71–77), RabF4 (residues 80–84), RabSF1 (residues 8–13), RabSF2 (residues 25–42), RabSF3 (residues 100–122) are observed to form binding sites in most of the Rab11 structures (S4 Table). In all representative structures, residues of the RabSF3 (residues 100–122) region, form binding sites. By visually examining FTMap results using PyMOL, we observed some binding pockets/sites in the representative structures comprising the residues identified by FTMap, which could serve as targets for small molecule drugs. In Rab11 structures crystallized without interacting partners (PDB entries 1OIV_A and 1YZK_A), we observed a small pocket formed by residues Y99, F142 and N-terminal of RabSF3 region (residues V102 and E103) (S13 Fig). In 1YZK_A, we observed another small binding pocket formed by RabSF1 (residues Y8-K13) and the N-terminal of RabSF4 (residues Y173-I175) (S14 Fig). Some residues of RabSF1 form a binding site in many Rab11 structures that are co-crystallized with interacting partners. For example, residues Y10-I17 of Rab11 form a binding site in PDB entries 4C4P_A, 4LX0_C and 5JCZ_D (S15 Fig). We also identified some binding sites in Rab11 that are near to its active site. In PDB entries 1YZK_A, 4C4P_A, 4LX0_C, 4OJK_A, 4UJ5_B, 5C46_F and 5JCZ_D, we observed a binding site formed by residues D19, S20, G69, Q70, E71 and W105, near to the active site (S16–S18 Figs). In PDB entries 1YZK_A and 4UJ5_B, residues G45, T67, A68, R74 and I76 form a binding site (S19 Fig). In 4UJ5_B, we observed a pocket formed by RabSF2 (residues S25-S42) and adjacent residues (residues T43-E47), which is near the active site (S20 Fig). In PDB entry 1OIV_A, two binding sites are identified near the active site that are formed by residues I17, T67, R72, Y80, Y81, W105, E108 and residues N101, R104, W105 and E108 of RabSF3, respectively (S21 Fig).

We compared the binding sites in Rab11 with that of Rab1 representative structures (PDB entries 2FOL_A, 2WWX_A, 3SFV_A, 3TKL_A, 4FMB_D and 4I1O_E) that we have previously identified using FTMap[37]. In Rab1, residues of RabF1, RabF2, RabF3, RabF4, RabSF1, RabSF2 and RabSF3 are observed to form binding sites, as in Rab11. There are many similarities in the occurrence of binding sites in Rab1 and Rab11 structures, i.e. amino acids at equivalent positions in these protein structures are identified as binding sites by FTMap. RabSF3 (residues 100–122) form binding site in all Rab1 structures, as in Rab11. In Rab1 structures 2FOL_A, 2WWX_A and 4I1O_E, residues K10-S17 form binding sites as observed in Rab11 (S22–S24 Figs). In Rab1 structures 3TKL_A, 4FMB_D and 4I1O_E, residues 99, 102, 103 and 142 (F99, V102, K103, F142 in 4FMB_D and 3TKL_A; V99, W102, L103 and S142 in 4I1O_E) form a binding pocket as in Rab11 structures 1OIV_A and 1YZK_A (S25–S27 Figs). Residues N101, Q104, W105 and E108 of RabSF3 in Rab1 structures 3SFV_A and 3TKL_A form binding sites as observed in Rab11 structure 1OIV_A (S28 Fig). In Rab1 structures 2WWX_A, 3SFV_A, and 4I1O_E, a small binding pocket is formed by residues 8–13 (L8-L13 in 2WWX_A and 4I1O_E; Y8-K13 in 3SFV_A) and the residues in the RabSF4 region, as in Rab11 structure 1YZK_A (S29 Fig). In Rab1 structure 2FOL_A, a unique small binding site was previously observed at the C-terminal of helix 1 (residues L25-D31), which is a part of RabSF2 (residues 25–42) [37]. Interestingly, this binding site is not observed in Rab11 representative structures (S30 Fig).

In brief, we observed that many pockets are structurally conserved in Rab1 and Rab11 even though the amino acids that form the pockets are poorly conserved at the sequence level. Previous studies have shown that protein structures are more related to functions and can also be conserved in the absence of high sequence conservation [57][58]. This is because proteins accept mutations of surface residues more readily than mutations of buried residues and as a result closely related proteins differ mainly in surface residues [58]. Large structural changes are usually produced by the mutations of internally buried residues than that of the surface residues [58]. Even though the binding sites in Rab11 and Rab1 are structurally conserved, the unique residues in their binding sites, that are part of the RabSF regions may allow in specific regulation of these proteins.

### Virtual screening using Rab11 representative structures

**Fig 2:**
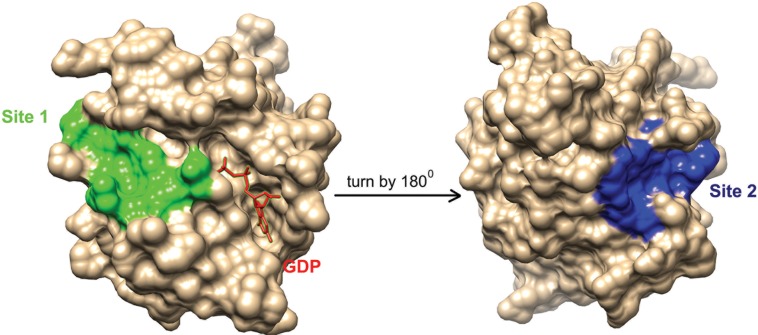
Site 1 and Site 2 in Rab11. The figure shows site 1 (colored in green) and site 2 (colored in blue) in Rab11 representative structure 1OIV_A. Guanosine-5'-Diphosphate (GDP) in the active site near site 1 is shown as red sticks. The Rab11 structure is rotated by 180^0^ along the Y axis to show the location of site 2. The figures are generated using UCSF Chimera [65].

**Fig 3:**
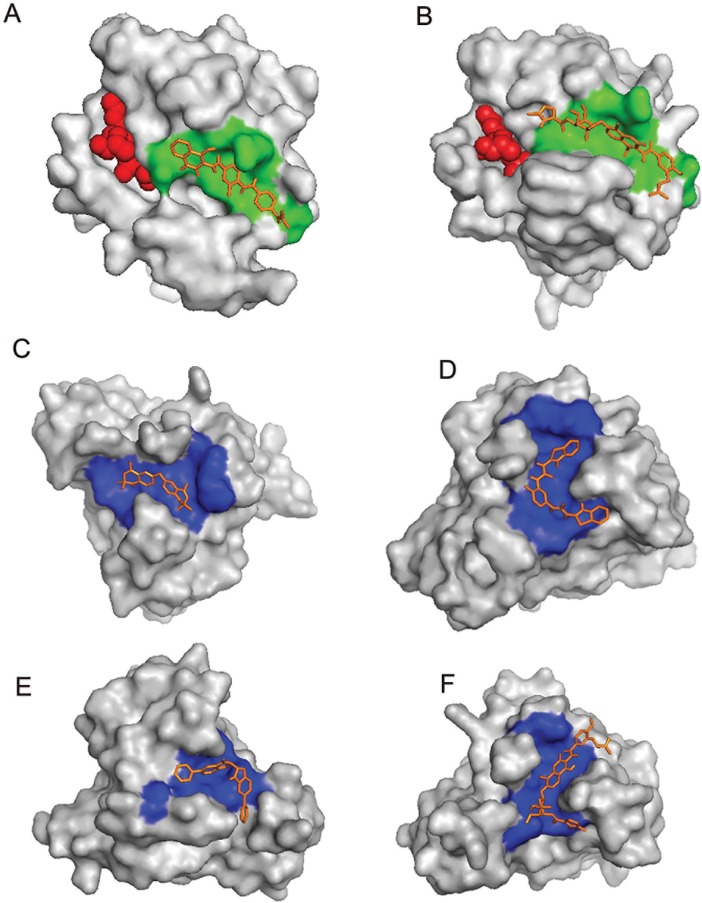
Top scoring ligands docked at different Rab11 representative structures. (A) ZINC18141294 docked at site 1 of PDB entry 1OIV_A, (B) ZINC29590259 docked at site 1 of PDB entry 4LX0_C, (C) ZINC04773602 docked at site 2 of PDB entry 1YZK_A, (D) ZINC01572309 docked at site 2 of PDB entry 4C4P_A, (E) ZINC13152284 docked at site 2 of PDB entry 4UJ5_B and (F) ZINC29590263 docked at site 2 of PDB entry 4OJK_A. Ligands are shown in brown sticks. Site 1 and Site 2 are colored in green and blue, respectively. Ligands at the active sites of Rab11 in (A) and (B) are shown as red spheres.

**Fig 4:**
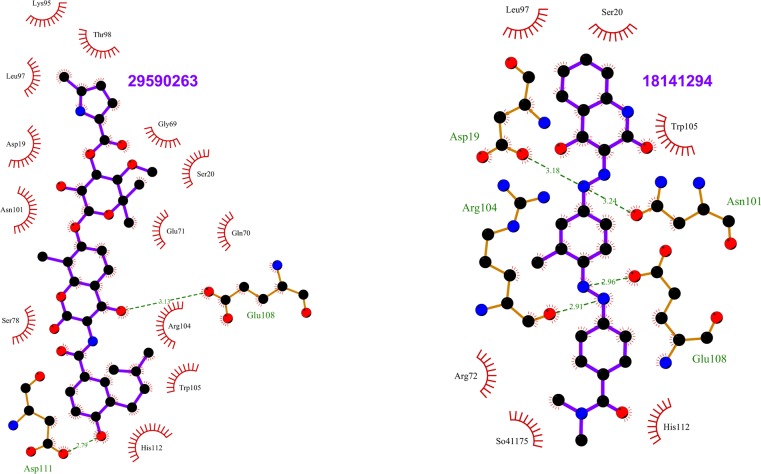
Top scoring ligands ZINC29590263 and ZINC18141294 and their interactions with Rab11. The figure shows the interactions of the ligands (labeled) with the residues of the Rab11 structures in which they scored the best. ZINC29590263, ZINC29590257, and ZINC29590259 are isomers. The ligand and Rab11 side chains are shown in ball-and-stick representation. Black circles denote carbon atoms, red circles denote oxygen atoms and blue circles denote nitrogen atoms. The ligand bonds are colored in purple. Residues in Rab11 interacting with the ligand are labeled. Hydrogen bonds are shown as green dotted lines. The Rab11 residues making nonbonded contacts with the ligand are shown as spoked arcs. Figures are generated using LigPlot+ [66][67].

**Fig 5:**
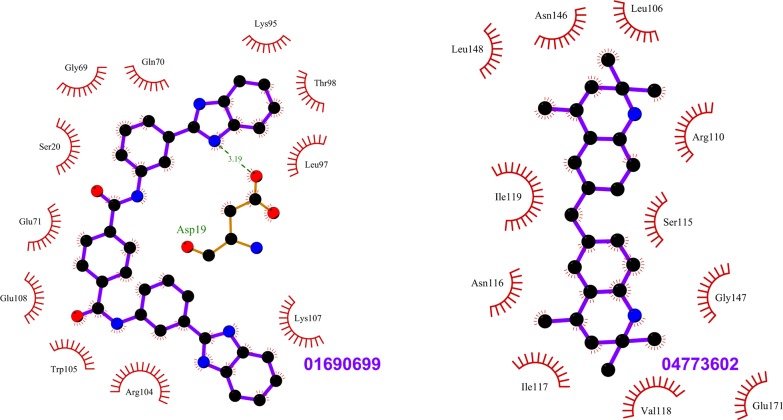
Top scoring ligands ZINC01690699 and ZINC04773602 and their interactions with Rab11. The figure shows the interactions of the ligands (labeled) with the residues of the Rab11 structures in which they scored the best. The ligand and Rab11 side chains are shown in ball-and-stick representation. Black circles denote carbon atoms, red circles denote oxygen atoms and blue circles denote nitrogen atoms. The ligand bonds are colored in purple. Residues in Rab11 interacting with the ligand are labeled. Hydrogen bonds are shown as green dotted lines. The Rab11 residues making nonbonded contacts with the ligand are shown as spoked arcs. Figures are generated using LigPlot+ [66][67].

**Fig 6:**
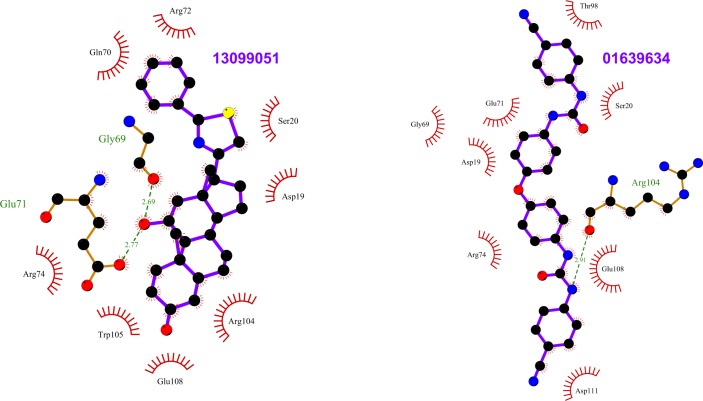
Top scoring ligands ZINC13099051 and ZINC01639634 and their interactions with Rab11. The figure shows the interactions of the ligands (labeled) with the residues of the Rab11structures in which they scored the best. The ligand and Rab11 side chains are shown in ball-and-stick representation. Black circles denote carbon atoms, red circles denote oxygen atoms, yellow circle denoted sulfur atom and blue circles denote nitrogen atoms. The ligand bonds are colored in purple. Residues in Rab11 interacting with the ligand are labeled. Hydrogen bonds are shown as green dotted lines. The Rab11 residues making nonbonded contacts with the ligand are shown as spoked arcs. Figures are generated using LigPlot+ [66][67].

**Fig 7:**
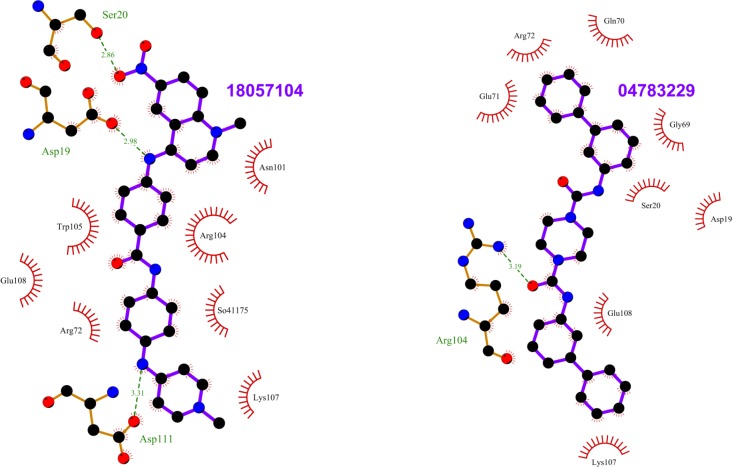
Top scoring ligands ZINC18057104 and ZINC04783229 and their interactions with Rab11. The figure shows the interactions of the ligands (labeled) with the residues of the Rab11 structures in which they scored the best. The ligand and Rab11 side chains are shown in ball-and-stick representation. Black circles denote carbon atoms, red circles denote oxygen atoms, and blue circles denote nitrogen atoms. The ligand bonds are colored in purple. Residues in Rab11 interacting with the ligand are labeled. Hydrogen bonds are shown as green dotted lines. The Rab11 residues making nonbonded contacts with the ligand are shown as spoked arcs. Figures are generated using LigPlot+ [66][67].

**Fig 8:**
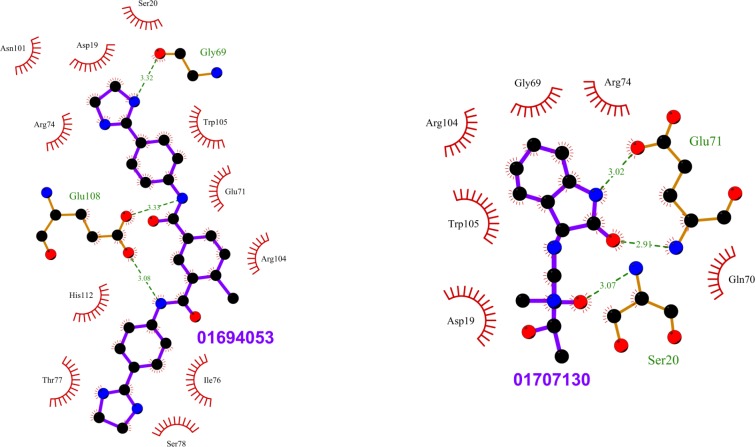
Top scoring ligands ZINC01694053 and ZINC01707130 and their interactions with Rab11. The figure shows the interactions of the ligands (labeled) with the residues of the Rab11 structures in which they scored the best. The ligand and Rab11 side chains are shown in ball-and-stick representation. Black circles denote carbon atoms, red circles denote oxygen atoms, and blue circles denote nitrogen atoms. The ligand bonds are colored in purple. Residues in Rab11 interacting with the ligand are labeled. Hydrogen bonds are shown as green dotted lines. The Rab11 residues making nonbonded contacts with the ligand are shown as spoked arcs. Figures are generated using LigPlot+ [66][67].

**Fig 9:**
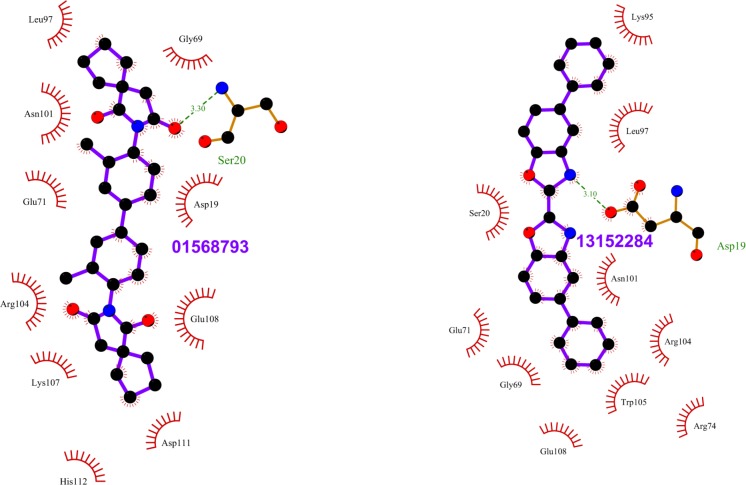
Top scoring ligands ZINC01568793 and ZIN13152284 and their interactions with Rab11. The figure shows the interactions of the ligands (labeled) with the residues of the Rab11 structures in which they scored the best. The ligand and Rab11 side chains are shown in ball-and-stick representation. Black circles denote carbon atoms, red circles denote oxygen atoms, and blue circles denote nitrogen atoms. The ligand bonds are colored in purple. Residues in Rab11 interacting with the ligand are labeled. Hydrogen bonds are shown as green dotted lines. The Rab11 residues making nonbonded contacts with the ligand are shown as spoked arcs. Figures are generated using LigPlot+ [66][67].

Compounds from National Cancer Institute (NCI) diversity subset 3 were docked, against the RabSF3 region, in all Rab11 representative structures using AutoDockVina[59]. We have focused on the RabSF3 region for docking studies, since many residues in this region form binding sites in all Rab11 representatives and the residues in this region have correlated motions with the residues near the switch 2 region (Y80-W105). During the virtual screening, we observed that ligands prefer two distinct cavities/pockets in the RabSF3 region (referred to as site 1 and site 2 hereafter) (Fig 2). The range of free energy predicted by AutoDockVina for all compounds lie between -2 Kcal/mol and -10 Kcal/mol.Next, we re-scored the top scoring ligands identified by AutoDockVina[59], based on their observed interactions with Rab11 representatives, using Protein-Ligand Interaction Profiler (PLIP) [60]. The resultant 15 top scoring ligands and their respective targets are listed in Table 1. These ligands exhibit interactions with the residues in and near the RabSF3 region (see Table 1; Figs 3–9 and S3 File). Some of the ligands are scored best only in certain conformations of Rab11. The ligands that are scored best only in the inactive conformation of Rab11a (i.e. GDP-bound Rab11; PDB entry 1OIV_A) are listed in Table 2 and are shown in S31–S33 Figs. The ligands that are scored best only in GNP-bound Rab11a (PDB entries 1YZK_A, 4C4P_A and 4UJ5_B) are listed in Table 3 and are shown in S34–S37 Figs. The ligands that are scored best only in Rab11b (PDB entry 4OJK_A) are listed in Table 4 and are shown in S38 Fig. Finally, we redocked the top hits using Vinardo[61]. Vinardo is a scoring function implemented in smina[62], a fork of AutoDockVina. Vinardo has shown to have superior docking capabilities compared to Vina[61]. During redocking, some of the top scorers in the initial docking procedure were not observed as hits for certain Rab11 representatives (Table 1). However, all the Vina top scorers were reported as hits for the target structures in which they scored the best during the initial docking (Tables 1–4). The free energy values computed by Vinardo for the top scorers are listed in S5–S8 Tables. While absolute scores given by the different scoring methods are not comparable [39], consensus ligands predicted by both Vina and Vinardo suggest the robustness of our proposed compounds targeting the allosteric sites of Rab11. We observed that many of the top scoring ligands meet Lipinski's rule of five [63][64] (Table 5).Structurally, most of the top scored ligands are characterized by nitrogen-rich scaffolds and aromatic rings. However, their structures are so diverse that it is impossible to find a substructure common to all of them.

**Table 1:** Top scoring ligands identified through virtual screening.

Ligand	Target structure	Site	Free energy (Kcal/mol)	Interactions
ZINC29590259	4LX0_C^*^	Site 1	-8.9	Hydrogen bonds-ASP19, SER20, ASN101, ARG104, GLU108, ASP111, HIS112
	5C46_F^*^	Site 1	-8.2	Hydrophobic interactions- TYR99, VAL102, GLU103, ARG104, LYS107, PHE142 Hydrogen bonds-ARG74, GLU100, ARG104 Salt bridge- LYS107
	1OIV_A^*^	Site 1	-8.5	Hydrophobic interactions- ARG74, THR77 Hydrogen bonds- SER20, ARG72 π-Cation Interaction- HIS112
	4OJK_A^*^	Site 2	-8.4	Hydrophobic interactions-GLU103, LEU106 Hydrogen bonds-LEU148, GLU171
ZINC29590263	4LX0_C^*^	Site 1	-9	Hydrogen bonds- ASP19, SER20, ARG104, GLU108, ASP111, HIS112
	4OJK_A	Site 2	-8.3	Hydrophobic interactions- GLU103, LEU106 Hydrogen bonds- LEU148, GLU171
ZINC18141294	1OIV_A^*^	Site 1	-8.9	Hydrogen bonds-SER20, ASN101, ARG104, GLU108, HIS112
	4OJK_A^*^	Site 2	-8	Hydrogen bonds- ILE117, ILE119, ASN146, GLU171
ZINC01690699	4LX0_C^*^	Site 1	-9.9	Hydrogen bonds- ASP19, GLU108
	1OIV_A	Site 1	-8.6	Hydrogen bonds- ASP19, SER20, GLU108, ASP111, HIS112 π-Cation Interaction- HIS112
	4C4P_A	Site 2	-8.6	Hydrogen bonds-ALA113, SER115, ILE119, GLY147 π-Cation Interaction- HIS112
	5C46_F^*^	Site 1	-8.7	Hydrogen bonds- ARG74, GLU100, ARG104
	4UJ5_B^*^	Site 2	-8.9	Hydrogen bonds-ILE117, ASN146
	1YZK_A^*^	Site 2	-8.6	Hydrogen bonds- ALA113, ILE119, GLY147
	5JCZ_D^*^	Site 1	-8.3	Hydrogen bonds- ASP19, ASN101, GLU103, ARG104
ZINC04773602	1YZK_A^*^	Site 2	-8	Hydrophobic interactions- LEU106, LEU109, ARG110, VAL118, ILE119, ASN146, LEU148, GLU171, ILE175 Hydrogen bond- ILE119
ZINC29590257	5C46_F^*^	Site 1	-8.2	Hydrophobic interactions- TYR99, VAL102, LYS107, PHE142 Hydrogen bonds- ARG74, GLU100, GLU103, ARG104 Salt bridge- LYS107
	4LX0_C^*^	Site 1	-8.7	Hydrogen bonds- ASP19, ARG74, ASN101, ARG104
	4OJK_A^*^	Site 2	-8.4	Hydrophobic interactions-GLU103, LEU106 Hydrogen bonds- ASN147, SER149
ZINC13099051	4LX0_C^*^	Site 1	-9.4	Hydrophobic interactions-GLU71 Hydrogen bonds- GLY69, GLU71, ARG74, GLU108
	1OIV_A^*^	Site 1	-8.6	Hydrophobic interactions- LYS107, GLU108 Hydrogen bond- LYS107
ZINC01639634	4LX0_C^*^	Site 1	-8.6	Hydrogen bonds- SER20, GLY69, GLU71, ARG104, GLU108
ZINC18057104	1OIV_A^*^	Site 1	-9.1	Hydrogen bonds- ASP19, SER20, GLU108, ASP111 Salt bridge- ASP19, ASP111
	4LX0_C^*^	Site 1	-8.8	Hydrogen bonds- SER20, ARG104, GLU108 Salt bridges- GLU108
	4UJ5_B^*^	Site 2	-8.3	Hydrogen bonds- ASN146, GLU171
ZINC04783229	4LX0_C^*^	Site 1	-9.6	Hydrogen bonds-GLY69, ARG104, GLU108
	4OJK_A^*^	Site 2	-9	Hydrogen bonds-ILE117
	4UJ5_B^*^	Site 1	-8.5	Hydrogen bonds- ASP19, ASN101
	4C4P_A	Site 2	-8.4	Hydrogen bonds- ILE117, GLU171
ZINC01694053	4LX0_C^*^	Site 1	-8.6	Hydrogen bonds- SER20, GLY69, ARG74, THR77, SER78, GLU108
	4C4P_A^*^	Site 2	-8.3	Hydrogen bonds- ALA113, SER115, ILE1117, ILE119
ZINC01572309	1YZK_A^*^	Site 2	-8.8	Hydrogen bonds-ARG110, SER115, ASN146, GLY147
	4C4P_A^*^	Site 2	-8.3	Hydrogen bonds- SER115, ASN116, GLY147, SER149, GLU171
	4OJK_A^*^	Site 2	-8.4	Hydrogen bonds- SER115, ASN116, ASN147, SER149, GLU171
	1OIV_A^*^	Site 1	-8.6	Hydrogen bonds- ASP19, ARG104, GLU108
ZINC01707130	4LX0_C^*^	Site 1	-8.5	Hydrogen bonds- ASP19, SER20, GLU71 Salt bridge- ASP19
ZINC01568793	4LX0_C^*^	Site 1	-9	Hydrophobic interactions- LEU97 Hydrogen bonds- SER20, ASN101 Salt bridges- ASP19, GLU108
	5JCZ_D^*^	Site 1	-8.6	Hydrophobic interactions- THR77, GLU103 Hydrogen bonds- ARG104 π-Cation Interaction- ARG104
	1OIV_A^*^	Site 1	-8.5	Hydrophobic interactions- LEU97 Hydrogen bonds- SER20, ARG72 π-Cation Interactions- ARG72 Salt bridges- ASP19, GLU108
	4C4P_A^*^	Site 2	-8.3	Hydrophobic interactions- ARG110, ILE117 Salt bridge- GLU171
	4UJ5_B^*^	Site 2	-8	Hydrophobic interactions- LYS107, VAL118 Hydrogen bonds- ILE119
ZINC13152284	4LX0_C^*^	Site 1	-9.2	Hydrogen bonds- ASP19, SER20, GLY69
	1OIV_A^*^	Site 1	-9.1	Hydrogen bond -104
	4UJ5_B^*^	Site 2	-8.3	Hydrogen bond- GLU171

Top scoring ligands, their target sites in each representative structure for which they scored best, their binding free energy values predicted by AutoDockVina, and their interactions with the respective target structures that are observed using protein-ligand interaction profiler (PLIP) are listed. Ligands are listed based on their weighted scores (see methods). The target structures for which, each ligand scored best is indicated in bold. The ligand structures are shown in Figs 4–9.

* The target structures for which the ligands are observed as hits duringredocking using Vinardo.

**Table 2:** Ligands that are scored best only in GDP-bound Rab11a (PDB entry 1OIV_A).

Ligand	Site	Free energy computed by AutoDockVina (Kcal/mol)	Interactions
ZINC00084617	Site 1	-8.6	Hydrogen bonds- ASN 101, TRP 105 Salt bridges—ARG104, GLU108
ZINC01578333	Site 1	-8.9	Hydrogen bonds- ASN101, Salt bridge- ASP19
ZINC13208966	Site 1	-8.9	Hydrophobic interactions- ARG104 Hydrogen bonds- ARG104
ZINC04720972	Site 1	-8.6	Hydrogen bonds-ASN101, TRP105
ZINC11677172	Site 1	-8.6	Hydrophobic interaction- GLU108, Hydrogen bonds- SER20, ARG72
ZINC00393674	Site 1	-8.5	Hydrogen bonds-ASN101, ARG104, GLU108
ZINC01701460	Site 1	-8.8	Hydrogen bond-ASN 101 π-Cation Interaction- ARG72

The target sites of ligands in 1OIV_A, their free energy of binding and their interactions with Rab11 that are observed using protein-ligand interaction profiler (PLIP) are listed. The listed ligands were also reported as hits for the respective target structures while redocking. The ligand structures are shown in S31–S33 Figs. GDP stands for Guanosine-5'-Diphosphate.

**Table 3:** Ligands that are scored best only in GNP-bound conformations of Rab11a.

Ligands	Targets	Site	Free energy computed by AutoDockVina (Kcal/mol)	Interactions
ZINC04773602	**1YZK_A**	Site 2	-8	Hydrophobic interactions- LEU106, LEU109, ARG110, VAL118, ILE119, ASN146, LEU148, GLU171, ILE175 Hydrogen bond- ILE119
ZINC15952559	**4C4P_A**	Site 2	-8.3	Hydrogen bonds-ILE117, ILE119, SER149
ZINC11677178	**4C4P_A**	Site 2	-8	Hydrophobic interactions- VAL118, GLU171 Hydrogen bonds- ASN116, ILE117, SER149
ZINC12671898	**4C4P_A**	Site 2	-8	Hydrophobic interactions- LEU106, ARG110,ILE119 Hydrogen bonds- ILE119, ASN146 Salt bridge- ARG110
ZINC17353914	**4UJ5_B**	Site 1	-8.2	Hydrophobic interactions- ASP19, LEU70 Hydrogen bonds- SER20, GLU108 Salt bridge- GLU108
ZINC01573829	**1YZK_A**	Site 2	-8.3	Hydrogen bonds- ILE119, GLY147
	4C4P_A	Site 2	-8	Hydrogen bonds- SER115
ZINC01577889	**1YZK_A**	Site 2	-8.4	Hydrogen bond- GLY147
ZINC29590275	**4UJ5_B**	Site 1	-8	Hydrogen bond-TRP105, GLU108
ZINC01726776	**1YZK_A**	Site 2	-8.1	Hydrogen bond- ILE117

The target sites of ligands, their free energy of binding and their interactions with Rab11 that are observed using protein-ligand interaction profiler (PLIP) are listed. Target structures for which the ligands scored best are indicated in bold. The listed ligands were also reported as hits for the respective target structures while redocking. The ligand structures are shown in S34–S37 Figs. GNP stands for Phosphoaminophosphonic acid-guanylate ester.

**Table 4:** Ligands that are scored best only in GDP-bound Rab11b (PDB entry 4OJK_A).

Ligands	Site	Free energy (Kcal/mol)	Interactions
ZINC17465979	Site 1	-8.2	Hydrophobic interactions- ASP19, THR98 Hydrogen bonds- SER20, ASN101, ARG104
ZINC05462670	Site 1	-8.1	Hydrogen bonds- GLY18, SER20, ASN101, ARG104
ZINC05462674	Site 1	-8.1	Hydrogen bonds- GLY18, SER20, ASN101, ARG104
ZINC12672242	Site 1	-8.4	Hydrogen bonds-SER20
ZINC17465983	Site 1	-8.1	Hydrophobic interaction- LEU97 Hydrogen bonds- SER20, ASN101, ARG104
ZINC01668429	Site 2	-8	Hydrogen bonds- GLU103, ASN147, GLU171

The target sites of ligands, their free energy of binding and their interactions with Rab11 that are observed using protein-ligand interaction profiler (PLIP) are listed. The listed ligands were also reported as hits for the respective target structures while redocking. The ligand structures are shown in S38 Fig. GDP stands for Guanosine-5'-Diphosphate.

**Table 5:** Molecular properties of ligands identified in virtual screening.

Ligands	H-bond donors	H-bond acceptors	Molecular mass (g/mol)	LogP
ZINC29590259	4	13	696.129	5.73
ZINC29590263	4	13	696.129	5.73
ZINC18141294	1	9	453.482	4.43
ZINC01690699	4	8	548.606	6.89
ZINC04773602	2	2	358.529	6.03
ZINC29590257	4	13	696.129	5.73
ZINC13099051	1	3	447.644	5.66
ZINC01639634	4	9	488.507	5.61
ZINC18057104	3	9	506.566	5
ZINC04783229	2	6	476.58	6.47
**ZINC01694053**	6	8	488.979	2.06
ZINC01572309	4	8	474.571	5.84
**ZINC01707130**	3	8	299.29	-0.28
**ZINC01568793**	0	6	484.596	4.28
ZINC13152284	2	4	392.458	6.51
**ZINC00084617**	1	6	333.323	2.71
**ZINC13208966**	2	2	275.351	3.91
ZINC04720972	3	5	382.419	6.01
**ZINC11677172**	2	6	496.575	3.78
**ZINC00393674**	2	5	314.348	3.11
ZINC01701460	0	2	367.279	7.26
ZINC04773602	2	2	358.329	6.03
**ZINC15952559**	3	5	271.251	3.07
**ZINC11677178**	2	6	496.575	3.78
**ZINC12671898**	0	4	412.57	4.70
**ZINC17353914**	2	5	472.629	4.96
ZINC01573829	0	3	353.45	5.98
ZINC01577889	2	4	392.458	5.41
ZINC29590275	1	3	447.003	6.38
ZINC01726776	0	5	398.853	5.98
ZINC17465979	6	10	546.528	5.26
ZINC05462670	6	10	546.528	5.26
ZINC05462674	6	10	546.528	5.26
ZINC12672242	2	3	410.524	5.61
ZINC17465983	6	10	546.528	5.26
ZINC01668429	6	11	499.531	1.34

For each ligand, number of groups that can act as hydrogen bond donors (H-bond donors), number of groups that can act as hydrogen bond acceptors (H-bond acceptors), molecular mass and lipophilicity (expressed as LogP value) are listed. Ligands that meet Lipinski's rule of five are indicated in bold.

Site 1 is near the active site of Rab11 and is formed by residues in the N-terminal region of helix V102-H112 and loop G18-S29 in Rab11. Site 2 is distant from the active site and is formed by residues in the C-terminal region of helix V102-H112 and loops K145-S149 and D114-I117. We observed that the top scoring ligands (i.e. the consensus top scorers listed in Table 1) shows different binding affinities for these sites in different representative structures. In representative structures 1OIV_A, 4LX0_C, 5C46_F and 5JCZ_D, the top scoring ligands prefer site 1 and in representative structures 1YZK_A, 4C4P_A, 4OJK_A, the top scoring ligands prefer site 2. In PDB entry 4UJ5_B, we got hits for both site 1 and site 2. This preferential binding of ligands in the representative structures of Rab11 can be explained by conformational variances of site 1 and site 2 (S39 and S40 Figs).

In PDB entries 1OIV_A and 4LX0_C, we observed that the top scoring ligands are closely packed against residues D19, S20, N101, R104, W105, E108, D111 lining the site 1. In PDB entries 5C46_F and 5JCZ_D, residue R104 undergoes conformational change so as to block the entire access of the pocket/site1 by ligands (S41 Fig). In 5C46_F, in addition to the residues of site 1, ligands seem to interact with a nearby narrow cavity lined by residues Y99, E100 and F142 (S41 Fig). In PDB entries 1YZK_A, 4C4P_A, 4OJK_A and 4UJ5_B, the top scorers are closely packed against residues L106, L109, R110, A113, I117, V118, I119, L148, S149, E171 that lines site 2.

We observed that most of the ligands that are scored well only in GDP-bound Rab11a have hydrogen bonds with N101 of 1OIV_A. It was also interesting to note that while the consensus top scoring ligands prefer site 1 in GDP-bound Rab11a (PDB entry 1OIV_A), they prefer site 2 in GDP-bound Rab11b (PDB entry 4OJK_A), which is co-crystallized with an interacting partner. On the other hand, most of the ligands that are scored best only in the Rab11b structure (Table 4) bind to site 1. Since these ligands have hydrogen bonds with residues S20, N101 and R104 in 4OJK_A, we infer that these residues are important for the binding of ligands at site 1 in Rab11b.

### Switch 2 communicates with sites 1 and 2

**Fig 10:**
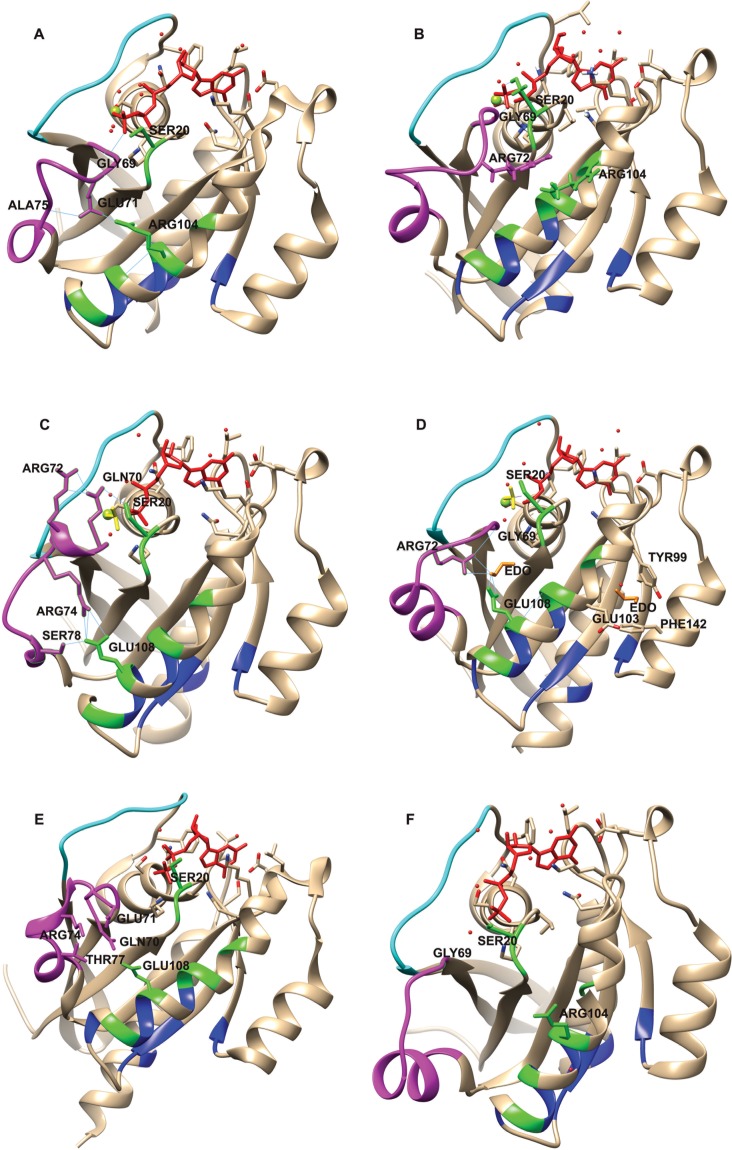
**Communications between switch 2 and site 1 in (A) 1YZK_A, (B) 4C4P_A, (C) 4LX0_C, (D) 5JCZ_D, (E) 4OJK_A and (F) 1OIV_A.** Switch 1 and switch 2 regions are shown in cyan and magenta, respectively. Site 1 and site 2 residues are colored in green and blue, respectively. The ligands at the active site (i.e. GTP analogs or GDP) are shown as red sticks. Berryliumflouride (BEF) in (C) and (D) are shown as yellow sticks. In (D) ethylene glycol (EDO) molecules are shown as orange sticks. For each structure, the magnesium ion at the active site is represented as a green sphere, water molecules are shown as red spheres and the residues that undergo conformational changes are labeled. Hydrogen bonds are shown as thin blue lines. In (A) GLY69 of switch 2 has hydrogen bond interaction with the ligand. Switch 2 is perturbed and GLU71 (switch 2) makes contacts with ARG104 (site 1) and ALA75 (switch 2), through hydrogen bonds. In (B) GLY69 and SER20 have hydrogen bond interactions with the ligand. The interaction with Rab11a-effector causes additional conformational changes in switch 2 region comprising ARG72, and there are associated conformational changes in residue ARG104 of site 1. In (C), in relation to switch 2 conformational changes, ARG72 and SER20 have hydrogen bonds with GLN70, and ARG74 and SER78 have hydrogen bonds with GLU108. In (D), there are two EDO molecules shown on protein structure. ARG72 and GLU108 have hydrogen bond interactions with EDO. ARG72 has hydrogen bond interaction with GLY69. In (E), there are conformational changes in GLN70, GLU71, ARG74, THR77 and GLU108 associated with perturbation of switch 2. In (F), the GDP-bound representative structure of Rab11a is shown, the switch 2 region is structurally different from the other representative structures and it is in no position to have hydrogen bond interactions with site 1. Figures are generated using UCSF Chimera [65].

To probe the possible reasons for the conformational variances in site 1 and site 2, we examined the interactions in the crystal structures of Rab11 representative structures using UCSF Chimera [65] and RING-2.0 server [68]. We observed that the switch 2, which is highly flexible in Rab11 structures, have conformational variations in the representatives dependent on the presence of nucleotide in the active site, and the interactions with effectors (the effectors and the structural changes induced by them on Rab11 are detailed in Discussion section). In each Rab11 representative with GTP analog at the active site, there is an additional residue (GLY 69) from switch 2 interacting with the γ-phosphate of the GTP analog compared to GDP-bound Rab11 (S42 Fig). We infer that in Rab11, either this γ-phosphate stabilizes the different conformations of switch 2 in the GTP analog-bound Rab11, or induces the different conformations, upon ligand binding. Additionally, the effectors of Rab11 interacts mainly with the switch regions of Rab11, perturbing the switch 2. However, the perturbations in switch 2 promote interactions between residues in switch 2 and residues in helix V102-H112 (that comprises the residues of site 1 and site 2), and there are associated conformational changes in residues involved in the interaction (Fig 10). Additionally, we observed that residue SER20 (site 1) in these representative structures have different conformations dependent on interactions with the active site ligand, the presence of a water molecule in the active site, or switch 2 perturbations (Fig 10).

In the Rab11 representative 1YZK_A, having GNP at the active site, we observed that the residue GLY69 from switch 2 region interacts with the GNP, and the switch 2 region comprising residue GLU71, lies near to site 1 residues in the helix V102-H112 (Fig 10A). Residue GLU71 involves in hydrogen bonding contacts with residue ARG104 in helix V102-H112 and ALA75 from switch 2. The conformation of ARG104 is perturbed with respect to that in the GDP-bound representative 1OIV_A (Fig 10F). Residue SER20 from site 1 in this structure has interactions with a water molecule in the active site and the residue points towards switch 2.

In PDB entry 4C4P_A (GNP-bound; co-crystallized with an interacting partner), in addition to the nucleotide-dependent conformational changes, the interacting protein perturbs switch 2. As a result of these perturbations, residue ARG72 from switch 2 involves in van der Waals interaction with ARG104 (Fig 10B) and conformational changes are observed in ARG104 with respect to that in the other structures. Residue SER20 has hydrogen bond with the GNP.

PDB entry 4LX0_C (co-crystallized with an interacting partner) has beryllium fluoride (BEF) and GDP at the active site. BEF acts on G proteins as analogue of the γ- phosphate of GTP [69]. The switch 2 in this structure has interactions with BEF, and with the effector, and is perturbed. In this structure, residue GLN70 of switch 2 forms hydrogen bonds with residues SER20 of site 1 and ARG 72 of switch 2, and residue GLU108 of site 1 form hydrogen bonds with residues ARG74 and SER78 of switch 2 (Fig 10C).

PDB entry 5JCZ_D (co-crystallized with an interacting partner) also has BEF and GDP at the active site. The switch 2 in this structure is also perturbed as in the above mentioned structures. This structure has two ethylene glycol (EDO) molecules (EDO is used as a precipitant and cryoprotectant during crystallization and data collection experiments [70][71]) on two shallow cavities (Fig 10D) near the helix V102-H112. In this structure, residues ARG72 from switch 2 and GLU108 from site 1 have hydrogen bond interactions with one of the EDO molecules. Residue SER 20 in this structure points towards the GDP.

In PDB entry 4OJK_A, which is the only Rab11b structure among the Rab11 representatives, the switch 2 region is different from the switch 2 regions of the other Rab11a structures. 4OJK_A interacts with its effector in the GDP-bound state. The switch 2 in this structure has no interactions with the bound nucleotide. However, as a result of its interaction with the effector, the residues GLU71, GLN70, ARG74, THR77 from switch 2 and GLU108 from site 1 comes together in space and involves in van der Waals interactions, causing conformational changes on protein surface (Fig 10E). Residue SER 20 points towards the GDP in this structure.

PDB entries 1OIV_A (Fig 10F), 4UJ5_B (Panel A in S43 Fig) and 5C46_F (Panel B in S43 Fig) are different from the other representatives in that, the communication between switch 2 and helix V102-H112 are minimum. 1OIV_A has GDP at the active site; there are no residues from switch 2 involved in hydrogen bond interactions with the GDP (S42 Fig). 4UJ5_B and 5C46_F have GTP analogs at the active site, and they are crystallized with interacting partners. Even though the switch 2 regions are perturbed in these structures (S43 Fig), they are in no position to have direct hydrogen bonding interactions with site 1. However, residues ARG104 and SER20 have different conformations in these structures (S43 Fig).

Taken together, these observations suggest that the different conformations of the switch 2 region affect the architecture of sites 1 and 2, and the Rab11 effectors induces unique conformational changes at these sites via switch 2. These communications of the novel sites with the switch 2 also provides an evidence for their allosteric character.

### Site 1 is conserved in Rab1 and Rab11

On examining the Rab1 representative structures, we observed that site 1 and site 2 are structurally conserved in Rab1. For example, RMSD deviations of these sites in Rab1 representative 3TKL_A from that of Rab11 representative 1OIV_A are less than 1 Å. In order to know whether the top scoring ligands identified for Rab11 also binds to Rab1, we docked the top scoring ligands against the RabSF3 region of PDB entry 3TKL_A. We observed that all the top scoring ligands binds to site 1 in Rab1 with high binding affinities. Interestingly, none of the top scoring ligands binds to site 2 in Rab1.

**Fig 11:**
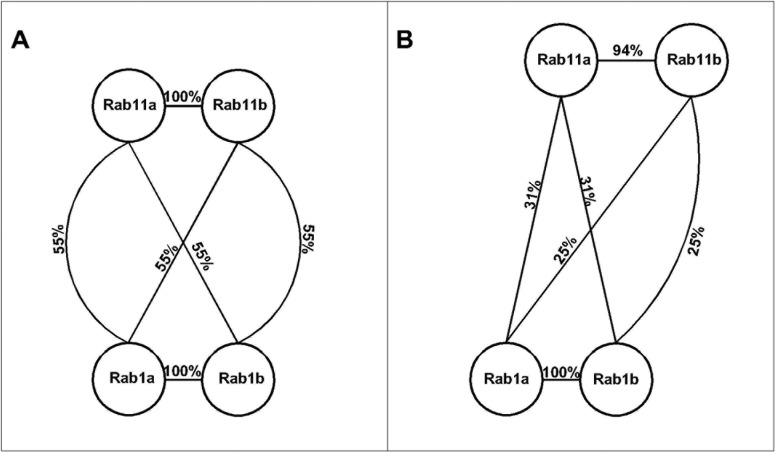
**Sequence map showing identities of (A) site 1 and (B) site 2 between Rab1 and Rab11 proteins.** Proteins are represented as circles and the identities between them are indicated on the edges. Site 1 is conserved in Rab1 and Rab11 proteins having more than 50% identity. Site 2 is less conserved with less than 40% identity.

In order to examine the possible reason for the preference of top scoring ligands for site 1 in Rab1, we performed sequence comparisons of Rab1 and Rab11 by computing their sequence identity. We compared Rab1 and Rab11 at the level of full sequence and for residue contacts made by the top scoring ligands at site 1 and site 2 (see Methods). Many previous studies have shown that ligand binding pockets in different proteins can have much higher conservation than full proteins [72][73]. Consistent with this result, we observed that while the full sequences of Rab1 and Rab11 have less than 50% identity (S9 Table), site 1 in Rab1 is 55% identical to that of Rab11 (Fig 11 and panel A in S44 Fig). On the other hand, residues of site 2 are less conserved in Rab1 and Rab11 (only 25–31% identical) (Fig 11 and panel B in S44 Fig). The high sequence identity of site 1 in Rab1 and Rab11 explains why the ligands also prefer site 1 in Rab11. These results also suggest that site 2, which is less conserved at the sequence level in Rab1 and Rab11, may allow selective regulation of Rab11 proteins.

### Analysis of Rab11 representative structures using their residue interaction networks

The representation of proteins as networks of interacting residues is useful for the identification of functionally important residues [74], key residues associated with protein folding [75][76][77], residue contributions to binding free energy in protein-protein complexes [78][79], etc. Atilgan *et al*. have shown previously that there is relation between spatial arrangement of residues and protein dynamics [77]. Previous studies have shown that central residues in residue interaction networks of proteins that are part of the shortest pathways between any two residues are critical to protein function [80][81]. For example, if we consider protein residue interaction networks as information processing network, it is reasonable to assume that perturbations to central residues in the network that are crucial for network communications, could impair the function of protein [82]. These central residues that lie on the shortest pathways between most residue pair in the protein could play important roles in allosteric communication [82][79].Similar to the concept of centrality, it is also useful to explore the correlated movements of residues that lie on the shortest paths in the residue interaction networks. It was shown previously for methionyl-tRNAsynthetase that dynamically cross-correlated set of residues which lies in shortest paths of connected networks play important roles in long distance communication in the protein [83].

In order to analyze the communication between the newly identified binding sites and the active site in Rab11, we examined the residue interaction networks of the representative structures of Rab11. The residue interaction networks were generated using Network Analysis of Protein Structures (NAPS) web server [84]. Then we examined the shortest paths from the point of contacts (i.e. contacting residues) made by the top scoring ligands at site 1 and site 2, to the residues that are in contact with the active site. It can be assumed that the energy generated at any of these sites (i.e. site 1 or site 2) as a result of the binding of small molecules may be dissipated efficiently to the active site in Rab11, if there are short paths connecting them. We observed that the distances along the shortest paths from residue N101 of site 1 in all representative structures, from residues E103, L106, L109, V118 and I119 of site 2 in PDB entries 1YZK_A, 4C4P_A, 4OJK_A, 4UJ5_B, 5C46_F and 5JCZ_D, and from residues L148 and S149 in all representative structures, to the active site (S10 Table) are minimal, i.e. the paths include fewer residues and their lengths are minimal as compared to that of the shortest paths from other residues of site 1 and site 2 to the active site. We also examined the betweenness centrality values of the residues involved in the shortest paths (see Methods). Residue I119 was found to have high betweenness centrality (see Methods) (S11 Table and S45 Fig). We also observed that residues E103, L106, L109, V118 and I119 are directly connected to nodes having high centrality values in the network. Residues E103, L106 and L109 are directly connected to residue W105, residue V118 is connected to residue A87 and residue I119 is connected to residue L89 in the shortest paths. Residues A87, L89 and W105 have high betweenness centrality (S11 Table and S45 Fig). Even though residues L148 or S149 do not have significantly high centrality values, we infer that these residues are also important since they have strong anti-correlated movements with the interswitch region of Rab11 as shown in the DCCM analysis.

Sol *et al*. have previously shown that there are correlations between residue centrality and residue distance to the protein center of mass [79]. Consistent with their results, we observed that these central residues that lie in and near site 1 and site 2 lies near to the protein center of mass. The distances of residues A87, L89, W105 and I119 to the center of mass of Rab11 are less than 6 Å, 5 Å, 12 Å and 9 Å in all representative structures.

In DCCM analysis, we observed that residues Y80-K107 (that comprises A87, L89 and W105) have strong correlated motions with the N-terminal region in Rab11 that comprises the P-loop residues (S1 File). Residue I119 also has correlated motions with residue L89 to which it is directly connected in the shortest paths (S1 File). Residues L148 and S149 have shown strong correlated movements with residue L121 to which they are directly connected. These results supports the observations previously made by Atilgan *et al*. [77]that the shortest path lengths in residue networks and fluctuations of residues are correlated.

Since highly correlated central residues that lie on shortest paths can play key roles in allosteric communication [79][83][82], we infer that residues A87, L89, W105, I119, L148 and S149 are important and mediate the communication between the newly identified sites and the active site in Rab11.

### Site 1 and site 2 are structurally conserved as allosteric pockets in Ras

We compared the newly identified Rab11 allosteric sites to the allosteric sites in Ras protein which we have identified in a previous study [39]. In the previous study, using computational and simulation approaches, we have shown that there are three allosteric pockets (termed pockets p1, p2 and p3) in Ras. In this study, we observed that residues at equivalent positions in structurally aligned Rab11 and Ras structures form binding sites. Site 1 and site 2 in Rab11 are structurally conserved as allosteric pockets p2 and p3, respectively, in Ras (S46 and S47 Figs) with low RMSD values. For example, the RMSD between site 1and pocket p2 is 3 Å and the RMSD between site 2 and pocket p3 is 2.9 Å for Rab11 structure 1OIV_A and H-Ras structure 2Q21_A, respectively. We also observed that there are many other similarities in the conformation and dynamics of Rab11 and Ras.

Pockets p2 and p3 in Ras lie near helix α3 (residues I93-V103 in H-Ras). Pocket p2 is near the nucleotide-binding site in Ras. Pocket p3 lies in the α3-loop 7 region (residues I93-P110 in H-Ras), distant from the nucleotide binding site, and is also lined by residues S136-P140 in H-Ras. We observed that helix V102-H112 that lines site 1 and site 2 in Rab11 structurally aligns with helix α3 in H-Ras, the residues that lines the pocket p2 and p3. Residues D114-I117 that lines site 2 in Rab11 structurally aligns with residues D105- V109 that lines the pocket p3 in H-Ras. Residues K145-S149 that lines site 2 in Rab11 structurally aligns with residues S136-P140 that lines the pocket p3 in H-Ras. While these observations suggest that the binding sites are structurally conserved in Rab11 and Ras, the amino acids that line these binding sites are poorly conserved between them. While the overall sequence identity between Rab11 and H-Ras sequences is 31%, site 1 and site 2 have only 14% and 19% sequence identities to pockets p2 and p3, respectively (S48 and S49 Figs).

In Ras, the relative displacements of helix α2 (residues Y64-G77 in H-Ras) have shown to affect the conformations of the allosteric pockets [39]. Consequently, the pocket p2 was inaccessible in some Ras structures. In Ras, residues from helix α2 comprise residues of its switch 2 (G60-E76 in H-Ras) and align with residues Y73-G86 of switch 2 of Rab11 (S48 Fig). Similar to Ras, the conformations of Rab switch 2 affects accessibility of site 1 and site 2. Previous molecular dynamics simulation studies on Ras have also shown that the helix α2 has correlated motions with α3-loop 7 region (residues I93-P110) [85][86]. In agreement with this finding, it was shown that the α3-loop 7 region can allosterically communicate with switch 2 and stabilize the switch 2 region [86][87]. Interestingly, in this study, we have observed similar correlated motions in Rab11, between the regions formed by residues Y80-W105 (residues near switch 2 in Rab11) and residues E100-V122, which line sites 1 and 2. The residues from these regions in Rab11 align with the helix α2 and α3-loop7 region, respectively, in Ras (S48 Fig).

In summary, these findings suggest that there are many similarities in the structures and dynamics of Rab11 and Ras. The newly identified binding sites in Rab11 are structurally conserved as allosteric pockets in Ras, suggesting that the novel Rab11 binding sites have the potential to function as allosteric switches.

## Discussion

We have employed multiple approaches for analyzing Rab11 structural dataset of 27 structures. Previous studies have individually deployed clustering methods to scrutinize protein structures and/or their conformational landscape. PCA has been successfully used to investigate proteins including actin capping protein [88], insulin-degrading enzyme [89]and insulin receptor [90].ICA has been used to study LAO protein [91], Rab1 protein[37] and protein tyrosine phosphatase 1B [92]. RMSD-based clustering has been applied on the multiple structures of bromo and extra terminal proteins [93]. In this study, we employed the unique set of PCA, ICA, LLE, and RMSD analyses of switch 1, interswitch and switch 2 to gain a better understanding of the Rab11 structures.

According to our analyses, Rab11 structures that are interacting with different proteins tend to cluster separately. While RMSD analysis and ICA could not separate most of the structures co-crystallized with different partner proteins, the three PCs in PCA could separate all such Rab11 structures in different groups. PCA also separated GDP-bound Rab11 structures from the other structures in the Rab11 ensemble. While we have to employ the three PCs to capture most of the variance in Rab11 data, LLE could cluster most of the structures with different partners in different groups with less than three dimensions. Thus, through the employment of these methods, we gained various insights on the Rab11 structural data. To the best of our knowledge, our study is the first one to use this unique combination of methods for cluster analyses and the results from our study suggest that the employment of this combination of methods can provide useful insights during protein structure analyses.

While small GTP binding proteins, including Rab1 and Rab11, share a similarly conserved core [55],subtle differences in sequence can be translated to distinct structural dynamics and functions, as shown for Ras protein [86].Thus, we are also interested to examine the unique residues and regions of Rab11 to understand their functional effects. Switch 1 and 2 regions in Rab11 differ from those of other Rab proteins. In the GDP-bound Rab11, they are involved in the formation of closely packed symmetrical dimers that enable Rab11 to undergo GDP/GTP cycles without recycling to the cytoplasm [55].Pasqualato *et al*. suggest that these switch regions of Rab11 form a novel interface for Rab11 interacting partners [55]. Currently, there are 5 Rab11a structures (PDB entries 1OIW_A, 1OIX_A, 1YZK_A, 1OIV_A, and 1OIV_B) and 1 Rab11b-GppNHp structure (PDB entry 2F9M_A), that were crystallized without any interacting partners in the Rab11 ensemble of 27 structures. PCA, ICA, and LLE co-clustered PDB entries 1OIW_A [55], 1OIX_A [94], 1YZK_A[95], and 2F9M_A[96]in a separate group. Although Rab11b-GppNHp (PDB entry 2F9M_A) co-clustered with Rab11a structures in all the clustering approaches, there are several key differences between the two isoforms in the residues involved in GTP hydrolysis [96]. In Rab11a-GTP (PDB entry 1OIW_A), neither residue S20 from the P-loop nor residue S42 from the switch 1 is in position to interact with γ-P oxygen in contrast to active Rab11b [96], as a result of which differences are observed in the position of residue S40 between these isoforms. According to Pasqualato *et al*. the switch regions of GTP-bound Rab structures can be divided into two structural classes based on the main chain conformation of S40 [55].This residue either points toward the nucleotide as in Rab11a or is flipped by 180° possibly as the result of a steric conflict with the facing residue from the P-loop (residue S20 in Rab11) for binding the γ-phosphate [55]. These alternative conformations of switch 1 determine the shape of a three-dimensional site spanning residues N26–A49 in Rab11, which comprises switch 1, RabF1 and RabSF2 regions. This region may define a novel three-dimensional epitope for the interaction of Rab11 with specific partners [55].

The FIPs belong to an evolutionarily conserved protein family that acts as effectors for many Rab and Arf (ADP-ribosylation factor) GTPases[97]. They bind to Rab11 subfamily members via a highly-conserved C-terminal Rab11-binding domain (RBD) [97] and regulate recycling endosome trafficking [98]. The FIPs are subcategorized into two classes: class I [Rip11 (Rab11 interacting protein), FIP2 and RCP (Rab-coupling protein)] and class II (FIP3 and FIP4) based on their sequences. In PCA, ICA, and LLE, we have seen co-clustering of FIP2-bound Rab11a structures (PDB entries 2GZH, 2GZD and 4C4P) and FIP3-bound Rab11a structures (PDB entries 2HV8, 2D7C and 4UJ3). Previous studies have shown that both FIP2 and FIP3 interact with Rab11a in the same manner [8][6][5][99]. Rab11a binding interface for FIP2 or FIP3 includes residues from switch 1, interswitch, and switch 2 (S2 Table). We observed that the switch 1 region of these structures adopts similar conformations and differs from that of unbound Rab11a-GTP (i.e. crystallized without interacting partner). The Ser40 in these structures flips toward GTP by 180°, and forms hydrogen bonds with ASN26 and GTP. Residues Ser42 and Ser20 also rotate to make hydrogen bonds with GTP [8], as a result of which conformational changes are induced in the switch regions of Rab11a (S50 Fig). Switch 2 of Rab11a moves farther away from the switch 1 to promote the interaction with FIPs. We observed that the switch 1 regions of these Rab11-FIP complexes are similar and switch 2 regions of these structures are highly flexible adopting different conformations. The similarity observed in the FIP2-bound and FIP3-bound Rab11a structures explains the reason for their co-clustering.

The Myosin 5 is a motor protein that transports a wide variety of membrane cargoes, organelles, secretary vesicles, mRNA, and lipids [100][71][101]. It has three isoforms: Myosin 5a, Myosin 5b, and Myosin 5c. Direct interaction between Rab11 and Myosin 5 (Myosin 5a and Myosin 5b) is necessary to target the motor to Rab11 vesicles [102]. In our study, PCA, LLE, and RMSD analysis (using all residues and switch 2) co-clustered Rab11a structures that are co-crystallized with Myosin 5a (PDB entries 5JCZ_A and 5JCZ_D) [71] and Myosin 5b (PDB entries 4LX0_A and 4LX0_C) [9] separately in two groups. Rab11a binds to the Myosin 5 via the residues in and near the switch regions (S2 Table). As a result of these interactions, Rab11a undergoes conformational changes at switch 1 and switch 2 (S51 Fig). The switch 2 helix of Rab11a is extended as a loop with only one turn of a 3_10_ helix, and forms new interactions with switch 1 which will enhance the lifetime of the active form Rab11 [9]. Even though Myosin 5a also induces conformational changes in switch 2 of Rab11a, the conformational rearrangements induced by Myosin 5a and Myosin 5b are different. We also observed that residue S40 in these Rab11a complex structures adopts a conformation as in Rab11-FIP complexes (S51 Fig).

P14KB is a lipid kinase that localizes primarily at Golgi membranes and is enlisted in biological responses that require rapid delivery of membrane vesicles [16]. The synergistic action of Rab11 and P14KB plays key roles in mediating lipid transport, cytokinesis and maintaining lysosomal identity [16].PCA, LLE, and RMSD analysis (using all residues and switch 2) separated Rab11a structures co-crystallized with P14KB (PDB entries 5C46_F, 4D0L_B, 4D0L_D and 4D0L_F) in a single cluster distinct from other clusters. P14KB is different from the above mentioned effectors in that it makes only a single peripheral contact with switch 1 (i.e. with residues F36, L38, E39, S40) and no contacts with switch 2. Thus, Rab11a switch regions remains available to contact Rab11a effectors even after binding with P14KB. Burke *et al*. have shown that theRab11 residues interacting with PI4KB are all conserved among Rab11a and Rab11b, but not among other Rabs that do not bind PI4KB [16]. These Rab11a structures have shown formation of a helical element (residues 70–77) within switch 2 that is not present in unbound Rab11a-GTP (i.e. GTP-bound Rab11a without interacting partner) (S52 Fig). We observed that the conformational changes induced by P14KB in the switch 1 regions are minor and residue S40 in these structures adopts a conformation similar to that in unbound Rab11a-GTP, which is different from that observed in Rab11-FIP complexes.

Rabin8 is a guanine nucleotide exchange factor (GEF) of Rab8 protein [103][99]. Rab11 interacts with Rabin8 to promote the guanine nucleotide-exchange activity of Rabin8 toward Rab8 and these interactions play an important role in primary ciliogenesis[104]. We observed that PCA, ICA LLE, and RMSD analysis (using all residues) separated Rab11a structure co-crystallized with Rabin8 (PDB entry 4UJ5_B) from all other structures. The binding of Rabin8 to Rab11a is similar to that of P14KB. Rabin8 makes a few contacts in and near switch 1 (residues L38 and E39) and with loop K125-P135 in Rab11a. Vetter *et al*. have shown that Rab11a can simultaneously bind Rabin8 and FIP3, and Rabin8 has four times higher affinity for Rab11-FIP3 complex than for Rab11 alone [99]. We observed that Rabin8 induces conformational changes in both the switch regions of Rab11a (S53 Fig) and residue S40 adopts a position as observed in Rab11-FIP complexes.

PKGII is a serine/threonine kinase which has an important role in the regulation of bone growth, circadian rhythm, intestinal secretion, and renin secretion [15].PCA separated Rab11b co-crystallized with PKGII (PDB entry 4OJK_A) from other structures. Unlike the Rab11 structures interacting with effectors which are mentioned above, this Rab11 structure is GDP-bound and adopts inactive conformation. Yuasa *et al*. have shown that PKGII interacts specifically with GDP-bound Rab11b [105]. Reger *et al*. have shown that the interaction of PKGII with Rab11b is quite different from that observed in other complexes [15]. PKG II interacts with switch 1, switch 2, interswitch and N-terminal regions (D9-F12) of Rab11b. The interswitch, and N-terminal regions contribute to 55% of the total interaction interface [15]. The interactions with switch 2 contribute little to the overall interaction interface. They observed that even though Rab11b uses different interaction surfaces for recognition of PKGII and FIPs, these effectors cannot bind to Rab11b simultaneously due to steric clashes. They have also shown that interaction of PKGII with Rab11b is associated with major structural rearrangements in the switch 2 region and suggest that these changes are induced by the interactions of Y73 and I76 of Rab11 with that of PKGII. In the DCCM analysis, we have observed that switch 2 has correlated motions with the interswitch and the N-terminal region (D9-F12) of Rab11, with which PKGII interacts. We infer that these interactions at the interswitch and the N-terminal region could have an impact on the switch 2 region, which may have added to the structural perturbations at the switch 2 region.

Often, protein-protein interactions have been challenging yet attractive targets for drug design. Knowledge about protein-protein interactions may be helpful in designing drugs that could target protein-complexes involved in diseases [106]. Protein surfaces of protein-protein interactions may present transient binding sites (pockets) as reported for the proteins BCL-XL, IL-2, and MDM2[107].Additionally, the residues involved in protein-protein interactions may also serve as binding sites for drugs that could regulate the protein [106][108]. In this study, we focus on the structural elements of Rab11 protein, some of them are involved in the interactions of Rab11 with its binding partners. We have seen that there are unique conformational changes in Rab11 upon interacting with distinct partners and we identified a Rab11 representative structure for each such interaction. These representative structures can be used as starting points in computational studies that are aimed at specifically regulating Rab11 activity. For future studies, we envision virtual screening against aberrant protein-protein interactions in the complexes that are associated with diseases. The nature of interactions, either transient or obligate [109], needs to be accounted for the structural and functional properties of the involved proteins [110][111].

Majority of the FDA-approved drugs that are available in the market today, are designed to target protein's native ligand binding site (active site), some of which are highly conserved across protein family members. Targeting these active sites has the drawbacks such as unwanted side-effects, lack of efficacy and poor selectivity [112]. One solution to this problem is to design drugs that target allosteric sites in proteins [113]. Allosteric drugs work by stabilizing a unique conformation of protein and either activating or inhibiting the reactions carried out at the active site [113][114]. Moreover, some allosteric drugs exert their effects only when the natural ligand is bound to the target protein, thus preserving the spatial and temporal activity of the natural ligand [112]. Despite having clear benefits, only few allosteric drugs have entered the market, such as maraviroc[115], cinacalcet[116], benzodiazepines[112], etc. This is because the development of allosteric drugs is hindered by the lack of sufficient structural information of the protein. In this study, we have thoroughly analyzed the Rab11 representative structures and identified that there are many druggable pockets in the protein other than the nucleotide binding site. These binding sites present the opportunity to design allosteric drugs that regulate the protein.

Most of the drugs that are marketed by the pharmaceutical industry today are small molecules. Since these molecules have the benefits of oral bio-availability, efficiency and shortened half-life activity [117], they are often preferred over large molecules for the treatment of diseases. Previous studies have shown that small GTPases, particularly Rab, Rho and Ras from the Ras superfamily, can be regulated by small molecules that interferes with the protein functions in numerous ways such as interfering with the interaction between small-GTPases and GEF [118][119], inhibiting through covalent modification [120], interfering with the interaction between GTPase and effector[35][121][122], blocking membrane recruitment by inhibiting protein prenylation[123][124], etc. However, designing competitive small molecule inhibitors that binds to the nucleotide binding site of small GTPases is challenging since these proteins have high affinity for the nucleotide [125]. Even though few studies have reported such competitive inhibitors for small GTPases[34][126],these inhibitors have not been clinically successful. To overcome this challenge, it is highly desirable to design drugs that can bind to allosteric sites in these proteins with high affinity and selectivity. Previously we have identified three allosteric pockets in Ras protein and small molecules that binds to these sites [39]. In another study, we identified druggable non-nucleotide binding sites in Rab1 protein [37]. In this study, we employed structure-based virtual screening and identified many small molecules that have the ability to bind to two non-nucleotide binding sites (site 1 and site 2) in Rab11 with good binding affinities. We observed that site 1 and site 2 are flexible and undergoes conformational changes in Rab11. We have employed multiple Rab11 representative structures for virtual screening to account for the structural flexibility of Rab11 binding sites. During virtual screening, we observed that small molecules/ligands preferentially bind to either one of these sites in each representative structure. By doing network analysis and DCCM analysis of Rab11, we identified that the residues lining site 1 and site 2 may allosterically communicate with the nucleotide binding site in Rab11.

We observed that site 1 and site 2 are structurally conserved as allosteric pockets p2 and p3 in Ras[39]. These sites in Rab11 are also structurally conserved in Rab1. Interestingly, while these sites are structurally conserved in Rab1, Rab11 and Ras, they are poorly conserved at the sequence level, giving the opportunity to selectively regulate these proteins. Comparison of the correlated motions in Rab11 and Ras suggest that there are similarities in their dynamics as well. These observations imply that allosteric pockets may be structurally conserved in related proteins.

Even though the binding sites are similar in Rab11 and Ras, we observed some differences in the binding of the ligands to Rab11 compared to that observed in our previous study of Ras[39]. In the previous study, we have employed ligands from NCIDS II and ZINC drugs-now subset in ensemble-based virtual screening against Ras structures. During virtual screening, p3 was the most frequently targeted site. The top scoring ligands selected as promising leads in our study were also shown to target p3. Interestingly, in this study, we observed that the top scoring ligands target both site 1 and site 2 (Table 1). Additionally, we identified ligands that can specifically target each of these sites in Rab11. For example ZINC13099051 is scored well only in site 1 and ZINC04773602 is scored well only in site 2 of Rab11 structures. We also identified some ligands that specifically bind to different conformations of Rab11a and Rab11b. The ligands that specifically binds to GDP-bound conformations of Rab11a and Rab11b prefer site 1 (Tables 2 and 4) and those that specifically binds to GNP-bound conformations of Rab11a prefer site 2 (Table 3).

Rab proteins need to associate with membranes for their functional activity, which is accomplished by post translational modification of a Cysteine motif at the C-terminus by geranyl geranyl groups [127]. Membrane binding may alter the structural dynamics of Rab proteins, as it has been shown for H-Ras that its membrane binding is modulated by the nucleotide at the active site, and there are conformational changes in the protein associated with membrane binding [128]. In fact, it was shown that there is allosteric communication between the membrane interacting region and the nucleotide-binding region, and it has been suggested that the concerted motions between different regions in the protein upon transition between active and inactive conformations may have contributed to this [128]. This is encouraging, as the members of the Ras superfamily share a similar catalytic domain [129], which indicates that some similar allosteric mechanism may exist in Rab11. Further molecular dynamics simulation studies may provide more details in support of this. It may be also useful if future studies are carried out to assess how membrane binding alters the binding site accessibility in Rab proteins, using molecular dynamics simulation based methods such as pMD-membrane [130].

We have shown that the helix V102-H112 that comprises many of the site 1 and site 2 residues has direct interactions with switch 2. The perturbations at switch 2 are also reflected in this helix. We have shown in RMSF analysis that the switch 2 of Rab11 is highly flexible and in RMSD analysis we identified that the switch 2 regions deviates most from each other compared to the other regions of Rab11. Since site 1 and site 2 residues have direct communications with switch 2, and they have unique conformations depending on switch 2 perturbations, we infer that these sites may allow in regulating switch 2. The switch 2 of Rab11 is also involved in interactions with many effector proteins as we have mentioned above. Therefore, targeting site 1/site 2 may also allow regulation of those Rab-effector interactions. We observed that some small molecules are scored well only in certain conformations of Rab11. These molecules can be optimized for specifically targeting and stabilizing such conformations [131], as motivated by the discovery of reserpine that selectively binds to the *death* conformation of MutS Homolog (MSH) protein, but not its *repair* conformation [132]. We also observed that many of the top scoring molecules adhere to the Lipinski's rule of five, hence they are potential candidates for orally active drugs. We infer that the small molecules identified in our study can be further optimized as (i) drug candidates to allosterically modulate Rab11 at the respective binding sites or (ii) chemical probes to study Rab11 [133].Moreover, the insights on interactions of these ligands with Rab11 binding sites are likely to be useful in generating pharmacophore templates and scaffolds for further ligand screening studies. Besides site 1 and site 2, we also identified several binding pockets in Rab11 formed by RabF and RabSF regions. Opportunities to specifically regulate Rab11 using these binding sites can be explored in future studies. These binding sites can also be used as inputs for virtual screening studies employing molecular docking of known compounds [134], or binding pocket similarity of other proteins [135][136], to identify new drug repurposing opportunities. Additionally, using protocols such as LIBSA [137][138], the binding preferences of the small molecules for the binding sites identified in our study can be analyzed in future studies.

## Methods

### Analysis of Rab11 crystallographic structures

In order to generate the structural ensemble for Rab11, we first searched UniProt for a high-resolution PDB entry with the highest number of residues resolved, followed by visual examination using PyMOL. After examining the UniProt entry P62491 (human Rab11a), we have selected the PDB entry 1OIX [94], having a resolution of 1.7 Å, as a query to perform BLAST using the recommended BLAST cutoff value of 250 bits. An ensemble of 64 structures was formed as of 7^th^ July 2017. All the structures were resolved through X-ray crystallography and sourced from *Homo sapiens*. There were 60 Rab11a structures and 4 Rab11b structures (UniProt entry Q15907) in the resultant ensemble.

We excluded 36 structures from subsequent analysis because they either lacked resolved residues in key nucleotide binding regions or references in PDB. However, in the initial analysis, we included PDB entry 2F9L_A that lacks coordinates of residues E39-K41, since the structure has the highest resolution (1.55 Å) among Rab11b structures. Since we observed that the residues E39-K41 are important in Rab11 and including 2F9L_A in further analyses could bias towards incorrect conclusions, we excluded it from the ensemble (see S1 File). We used an ensemble of clustering approaches comprising PCA, ICA and LLE for the identification of clustroid representatives from the resultant ensemble of 27 Rab11 structures. Previously, PCA and ICA were used for the identification of clustroid representatives of Rab1 proteins. We used the Bio3D [139] package for identifying the most structurally invariant region of Rab11 structures. The C_α_ atom coordinates of the Rab11 structures are superposed using the structurally invariant region as a reference frame [92] to create an input matrix for PCA, ICA and LLE. Each row in the matrix corresponds to each Rab11 structure in the ensemble and the columns represent their C_α_ atom coordinates. In PCA, we used the matrix to define a covariance matrix (that represents their degree of linear relationship) from which the PCs are derived [139]. We projected the Rab11 structures onto the first three PCs, for which the maximum variance is observed [140]. Next, we performed ICA on the matrix and derived two independent components. Maximization of independence between components is done by maximizing non-Gaussianity[45]. We employed an ICA algorithm known as fastICA[45], where approximations to negentropy J are used for computing non-Gaussianity[45]. Finally, we employed LLE for neighborhood-preserving embeddings of the Rab11 structures from the high-dimensional space onto a low dimensional space. LLE is based on the principle that overlapping-local neighborhoods that are collectively analyzed, can provide information about global geometry [46]. LLE represents each sample in the data by a linear combination of its k nearest neighbors, with each neighbor weighted independently and it finally chooses the low dimensional representation that best preserves the weights in the target space [46]. Using LLE, we mapped the Rab11 structural data to a two-dimensional space. We chose the value of k based on an algorithm previously proposed by Kayo [141].

After mapping Rab11 structural data to low dimensional space using each of the above mentioned analyses, we performed hierarchical clustering and chosen the clustroids of the observed clusters as representative structures.

### Identification of binding sites

We used FTMap server available at http://ftmap.bu.edu to identify potential binding sites in Rab11 representative structures [56]. FTMap identifies binding hotspots on the protein surface by distributing small organic probe molecules (acetamide, acetonitrile, acetone, acetaldehyde, methylamine, benzaldehyde, benzene, isobutanol, cyclohexane, urea, dimethyl ether, ethanol, ethane, phenol, isopropanol, and N,N-dimethylformamide) of varying size, shape and polarity on the protein surface. Binding hotspots on the protein surface are identified by consensus sites that bind several different probe clusters [142]. For scoring the probe positions, FTMap employs an energy expression including electrostatic interaction energy based on Poisson-Boltzmann potential, pairwise interaction potential, van der Waals terms, and a cavity term based on hydrophobic contributions of the cavity [56].

### Virtual screening

We used the Rab11 representative structures for screening theNCI diversity subset III containing 1597 molecules. The NCI diversity subset III consists of compounds that are potential anti-cancer and anti-HIV agents. To account for structural and chemical diversity, the compounds in this subset were selected from 140,000 compounds by NCI Developmental Therapeutics program. We retrieved the subset was from the ZINC database (http://zinc.docking.org/). Virtual screening was performed using AutoDockVina utility [59] in the PyRx-0.8 [143]. Target structures were prepared by removing water molecules and adding polar hydrogen atoms using AutoDockTools[144]. Ligand structures were preparedby assigning partial chargesand were converted to PDBQT format using AutoDock utility in PyRx. Grid size dimensions for each target structure were set to cover the entire RabSF3 region. Default parameters were used for running AutoDockVina.

Using PyRx, we sorted the docked compounds based on their binding free energy predicted by AutoDockVina. Then the interactions of the top scored compounds for each target structure was visually inspected using PLIP [60] (https://projects.biotec.tu-dresden.de/plip-web/plip/). We computed weighted scores for each compound by giving 80% weight to binding free energy predicted by Vina and 20% weight to the number of interactions of the compounds with their respective sites. Based on the weighted scores, we identified the consensus top scorers (Table 1).

We employedsmina[62]and Vinardo scoring function[61]for redocking the top scored ligands on the respective sites in the Rab11 representative structures, to which they were docked during initial docking. We used the same input PDBQT files used for the initial docking as inputs for redocking. Default parameters were used for smina except for the scoring function.

We employed the program 'kcombu'[145]for post screening analysis of ligands, in order to find out whether there are any common substructures among the ligands. LigPlot+ [66][67]was used for plotting the interactions between the top scoring ligands and the Rab11 binding sites (Figs 4–9 and S31–S38 Figs). Since LigPlot+ and PLIP use different programs for mapping interactions, few differences between the interactions identified by LigPlot+ and PLIP are expected.

### Comparison of Rab11 binding sites with those of Rab1 and H-Ras

We compared Rab11 with Rab1 and H-Ras at their full sequence level and for site 1 and site 2. The amino acid sequences of Rab1a, Rab1b, Rab11a and Rab11b and H-Ras were retrieved from UniProt database [146]. We used Clustal Omega (https://www.ebi.ac.uk/Tools/msa/clustalo/) for sequence alignment of the amino acid sequences and computing sequence identity.

For comparison of site 1 and site 2, we first identified the point of contacts made be the top scoring ligands at site 1 and site 2 of Rab11, using PLIP [60]. ASP19, SER20, ARG74, THR77, LEU97, ASN101, ARG104, LYS107, GLU108, ASP111, HIS112 were identified as the residue contacts in site 1 and GLU103, LEU106, LEU109, ARG110, ALA113, SER115, ASN116, ILE117, VAL118, ILE119, ASN146, ASN147, LEU148, SER149, GLU171, ILE175 were identified as the residue contacts in site 2. Then, we extracted a sub selection of the full sequence alignment based on these residue contacts and computed their sequence identity using Clustal Omega.

### Network analysis of Rab11

We used NAPS [74], an interactive platform for network visualization and analysis (http://bioinf.iiit.ac.in/NAPS/), for creating RINs for Rab11 representatives. In the residue interaction networks, C_α_ atom of an amino acid residue is considered as a node and an edge is drawn if the C_α_–C_α_ distance between a pair of residues is within the threshold distance of 7 Å. Each edge between a pair of residues is assigned a weight based on the Euclidian distance between the C_α_ atoms of the residues [74].

Using NAPS we analyzed the betweenness centrality of Rab11 residues. Betweenness centrality of a node is the ratio of all shortest paths passing through a node to the total number of shortest paths in the network. Centrality values of nodes in a RIN can provide the topological importance of the node in the network. We also computed the shortest paths from the point of contacts made by the top scoring ligands in the newly identified sites to the active site employing NAPS. For identifying the residue contacts made by the top scoring ligands and the nucleotide, we employed PLIP [60]. The shortest path between a pair of nodes is chosen as the one with the minimum distance. Residues in the shortest paths have been experimentally shown to be important in allosteric communication [82][84]. We then employed CALCOM software [147] for measuring distance of Rab11 residues to the protein center of mass.

## Supporting information

S1 Fig Results of PCA on the ensemble of 27 Rab11 structures. (TIF)

S2 Fig Principal component analysis of Rab11 structures. Projection of Rab11 structures onto the first and second principal components (PCs) are shown in (A) and projection of Rab11 structures onto the first and third PCs are shown in (B).
(TIF)

S3 Fig Projection of Rab11 structures onto the subspace defined by first and second independent components. (TIF)

S4 Fig Projection of Rab11 structures onto a two-dimensional space using the locally linear embedding algorithm. (TIF)

S5 Fig Sequence alignment of Rab11a and Rab11b. RabF and RabSF motifs are colored in magenta and orange, respectively.
(TIF)

S6 Fig RMSD dendrogram of Rab11 structures. (TIF)

S7 Fig RMSD dendrogram of switch 1 (residues E39-V46) in Rab11 structures. (TIF)

S8 Fig RMSD dendrogram of interswitch (residues E47-T67) region in Rab11 structures. (TIF)

S9 Fig RMSD dendrogram of switch 2 (residues A68-A79) in Rab11 structures. (TIF)

S10 Fig Residues in and around switch 1 (residues D34-I41 in Rab1a) regions of Rab1 and Rab11. The residues of PDB entries 4FMB (B, D and F chains; Rab1a in complex with VirA), 4FMC (B and D chains; Rab1a in complex with ESPG), 4FMD (B and D chains; Rab1a in complex with ESPG) and 4FME (B and E chains; Rab1a in complex with ESPG) are colored in magenta, the residues of PDB entries 4IRU (B, D and F chains; Rab1a in complex with LepB) and 4JVS (B chain; Rab1a in complex with LepB) are colored in red, the residues of PDB entries 4HLQ (B, D, F, H and J chains; Rab1b in complex with TBC1D20) and 4I10 (A,C, E and G chains; Rab1b in complex with LepB) are colored in orange and the residues of PDB entry 4C4P (A chain; Rab11a in complex with FIP2) is colored in green. VirA and ESPG induce similar conformational changes in switch 1 of 4FMB, 4FMC, 4FMD and 4FME (Rab1a), LepB induces similar changes in switch 1 of 4IRU and 4JVS (Rab1a), TBC1D20 and LepB induce similar changes in switch 1 of 4HLQ and 4I1O (Rab1b).
(TIF)

S11 Fig Residue S40 in PDB entry 2D7C_A (Rab11 in complex with FIP2; magenta) and residue Y40 in PDB entries 3TKL_A (Rab1a-GTP in complex with LidA; red) and 3SFV_A(Rab1a-GDP in complex with LidA; green) are labeled. (TIF)

S12 Fig Binding sites identified by FTMap in Rab11 representative structures. Cartoon representations of Rab11 representative structures (A) 1OIV_A, (B) 1YZK_A, (C) 4C4P_A, (D) 4LX0_C and their rotated views are shown in the first and second rows, respectively. Cartoon representations of Rab11 representative structures (E) 4OJK_A, (F) 4UJ5_B, (G) 5C46_F, (H) 5JCZ_D and their rotated views are shown in the third and fourth rows, respectively. In the cartoon representations, probes occupying identified binding sites are shown in surface and colored yellow, blue, and red for C, N, and O atoms, respectively. Switches 1 and 2 are colored in cyan and pink, respectively. P loop is shown in green.
(TIF)

S13 Fig Binding sites in PDB entries 1OIV_A and 1YZK_A. The figure shows the binding site formed by residues Y99, F142, V102 and E103 (colored in orange) in PDB entries 1OIV_A ((A) and (C)) and 1YZK_A ((B) and (D)). The top panel shows the transparent surface of the PDB entries and the bottom panel shows the cartoon representation of the PDB entries, aligned in the same orientation.
(TIF)

S14 Fig Binding site formed by residues Y8-K13 and Y173-I175 in PDB entry 1YZK_A. In (A), transparent surface of 1YZK_A is shown and in (B), cartoon representation of 1YZK_A is shown. The binding site is colored in orange. The figures are aligned in the same orientation.
(TIF)

S15 Fig Binding site formed by residues Y10-I17 in PDB entries 4C4P_A, 4LX0_C and 5JCZ_D. The transparent surface of PDB entries 4C4P_A, 4LX0_C and 5JCZ_D are shown in (A), (B) and (E), respectively. The cartoon representations of 4C4P_A, 4LX0_C and 5JCZ_D are shown in (C), (D) and (F), respectively. The binding site is colored in cyan. The figures are aligned in the same orientation.
(TIF)

S16 Fig Binding site formed by residues D19, S20, G69, Q70, E71 and W105 in PDB entries 1YZK_A and 4C4P_A. The transparent surface of PDB entries 1YZK_A and 4C4P_A are shown in (A) and (B), respectively. The cartoon representations of 1YZK_A and 4C4P_A are shown in (C) and (D), respectively. The binding site is colored in orange. The figures are aligned in the same orientation.
(TIF)

S17 Fig Binding site formed by residues D19, S20, G69, Q70, E71 and W105 in PDB entries 4LX0_C and 4OJK_A. The transparent surface of PDB entries 4LX0_C and 4OJK_A are shown in (A) and (B), respectively. The cartoon representations of 4LX0_C and 4OJK_A are shown in (C) and (D), respectively. The binding site is colored in orange. The figures are aligned in the same orientation.
(TIF)

S18 Fig Binding site formed by residues D19, S20, G69, Q70, E71 and W105 in PDB entries 4UJ5_B, 5C46_F and 5JCZ_D. The transparent surface of PDB entries 4UJ5_B, 5C46_F and 5JCZ_D are shown over their secondary structures in (A), (B) and (E), respectively. The cartoon representations of 4UJ5_B, 5C46_F and 5JCZ_D are shown in (C), (D) and (F), respectively. The binding site is colored in orange. The figures are aligned in the same orientation.
(TIF)

S19 Fig Binding site formed by residues G45, T67, A68, R74 and I76 in PDB entries 1YZK_A and 4UJ5_B. The transparent surface of PDB entries 1YZK_A and 4UJ5_B are shown over their secondary structures in (A) and (B), respectively. The cartoon representations of 1YZK_A and 4UJ5_B are shown in (C) and (D), respectively. The binding site is colored in orange. The figures are aligned in the same orientation.
(TIF)

S20 Fig Binding site formed by residues S25-S42 and T43-E47 in PDB entry 4UJ5_B. In (A), transparent surface of 4UJ5_B is shown and in (B), cartoon representation of 4UJ5_B is shown. The binding site is colored in orange. The figures are aligned in the same orientation.
(TIF)

S21 Fig Binding sites in PDB entry 1OIV_A. In (A), transparent surface of 1OIV_A is shown and in (B), cartoon representation of 1OIV_A is shown. In (A) and (B), the binding site formed by residues I17, T67, R72, Y80, Y81, W105, E108 and the binding site formed by residues N101, R104, W105 and E108 are colored in cyan and orange, respectively.
(TIF)

S22 Fig Similar binding sites in PDB entries 2WWX_A (Rab1) and 4C4P_A (Rab11). Binding sites formed by residues 10–17 in Rab1 and Rab11 structures are shown. RabSF1 (residues 8–13) and Switch 2 (Residues 68–79) are colored in orange and pink, respectively. Probes occupying identified binding sites are shown in surface and colored yellow, blue, and red for C, N, and O atoms, respectively.
(TIF)

S23 Fig Binding site formed by residues K10-S17 in PDB entries 2FOL_A and 2WWX_A. The transparent surface of PDB entries 2FOL_A and 2WWX_A are shown in (A) and (B), respectively. The cartoon representations of 2FOL_A and 2WWX_A are shown in (C) and (D), respectively. The binding site is colored in orange. The figures are aligned in the same orientation.
(TIF)

S24 Fig Binding site formed by residues K10-S17 in PDB entry 4I1O_E (Rab1) and the similar binding site in PDB entry 4C4P_A (Rab11). In (A), transparent surface of PDB entry 4I1O_E is shown and in (B), cartoon representation of the PDB entry 4I1O is shown. The binding site formed by residues K10-S17is colored in orange in (A) and (B). In (C), the similar binding site in PDB entry 4C4P_A formed by residues Y10-I17, is colored in cyan on its cartoon representation. The figures are aligned in the same orientation. The binding site in 4C4P_A is shown in its 3D structure in S15 Fig.
(TIF)

S25 Fig Similar binding pockets in PDB entries 3TKL_A (Rab1) and 1YZK_A (Rab11). Pockets formed by residues 99, 102, 103 and 142 in Rab1 and Rab11 are colored in blue. Probes occupying identified binding sites are shown in sticks and colored yellow, blue, and red for C, N, and O atoms, respectively.
(TIF)

S26 Fig Binding site formed by residues 99, 102, 103 and 142 in PDB entries 3TKL_A and 4FMB_D. The transparent surface of PDB entries 3TKL_A and 4FMB_D are shown in (A) and (B), respectively. The cartoon representations of 3TKL_A and 4FMB_D are shown in (C) and (D), respectively. The binding site is colored in yellow. The figures are aligned in the same orientation.
(TIF)

S27 Fig Binding site formed by residues 99, 102, 103 and 142 in PDB entry 4I1O_E (Rab1) and the similar binding site in PDB entry 1OIV_A (Rab11). In (A), transparent surface of PDB entry 4I1O_E is shown and in (B), cartoon representation of the PDB entry is shown. The binding site formed by residues V99, W102, L103 and S142 in 4I1O_E is colored in yellow in (A) and (B). In (C), the similar binding site in PDB entry 1OIV_A formed by residues Y99, V102, E103 and F142, is colored in orange on its cartoon representation. The figures are aligned in the same orientation. The binding site is shown in the 3D representation of 1OIV_A in S13 Fig.
(TIF)

S28 Fig Binding site formed by residues N101, Q104, W105 and E108 in Rab1 and the similar binding site in Rab11. The transparent surface of PDB entries 3SFV_A (Rab1) and 3TKL_A (Rab1) are shown over their secondary structures in (A) and (B), respectively. The cartoon representations of 3SFV_A and 3TKL_A are shown in (C) and (D), respectively. The binding site formed by residues N101, Q104, W105 and E108 is colored in yellow. In (E), the similar binding site formed by residues N101, R104, W105 and E108 in PDB entry 1OIV_A (Rab11) is colored in orange on its cartoon representation. The figures are aligned in the same orientation. The binding site is shown in the 3D representation of 1OIV_A in S21 Fig.
(TIF)

S29 Fig Residues 8–13 in Rab1 and Rab11 that form binding sites. The transparent surface of PDB entries 2WWX_A (Rab1), 3SFV_A (Rab1) and, 4I1O_E (Rab1) are shown in (A), (B) and (C), respectively. The cartoon representations of 2WWX_A, 3SFV_A and 4I1O_E are shown in (D), (E) and (F), respectively. Residues 8–13 in Rab1 is colored in orange in (A)-(F). In (G), residues Y8-K13 in PDB entry 1YZK_A (Rab11) is colored in yellow on its cartoon representation. The figures are aligned in the same orientation. The binding site is shown in the 3D representation of 1YZK_A in S18 Fig.
(TIF)

S30 Fig Binding site in Rab1 which is not observed in Rab11. The binding site in Rab1 (PDB entry 2FOL_A) formed by residues L25-D31, which is not observed in any Rab11 representatives, is colored in yellow in its (A) transparent surface and (B) cartoon representation.
(TIF)

S31 Fig Ligands ZINC00084617 and ZINC13208966 that prefer GDP-bound Rab11a. The figure shows the interactions of the ligands (labeled) with the residues of PDB entry 1OIV_A. The ligand and Rab11 side chains are shown in ball-and-stick representation. Black circles denote carbon atoms, red circles denote oxygen atoms, and blue circles denote nitrogen atoms. The ligand bonds are colored in purple. Residues in Rab11 interacting with the ligand are labeled. Hydrogen bonds are shown as green dotted lines. The Rab11 residues making nonbonded contacts with the ligand are shown as spoked arcs. Figures are generated using LigPlot+ [66][67].
(TIF)

S32 Fig Ligands ZINC04720972 and ZINC11677172 that prefer GDP-bound Rab11a. The figure shows the interactions of the ligands (labeled) with the residues of PDB entry 1OIV_A. ZINC11677172 also has interactions with a sulfate ion present near its binding site in the target structure. The ligand and Rab11 side chains are shown in ball-and-stick representation. Black circles denote carbon atoms, red circles denote oxygen atoms, yellow circle denotes sulfur atom, green circle denotes fluorine atom and blue circles denote nitrogen atoms. The ligand bonds are colored in purple. Residues in Rab11 interacting with the ligand are labeled. Hydrogen bonds are shown as green dotted lines. The Rab11 residues making nonbonded contacts with the ligand are shown as spoked arcs. Figures are generated using LigPlot+ [66][67].
(TIF)

S33 Fig Ligands ZINC00393674 and ZINC01701460 that prefer GDP-bound Rab11a. The figure shows the interactions of the ligands (labeled) with the residues of PDB entry 1OIV_A. The ligand and Rab11 side chains are shown in ball-and-stick representation. Black circles denote carbon atoms, red circles denote oxygen atoms, green circles denote chlorine atoms and blue circles denote nitrogen atoms. The ligand bonds are colored in purple. Residues in Rab11 interacting with the ligand are labeled. Hydrogen bonds are shown as green dotted lines. The Rab11 residues making nonbonded contacts with the ligand are shown as spoked arcs. Figures are generated using LigPlot+ [66][67].
(TIF)

S34 Fig Ligands ZINC15952559 and ZINC11677178 that prefer GNP-bound Rab11a. The figure shows the interactions of the ligands (labeled) with the residues of the Rab11 structures in which they scored the best. The ligand and Rab11 side chains are shown in ball-and-stick representation. Black circles denote carbon atoms, red circles denote oxygen atoms, green circles denote fluorine atoms and blue circles denote nitrogen atoms. The ligand bonds are colored in purple. Residues in Rab11 interacting with the ligand are labeled. Hydrogen bonds are shown as green dotted lines. The Rab11 residues making nonbonded contacts with the ligand are shown as spoked arcs. Figures are generated using LigPlot+ [66][67].
(TIF)

S35 Fig Ligands ZINC12671898 and ZINC17353914 that prefer GNP-bound Rab11a. The figure shows the interactions of the ligands (labeled) with the residues of the Rab11 structures in which they scored the best (see Table 3). The ligand and Rab11 side chains are shown in ball-and-stick representation. Black circles denote carbon atoms, red circles denote oxygen atoms and blue circles denote nitrogen atoms. The ligand bonds are colored in purple. Residues in Rab11 interacting with the ligand are labeled. Hydrogen bonds are shown as green dotted lines. The Rab11 residues making nonbonded contacts with the ligand are shown as spoked arcs. Figures are generated using LigPlot+ [66][67].
(TIF)

S36 Fig Ligands ZINC01573829 and ZINC01577889 that prefer GNP-bound Rab11a. The figure shows the interactions of the ligands (labeled) with the residues of the Rab11 structures in which they scored the best (see Table 3). The ligand and Rab11 side chains are shown in ball-and-stick representation. Black circles denote carbon atoms, red circles denote oxygen atoms, yellow circle denote sulfur atom and blue circles denote nitrogen atoms. The ligand bonds are colored in purple. Residues in Rab11 interacting with the ligand are labeled. Hydrogen bonds are shown as green dotted lines. The Rab11 residues making nonbonded contacts with the ligand are shown as spoked arcs. Figures are generated using LigPlot+ [66][67].
(TIF)

S37 Fig Ligands ZINC29590275 and ZINC01726776 that prefer GNP-bound Rab11a. The figure shows the interactions of the ligands (labeled) with the residues of the Rab11 structures in which they scored the best. The ligand and Rab11 side chains are shown in ball-and-stick representation. Black circles denote carbon atoms, red circles denote oxygen atoms, yellow circle denote sulfur atom and blue circles denote nitrogen atoms. The ligand bonds are colored in purple. Residues in Rab11 interacting with the ligand are labeled. Hydrogen bonds are shown as green dotted lines. The Rab11 residues making nonbonded contacts with the ligand are shown as spoked arcs. Figures are generated using LigPlot+ [66][67].
(TIF)

S38 Fig Structures of diverse ligands that prefer GDP-bound Rab11b. The figure shows the interactions of the structurally diverse ligands (labeled) that prefer PDB entry 4OJK_A and their interactions. ZINC17465979, ZINC17465983, ZINC05462674 and ZINC05462670 are isomers. The ligand and Rab11 side chains are shown in ball-and-stick representation. Black circles denote carbon atoms, red circles denote oxygen atoms, green circles denote fluorine atoms and blue circles denote nitrogen atoms. The ligand bonds are colored in purple. Residues in Rab11 interacting with the ligand are labeled. Hydrogen bonds are shown as green dotted lines. The Rab11 residues making nonbonded contacts with the ligand are shown as spoked arcs. Figures are generated using LigPlot+ [66][67].
(TIF)

S39 Fig Conformational changes of site 1 in Rab11 representative structures. Surface representations of PDB entries 1OIV_A, 1YZK_A, 4C4P_A, 4LX0_C, 4OJK_A, 4UJ5_B, 5C46_F, and 5JCZ_D are shown in (A), (B), (C), (D), (E), (F), (G) and (H), respectively. Residues 19, 20, 101, 104, 105, 108 and 111 forming site 1 are colored in green.
(TIF)

S40 Fig Conformational changes of site 2 in Rab11 representative structures. Surface representations of PDB entries 1OIV_A, 1YZK_A, 4C4P_A, 4LX0_C, 4OJK_A, 4UJ5_B, 5C46_F and 5JCZ_D are shown in (A), (B), (C), (D), (E), (F), (G) and (H), respectively. Residues 106, 109, 110, 113, 117, 118, 119, 148, 149 and 171 forming site 2 are colored in blue.
(TIF)

S41 Fig **Ligands docked at PDB entries (A) 5C46_F and (B) 5JCZ_D.** Ligands (A) ZINC29590257 and (B) ZINC01568793 are shown in brown sticks. Residues of site 1 are colored in green and ligands are shown as brown sticks. In 5C46_F, ligands interact with a small cavity formed by residues H99, L100 and A142 which are colored in cyan.
(TIF)

S42 Fig Rab11 structures 1OIV_A (GDP-bound) and 4C4P_A (GNP-bound). The ligand and Rab11 side chains are shown in ball-and-stick representation. The ligand bonds are colored in purple. Hydrogen bonds are shown as green dotted lines. The Rab11 residues making nonbonded contacts with the ligand are shown as spoked arcs. The red circle and ellipses indicate protein residues that are in non-equivalent positions when the two structures are superposed. The figure shows that compared to 1OIV_A, there are two extra Rab11 residues (Thr43 from switch 1, and GLY69 from switch 2) that have hydrogen bonds with the ligand. The Figures are generated using LigPlot+ [66][67].
(TIF)

S43 Fig **Conformation of switch regions in (A) PDB entry 4UJ5_B and (B) PDB entry 5C46_F.** Switch 1 and switch 2 of the PDB entries are colored in cyan and magenta, respectively. The ligands at the active sites of the structures are colored in red. The magnesium ion at the active site of the structures is shown as green spheres. Water molecules are shown as red spheres. The site 1 and site 2 residues are colored in green and blue, respectively. Residues GLY69 (magenta; from switch 1 region), SER20 (green; from site 1) and ARG104 (green, from site 1) are labeled in both structures. GLY69 and SER20 have hydrogen bond interactions with the ligand at the active site. The switch 2 region and ARG104 adopt different conformations in them. Figures are generated using UCSF Chimera.
(TIF)

S44 Fig **Multiple sequence alignment of (A) site 1 and (B) site 2 in Rab1 and Rab11.** Residues conserved in three or more sequences are highlighted in green. Residues conserved only in Rab11 are highlighted in yellow. Residues conserved only in Rab1 are highlighted in cyan.
(TIF)

S45 Fig **Plots generated by NAPS server showing betweenness centrality values of residues in (A) 1OIV_A, (B) 1YZK_A, (C) 4C4P_A, (D) 4LX0_C, (E) 4OJK_A, (F) 4UJ5_B, (G) 5C46_F and (H) 5JCZ_D.** X axis shows residue number and Y axis shows betweenness centrality values of residues.
(TIF)

S46 Fig Site 1 is structurally conserved as allosteric pocket p2 in H-Ras. The figure shows Rab11 (PDB entry 1OIV_A) and H-Ras (PDB entry 2Q21_A) structures aligned in the same orientation. (A) and (B) show site 1 (colored in green) in cartoon representations of Rab11 and H-Ras. (C) and (D) show site 1 (colored in green) in surface representations of Rab11 and H-ras. Residues 19, 20, 101, 104, 105, 108 and 111 of PDB entry 1OIV_A are colored in green. Residues 61–65 and 90–95 of PDB entry 2Q21_A are colored in green.
(TIF)

S47 Fig Site 2 is structurally conserved as allosteric pocket p3 in H-Ras. The figure shows Rab11 (PDB entry 1OIV_A) and H-Ras (PDB entry 2Q21_A) structures aligned in the same orientation. (A) and (B) show site 2 (colored in blue) in cartoon representations of Rab11 and H-Ras. (C) and (D) show site 2 (colored in blue) in surface representations of Rab11 and H-Ras. Residues 110, 117–119, 148, 149 and 171 of PDB entry 1OIV_A are colored in blue. Residues 97, 101, 107–111, 136–140 and 161–166 of PDB entry 2Q21_A are colored in blue.
(TIF)

S48 Fig Pairwise sequence alignment of Rab11a and H-Ras sequences. Helix α2 (residues Y64-G77) in H-Ras and the corresponding amino acid sequences in the PSA are indicated in magenta color. Residues of helix α3 (I93-V103) and residues D105-V109 and residues S136-P140 that line pocket p3 in H-Ras and the corresponding residues in the PSA that line sites 1 and 2 in Rab11 are indicated in red.
(TIF)

S49 Fig Residues of site 1 and site 2 that are conserved in H-Ras allosteric sites. The figure shows the sequence alignment of site 1 and site 2 residues that are contacted by small molecules during virtual screening, and the corresponding residues in H-Ras pockets p2 and p3, respectively. The residues conserved between the binding sites are highlighted in green shade.
(TIF)

S50 Fig Switch and interswitch regions of Rab11a-FIP complexes (PDB entries 2GZD_A, 2GZD_B, 2GZH_A, 4C4P_A, 2D7C_A, 2D7C_B, 2HV8_A, 2HV8_B, 2HV8_C, 4UJ3_A and 4UJ3_G; cyan) and unbound-Rab11a-GTP (PDB entry 1OIW_A; yellow). Residue S40 that adopts different orientations in the structures is labeled. Switch 1 region of these Rab11a-FIP complexes are similar and switch 2 adopts different conformations.
(TIF)

S51 Fig Switch regions of Rab11a-Myosin 5a complex (PDB entries 5JCZ_A; green), Rab11a-Myosin 5b complex (PDB entries 4LX0_A; orange) and unbound-Rab11a-GTP (1OIW_A; yellow). Residue S40 adopts different orientations in the structures. Switch 1 and switch 2 adopt different conformations from those of unbound-Rab11a.
(TIF)

S52 Fig Switch regions of Rab11a-P14KB complex (PDB entry 4DOL_B; red), and unbound-Rab11a-GTP (1OIW_A; yellow). P14KB induces conformational changes in the switch regions of Rab11a. Unlike the observations on residue S40 in other protein complexes used in our study, residue S40 adopts a conformation similar to that in unbound-Rab11a-GTP.
(TIF)

S53 Fig Switch regions of Rab11a-Rabin8 complex (PDB entry 4UJ5_A; magenta), and unbound-Rab11a-GTP (1OIW_A; yellow). Rabin8 induces conformational changes in the switch regions of Rab11a.
(TIF)

S1 Table Rab11 structures in the ensemble of 27 structures. For each Rab11 structure in the ensemble, the experimental technique used for determining the structure, its resolution and its ligands are listed. GSP stands for 5'-Guanosine-Diphosphate-Monothiophosphate, MG stands for Magnesium ion, GNP stands for Phosphoaminophosphonic acid-guanylate ester, SO4 stands for sulfate ion, 2ME stands for Methoxyethane, MES stands for 2-(N-morpholino)-ethanesulfonic acid, GTP stands for Guanosine-5'-Triphosphate, GDP stands for Guanosine-5'-Diphosphate, CL stands for Chloride ion, PO4 stands for phosphate ion, GOL stands for glycerol, BEF stands for Beryllium trifluoride ion, EDO stands for Ethylene glycol, ACT stands for Acetate ion, MSE stands for Selenomethionine and NI stands for Nickel (ii) ion.
(DOCX)

S2 Table Rab11 structures, their interacting partners and binding sites. Residues in Rab11 that are not conserved in Rab1 are highlighted in cyan.
(DOCX)

S3 Table Rab1 structures, their interacting partners and binding sites. Residues in Rab1 that are not conserved in Rab11 are highlighted in cyan.
(DOCX)

S4 Table Binding sites identified by FTMap in Rab11 representative structures. Residues of switches 1 and 2 (residues 39–46 and 68–79) are shown in bold. RabF and RabSF regions are highlighted in magenta and orange, respectively.
(DOCX)

S5 Table Free energy values computed by Vinardo. The table lists the free energy values computed by Vinardo for the top scoring ligands when docked at different sites in Rab11 representative structures.
(DOCX)

S6 Table Vinardo scores for ligands targeting GDP-bound Rab11a (PDB entry 1OIV_A). The target sites of ligands in 1OIV_A and their free energy of binding computed by Vinardo are listed. GDP stands for Guanosine-5'-Diphosphate.
(DOCX)

S7 Table Vinardo scores for ligands that target GNP-bound conformations of Rab11a. The target sites of ligands, and their free energy of binding computed by Vinardo are listed. GNP stands for Phosphoaminophosphonic acid-guanylate ester.
(DOCX)

S8 Table Vinardo scores for ligands that target GDP-bound Rab11b (PDB entry 4OJK_A). The target sites of ligands and their free energy of binding computed by Vinardo are listed. GDP stands for Guanosine-5'-Diphosphate.
(DOCX)

S9 Table Sequence identities of Rab sequences. Sequence identities of Rab1a, Rab1b, Rab11a and Rab11b as computed by Clustal Omega are shown above.
(DOCX)

S10 Table Shortest paths from residues in site 1 and site 2 to the nucleotide-binding site in Rab11. The table above lists the shortest paths from site 1 and site 2 to the nucleotide binding site, that are observed to have minimal length during network analysis. The second column of the table shows the PDB entries for which such shortest paths are observed and the third column lists the shortest paths.
(DOCX)

S11 Table Residues in site 1 and site 2 of Rab11 representative structures that have high betweenness centrality and their values, as computed by NAPS server. (DOCX)

S1 File Details on Rab11, Ras, Rab1 proteins and the initial analyses performed on Rab11 proteins. (PDF)

S2 File Sequence alignments of Rab1 and Rab11 sequences. (PDF)

S3 File Figures showing interactions of various ligands with Rab11. (PDF)
