# Supplementary figures and images for: Allosteric binding sites in Rab11 for potential drug candidates
Source: PLoS One. 2018 Jun 6;13(6):e0198632. doi: 10.1371/journal.pone.0198632 (PMC5991966; doi:10.1371/journal.pone.0198632)

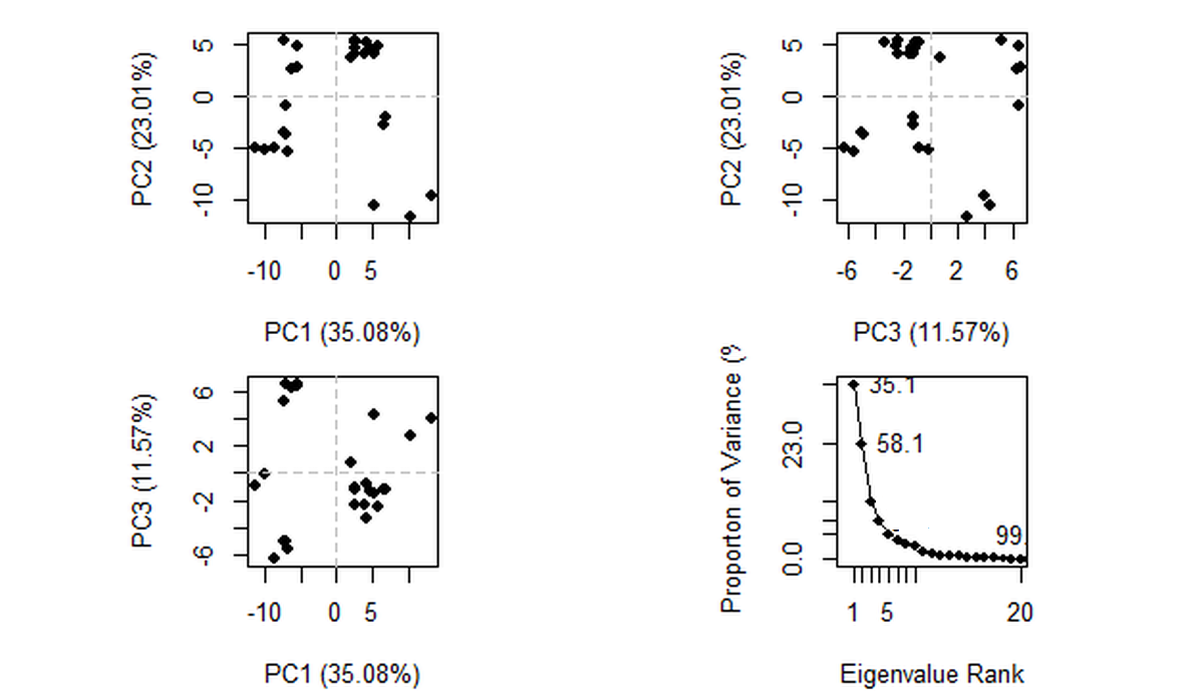

Supplement: S1 Fig — (TIF) [file pone.0198632.s001.tif]

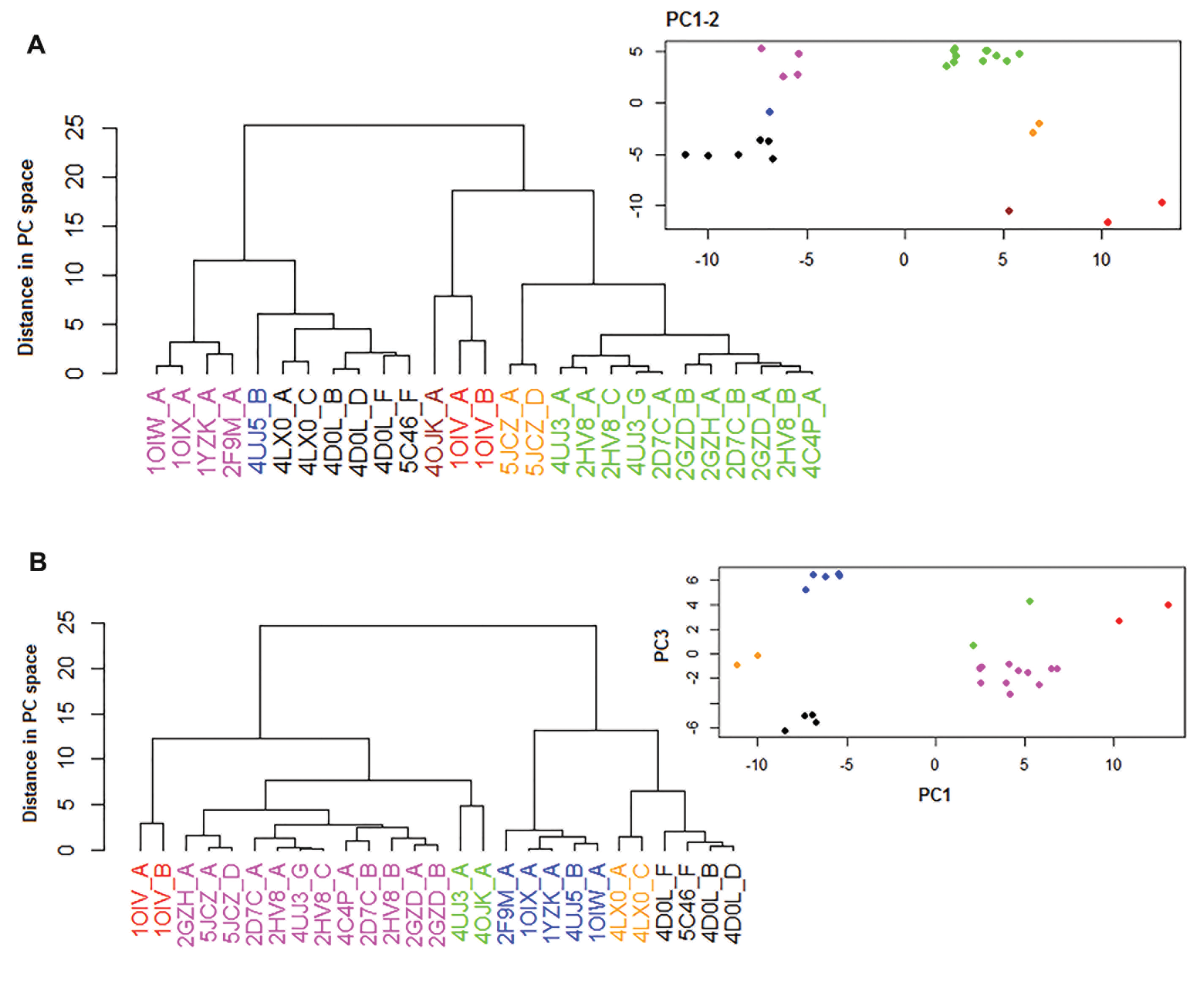

Supplement: S2 Fig — Projection of Rab11 structures onto the first and second principal components (PCs) are shown in (A) and projection of Rab11 structures onto the first and third PCs are shown in (B). (TIF) [file pone.0198632.s002.tif]

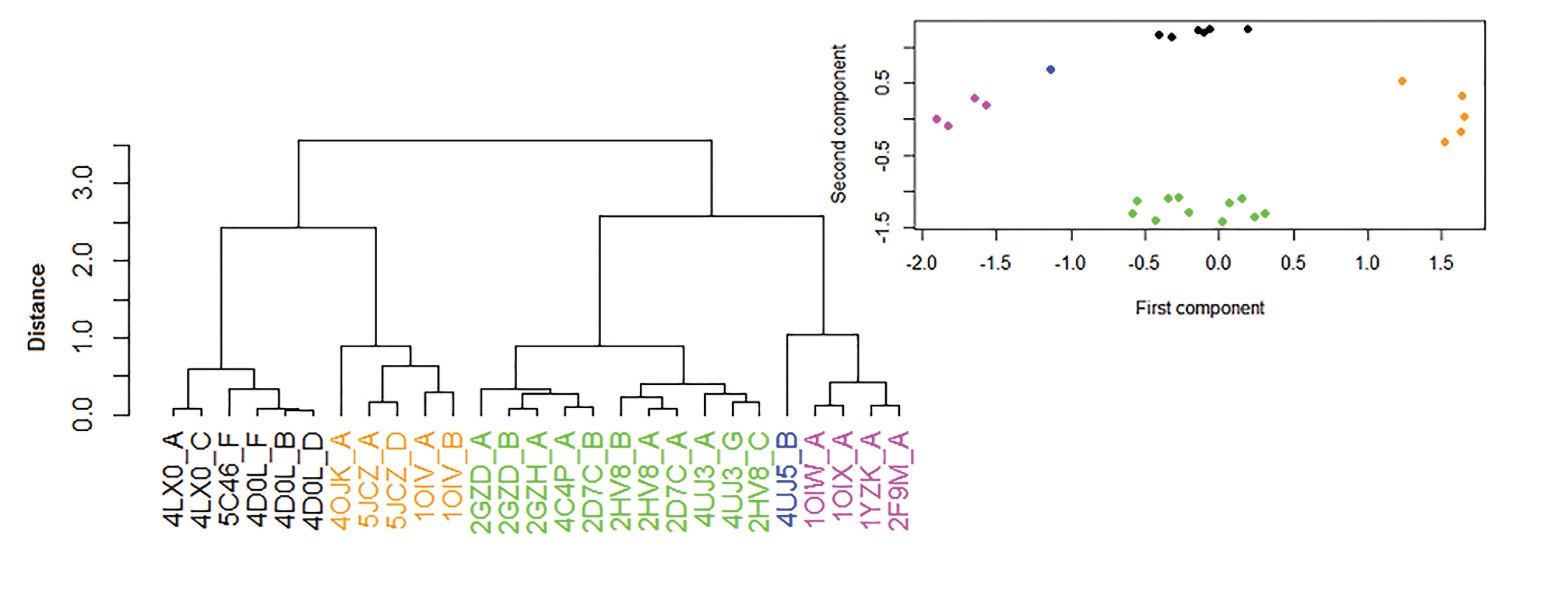

Supplement: S3 Fig — (TIF) [file pone.0198632.s003.tif]

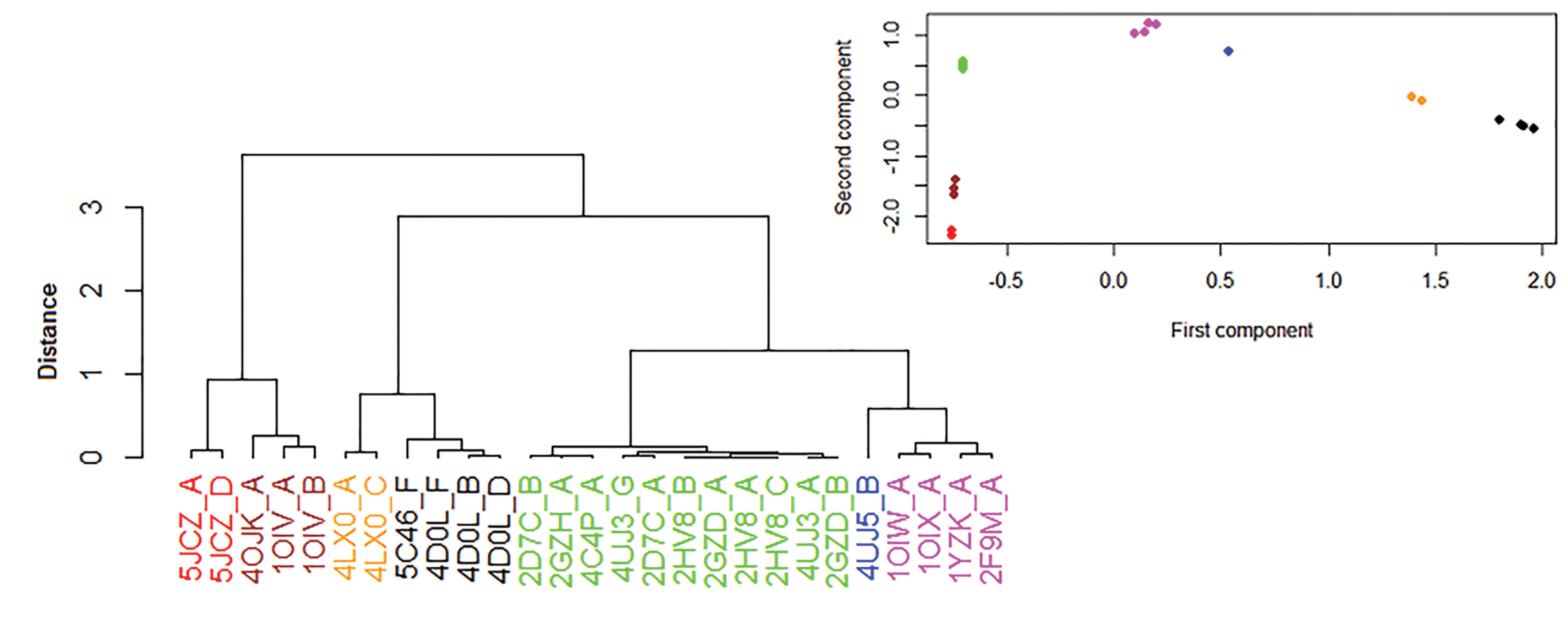

Supplement: S4 Fig — (TIF) [file pone.0198632.s004.tif]

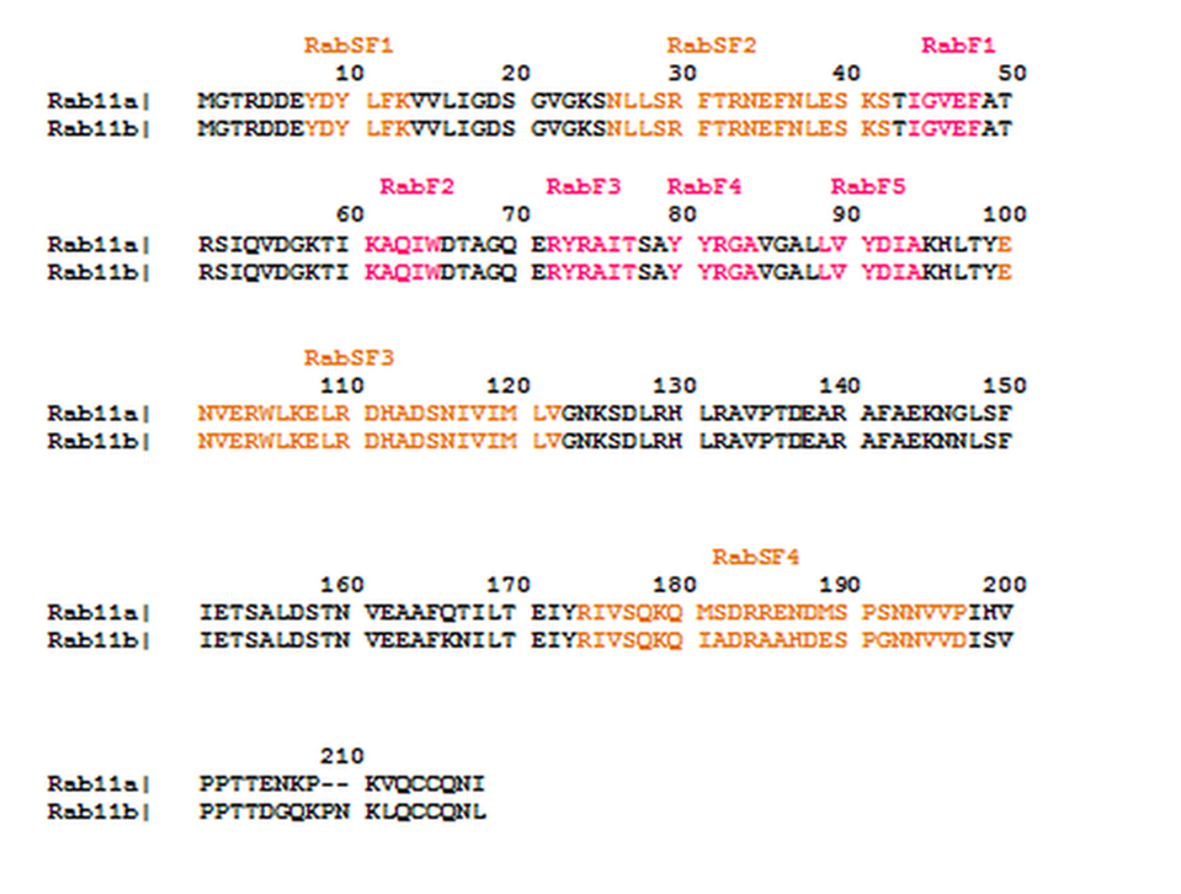

Supplement: S5 Fig — RabF and RabSF motifs are colored in magenta and orange, respectively. (TIF) [file pone.0198632.s005.tif]

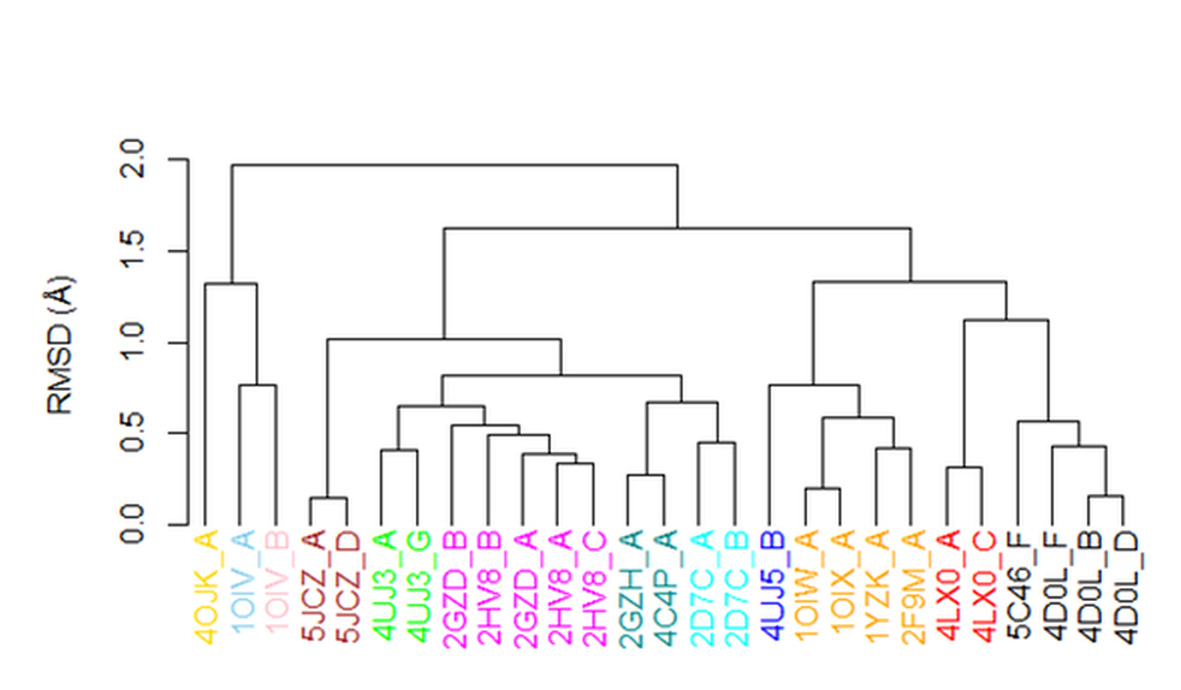

Supplement: S6 Fig — (TIF) [file pone.0198632.s006.tif]

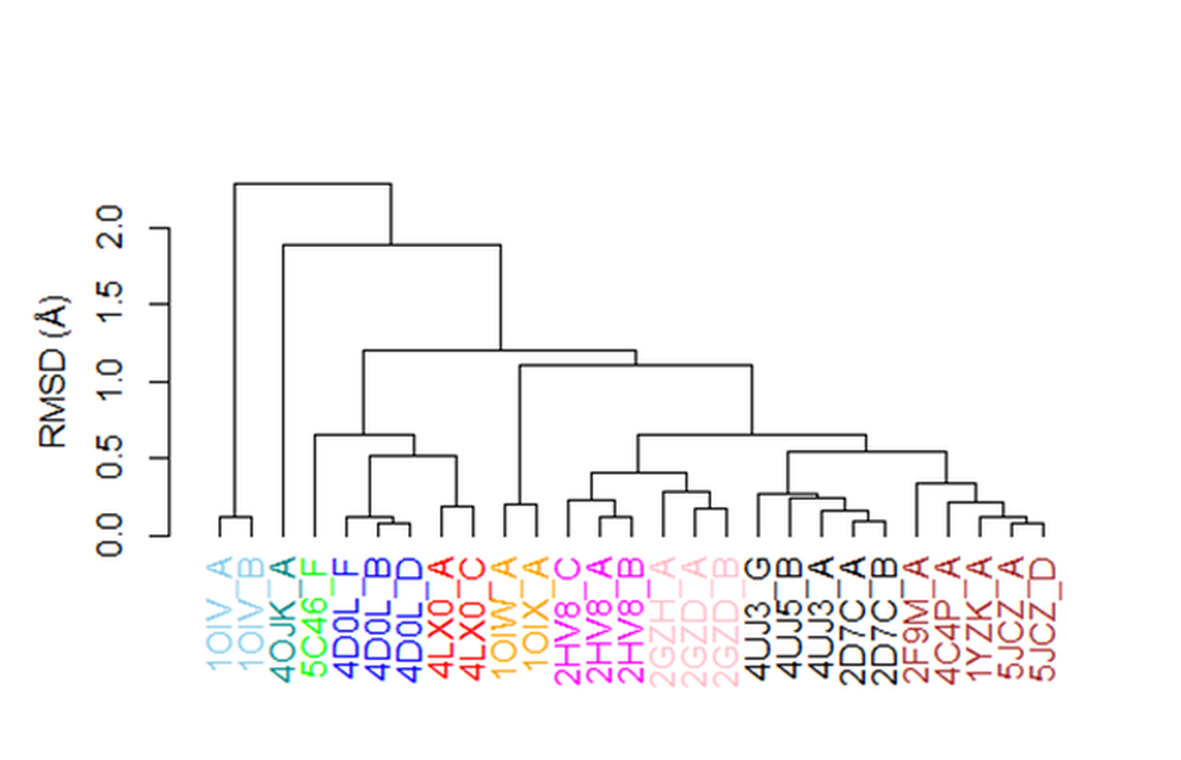

Supplement: S7 Fig — (TIF) [file pone.0198632.s007.tif]

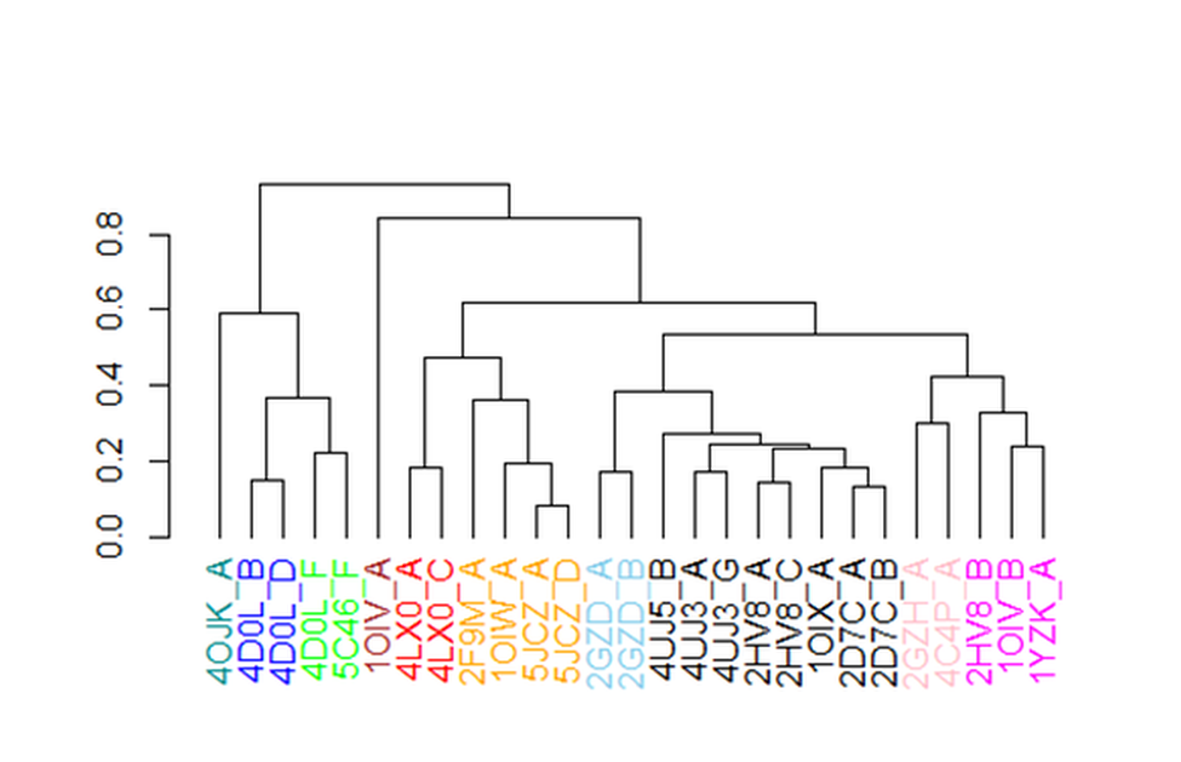

Supplement: S8 Fig — (TIF) [file pone.0198632.s008.tif]

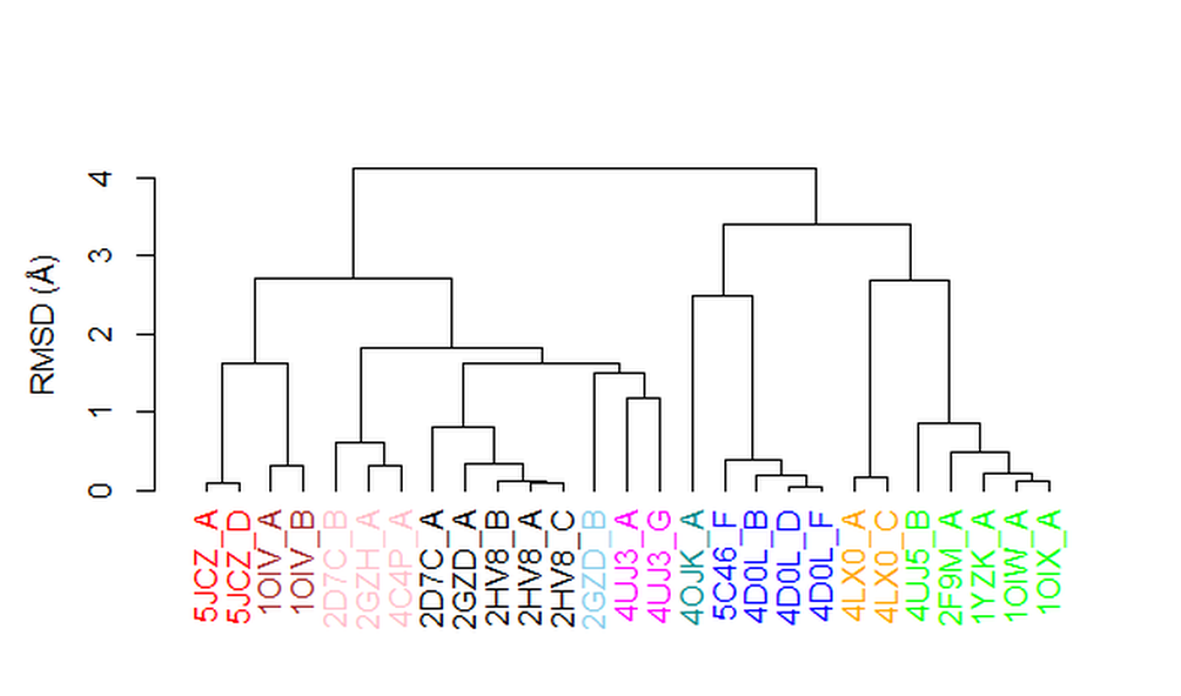

Supplement: S9 Fig — (TIF) [file pone.0198632.s009.tif]

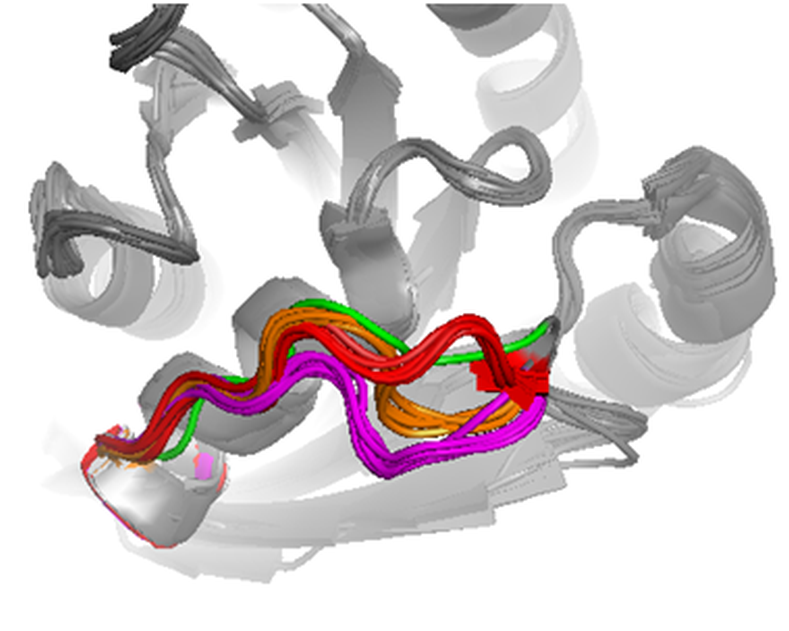

Supplement: S10 Fig — The residues of PDB entries 4FMB (B, D and F chains; Rab1a in complex with VirA), 4FMC (B and D chains; Rab1a in complex with ESPG), 4FMD (B and D chains; Rab1a in complex with ESPG) and 4FME (B and E chains; Rab1a in complex with ESPG) are colored in magenta, the residues of PDB entries 4IRU (B, D and F chains; Rab1a in complex with LepB) and 4JVS (B chain; Rab1a in complex with LepB) are colored in red, the residues of PDB entries 4HLQ (B, D, F, H and J chains; Rab1b in complex with TBC1D20) and 4I10 (A,C, E and G chains; Rab1b in complex with LepB) are colored in orange and the residues of PDB entry 4C4P (A chain; Rab11a in complex with FIP2) is colored in green. VirA and ESPG induce similar conformational changes in switch 1 of 4FMB, 4FMC, 4FMD and 4FME (Rab1a), LepB induces similar changes in switch 1 of 4IRU and 4JVS (Rab1a), TBC1D20 and LepB induce similar changes in switch 1 of 4HLQ and 4I1O (Rab1b). (TIF) [file pone.0198632.s010.tif]

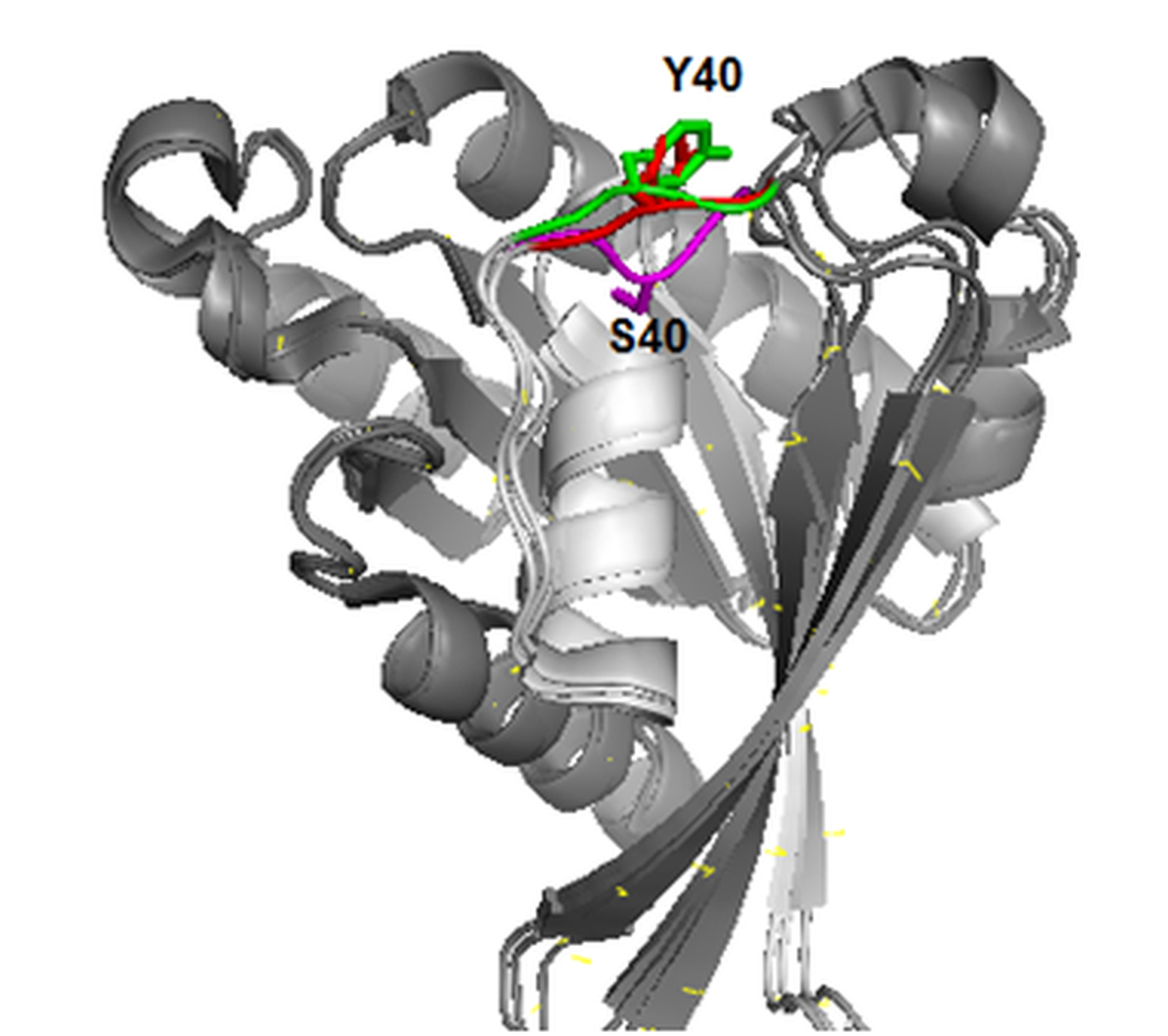

Supplement: S11 Fig — (TIF) [file pone.0198632.s011.tif]

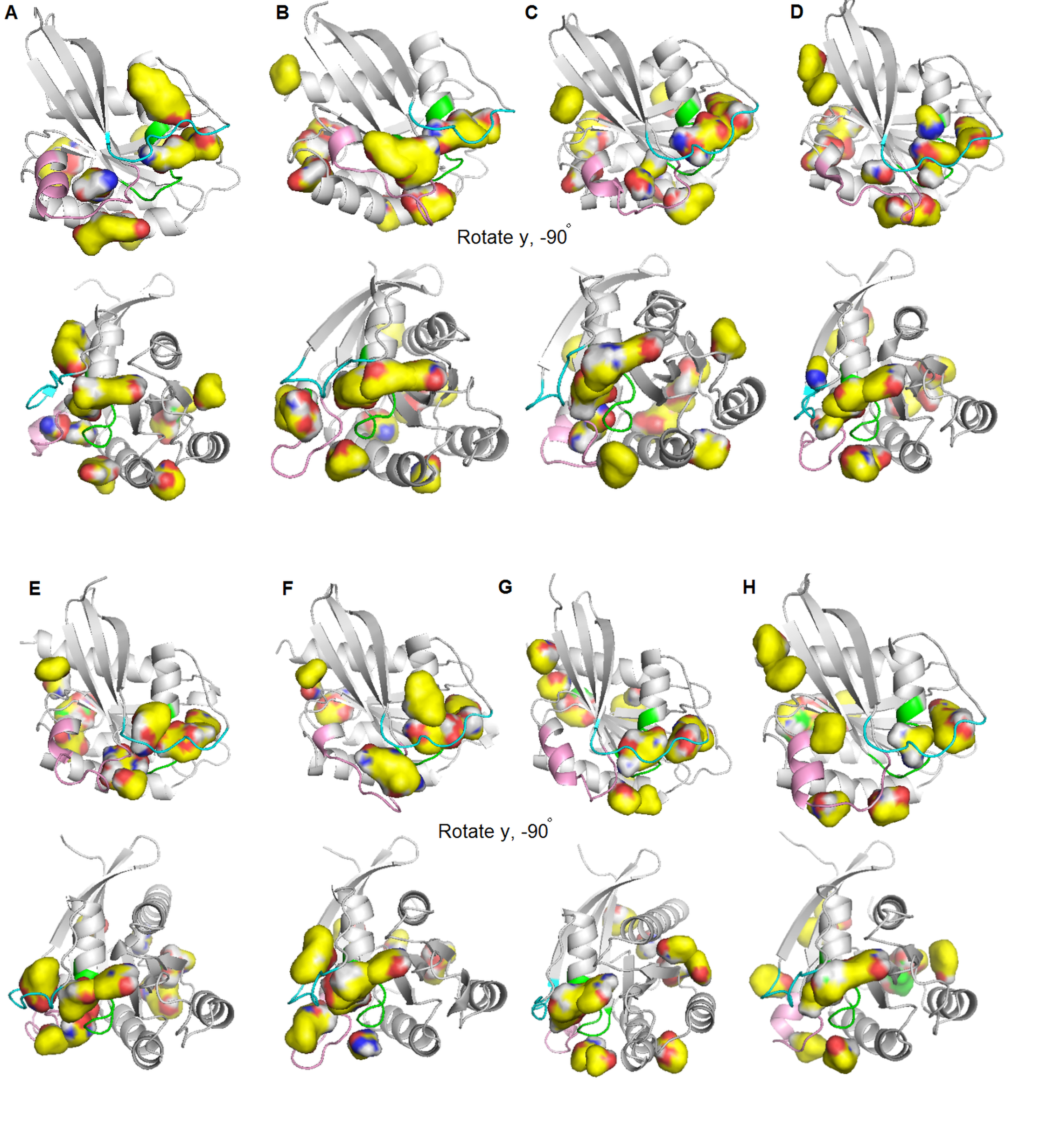

Supplement: S12 Fig — Cartoon representations of Rab11 representative structures (A) 1OIV_A, (B) 1YZK_A, (C) 4C4P_A, (D) 4LX0_C and their rotated views are shown in the first and second rows, respectively. Cartoon representations of Rab11 representative structures (E) 4OJK_A, (F) 4UJ5_B, (G) 5C46_F, (H) 5JCZ_D and their rotated views are shown in the third and fourth rows, respectively. In the cartoon representations, probes occupying identified binding sites are shown in surface and colored yellow, blue, and red for C, N, and O atoms, respectively. Switches 1 and 2 are colored in cyan and pink, respectively. P loop is shown in green. (TIF) [file pone.0198632.s012.tif]

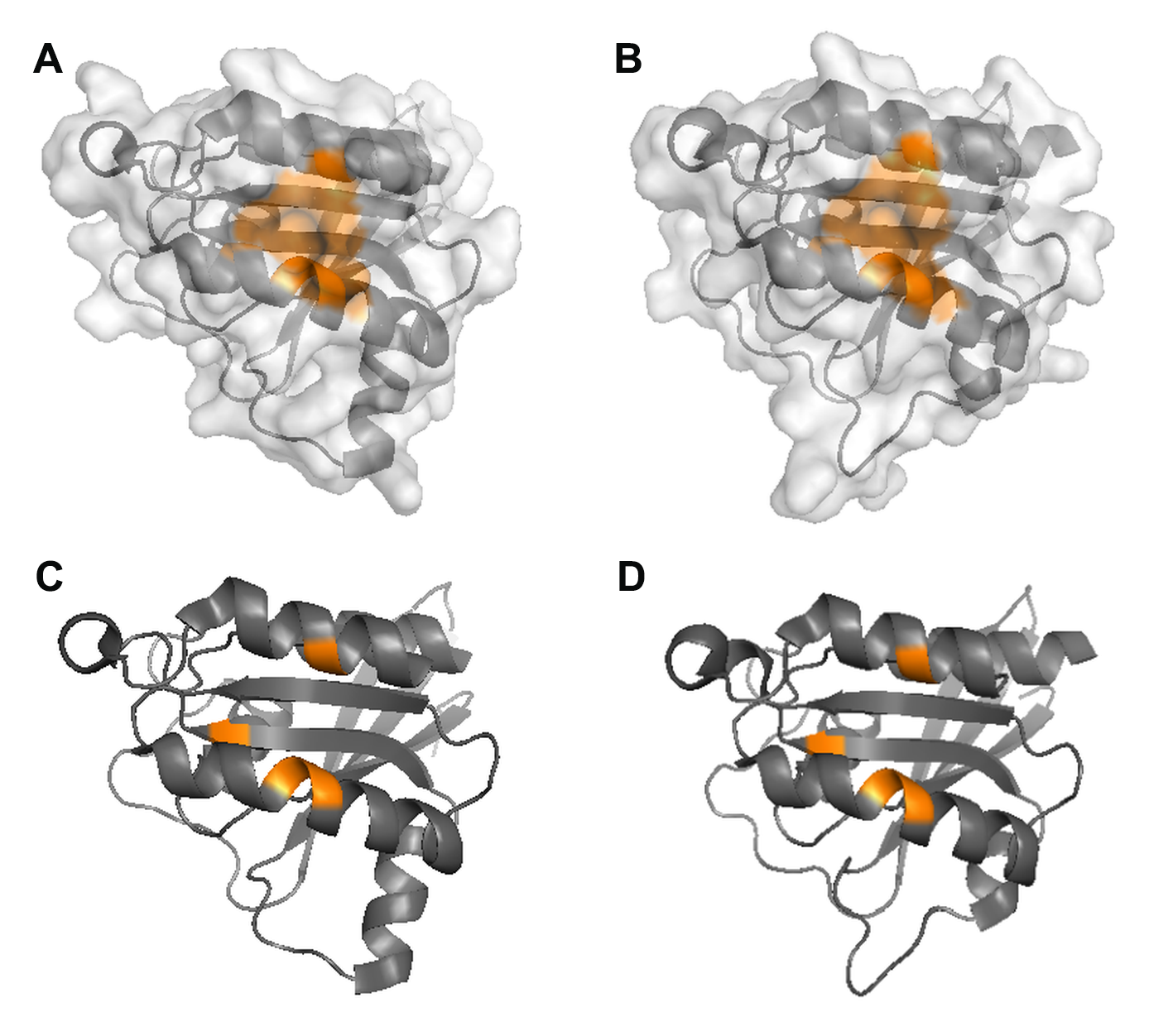

Supplement: S13 Fig — The figure shows the binding site formed by residues Y99, F142, V102 and E103 (colored in orange) in PDB entries 1OIV_A ((A) and (C)) and 1YZK_A ((B) and (D)). The top panel shows the transparent surface of the PDB entries and the bottom panel shows the cartoon representation of the PDB entries, aligned in the same orientation. (TIF) [file pone.0198632.s013.tif]

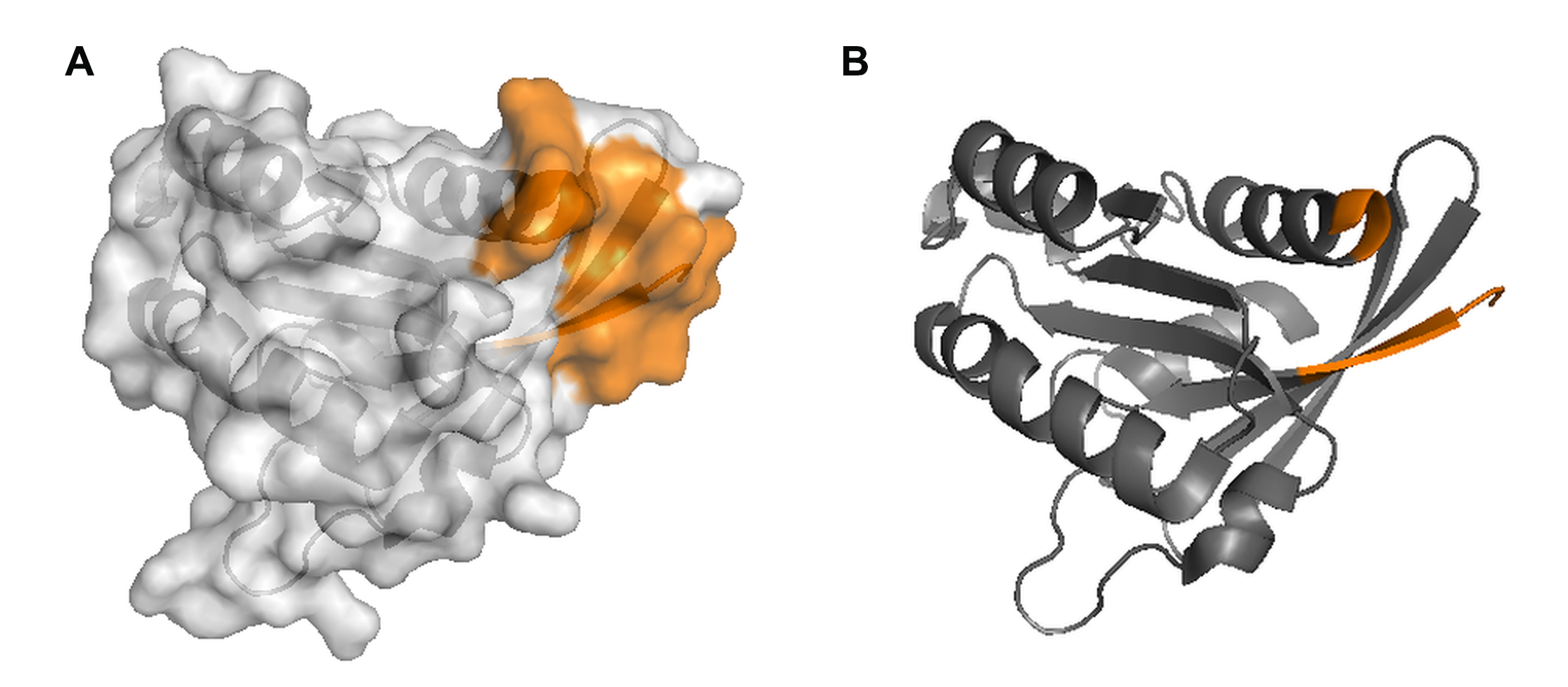

Supplement: S14 Fig — In (A), transparent surface of 1YZK_A is shown and in (B), cartoon representation of 1YZK_A is shown. The binding site is colored in orange. The figures are aligned in the same orientation. (TIF) [file pone.0198632.s014.tif]

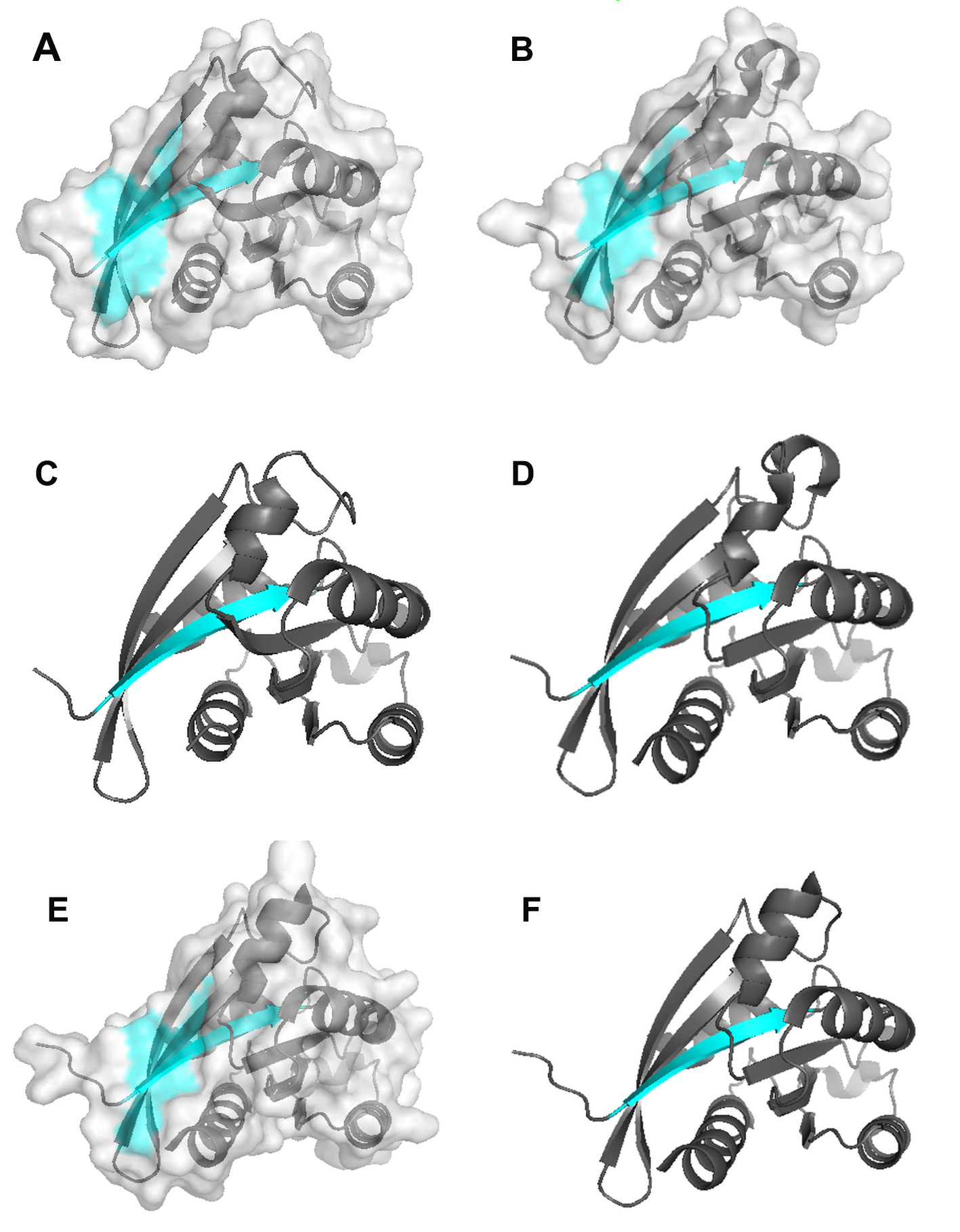

Supplement: S15 Fig — The transparent surface of PDB entries 4C4P_A, 4LX0_C and 5JCZ_D are shown in (A), (B) and (E), respectively. The cartoon representations of 4C4P_A, 4LX0_C and 5JCZ_D are shown in (C), (D) and (F), respectively. The binding site is colored in cyan. The figures are aligned in the same orientation. (TIF) [file pone.0198632.s015.tif]

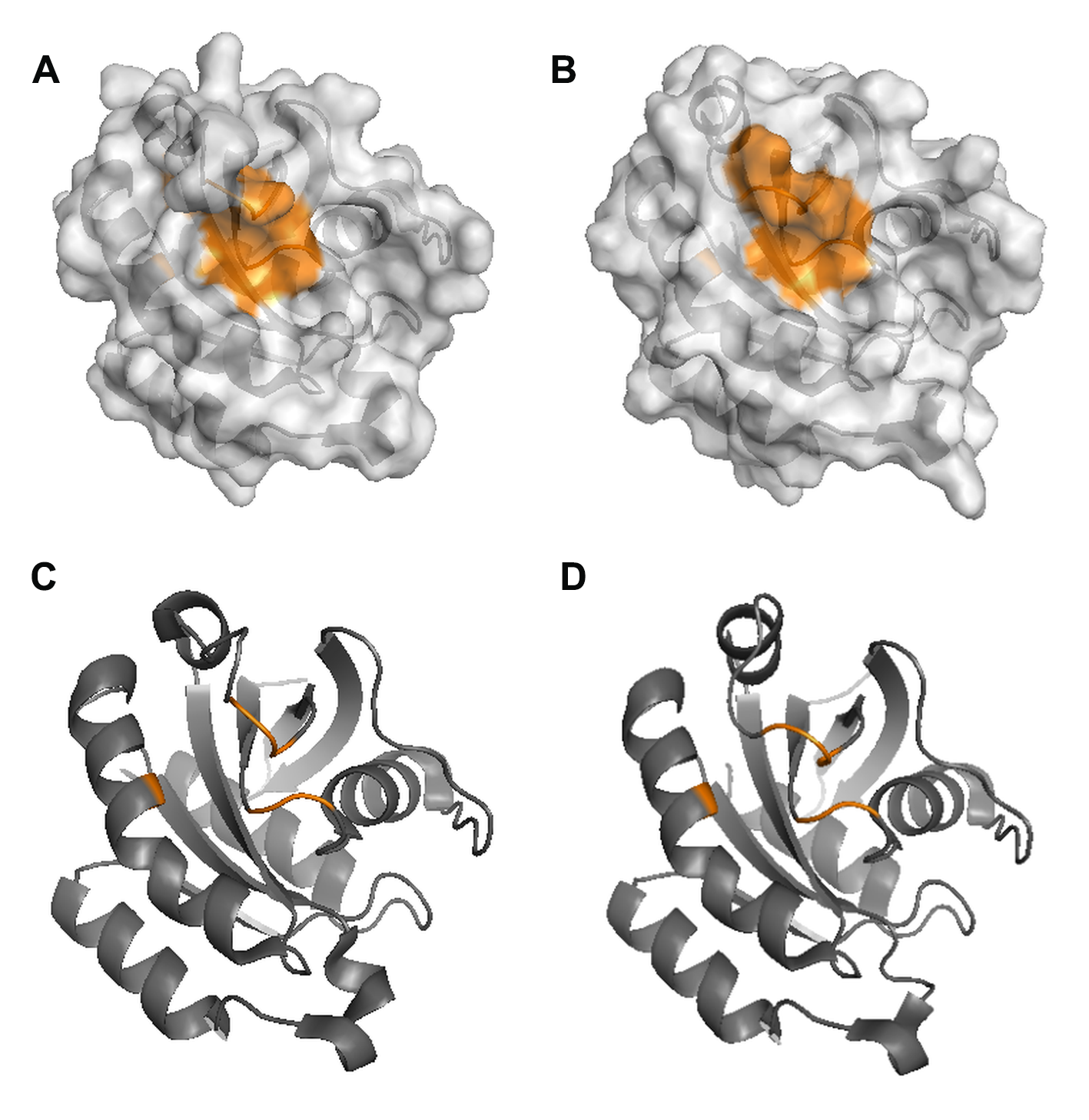

Supplement: S16 Fig — The transparent surface of PDB entries 1YZK_A and 4C4P_A are shown in (A) and (B), respectively. The cartoon representations of 1YZK_A and 4C4P_A are shown in (C) and (D), respectively. The binding site is colored in orange. The figures are aligned in the same orientation. (TIF) [file pone.0198632.s016.tif]

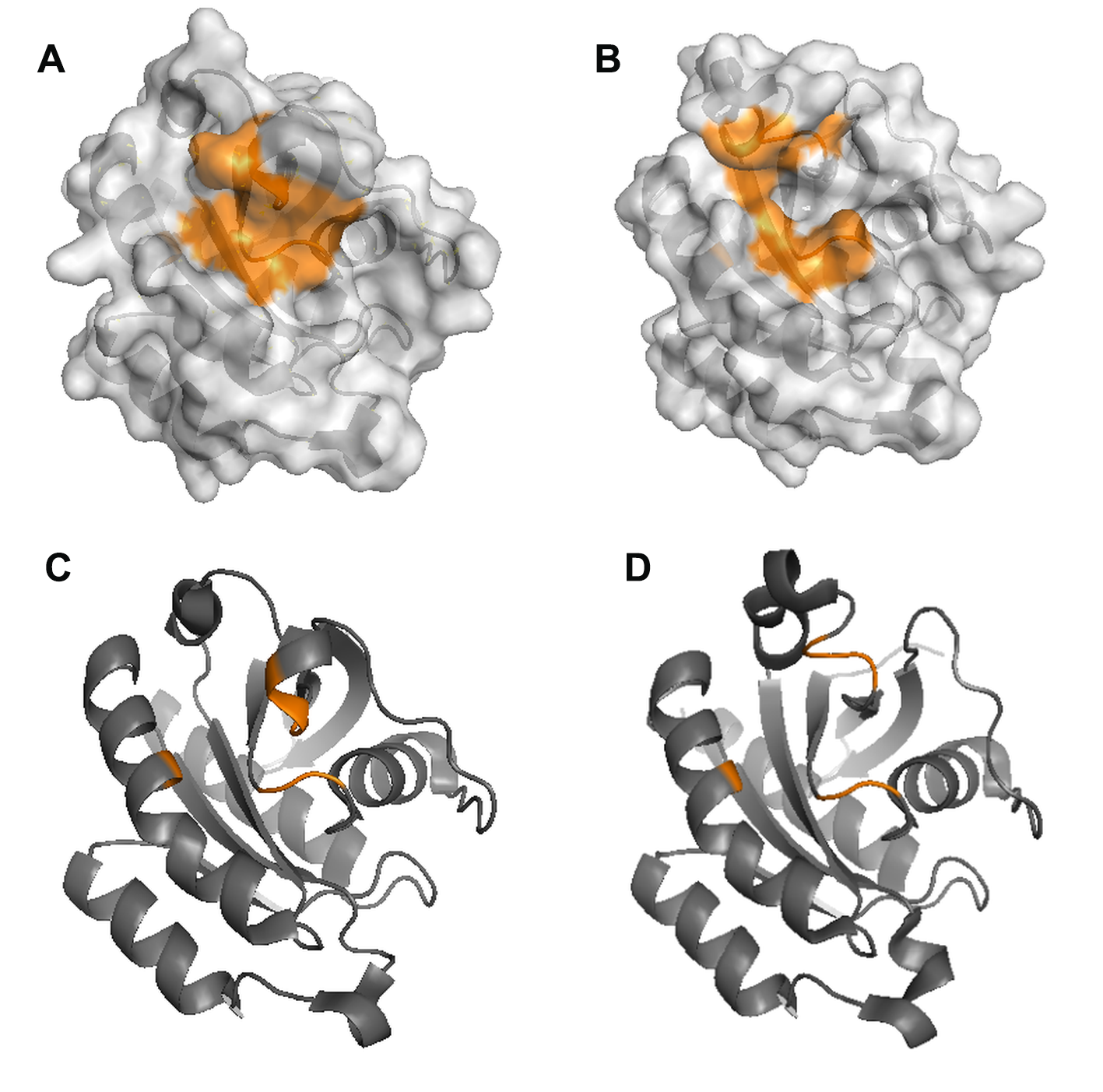

Supplement: S17 Fig — The transparent surface of PDB entries 4LX0_C and 4OJK_A are shown in (A) and (B), respectively. The cartoon representations of 4LX0_C and 4OJK_A are shown in (C) and (D), respectively. The binding site is colored in orange. The figures are aligned in the same orientation. (TIF) [file pone.0198632.s017.tif]

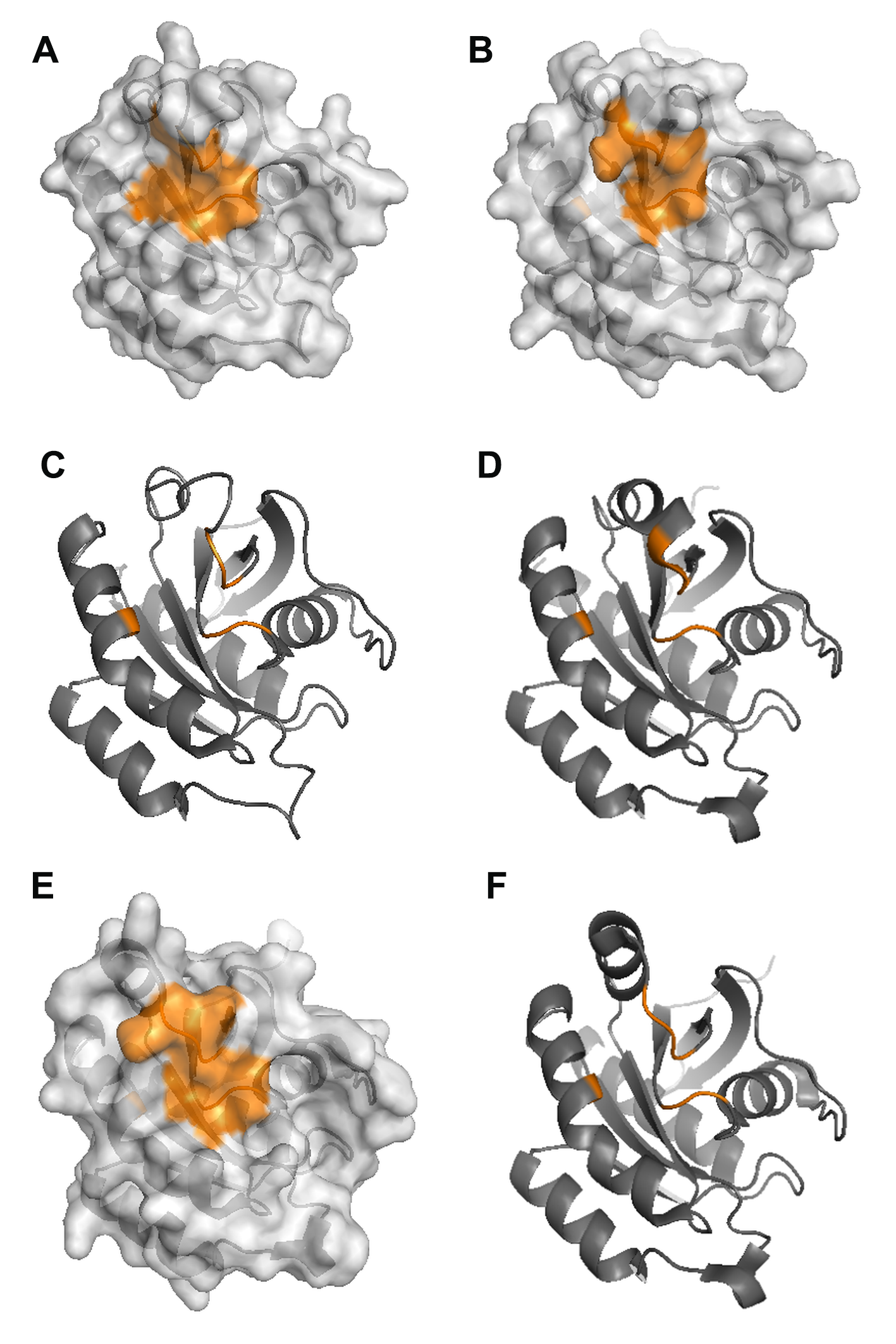

Supplement: S18 Fig — The transparent surface of PDB entries 4UJ5_B, 5C46_F and 5JCZ_D are shown over their secondary structures in (A), (B) and (E), respectively. The cartoon representations of 4UJ5_B, 5C46_F and 5JCZ_D are shown in (C), (D) and (F), respectively. The binding site is colored in orange. The figures are aligned in the same orientation. (TIF) [file pone.0198632.s018.tif]

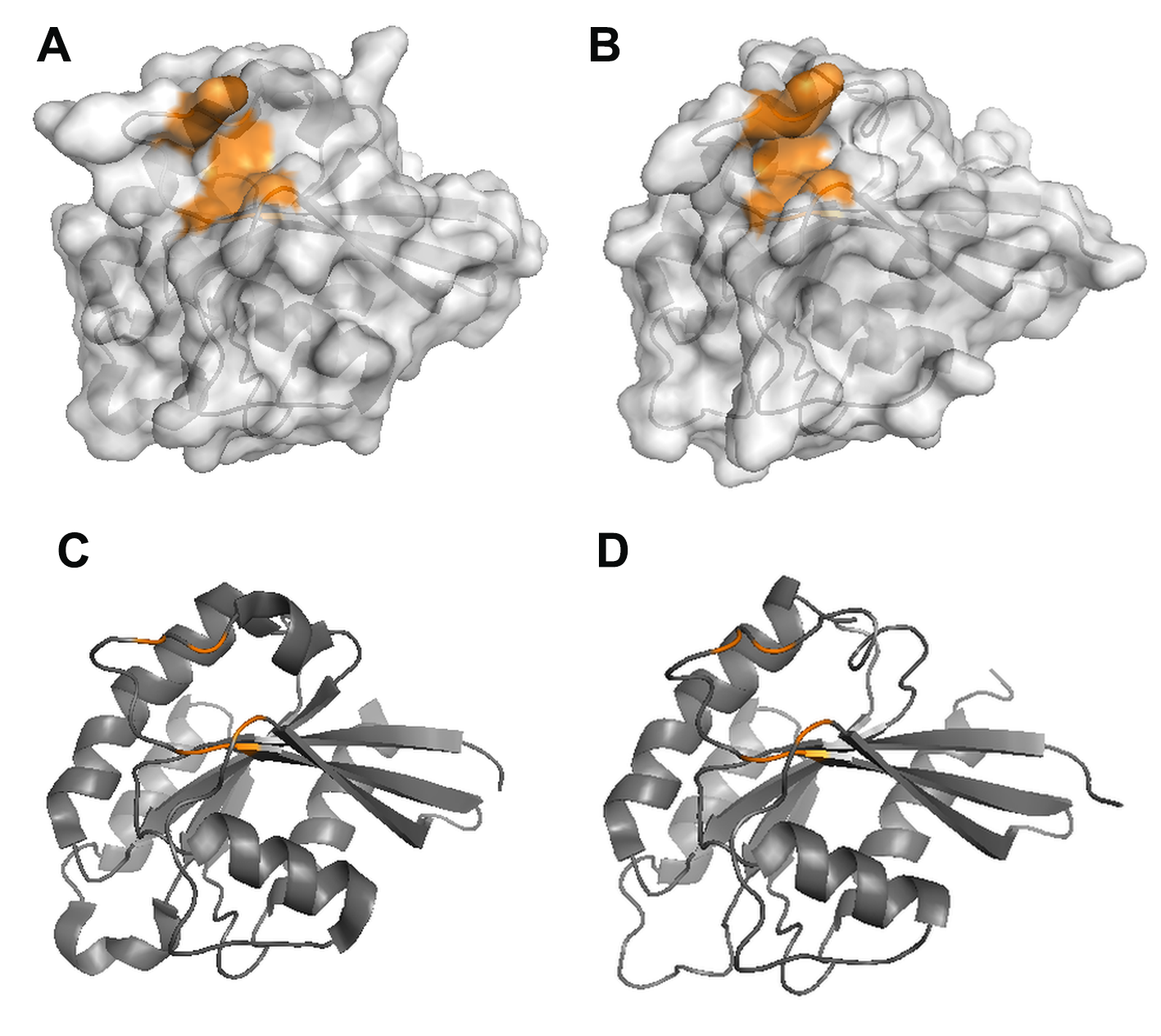

Supplement: S19 Fig — The transparent surface of PDB entries 1YZK_A and 4UJ5_B are shown over their secondary structures in (A) and (B), respectively. The cartoon representations of 1YZK_A and 4UJ5_B are shown in (C) and (D), respectively. The binding site is colored in orange. The figures are aligned in the same orientation. (TIF) [file pone.0198632.s019.tif]

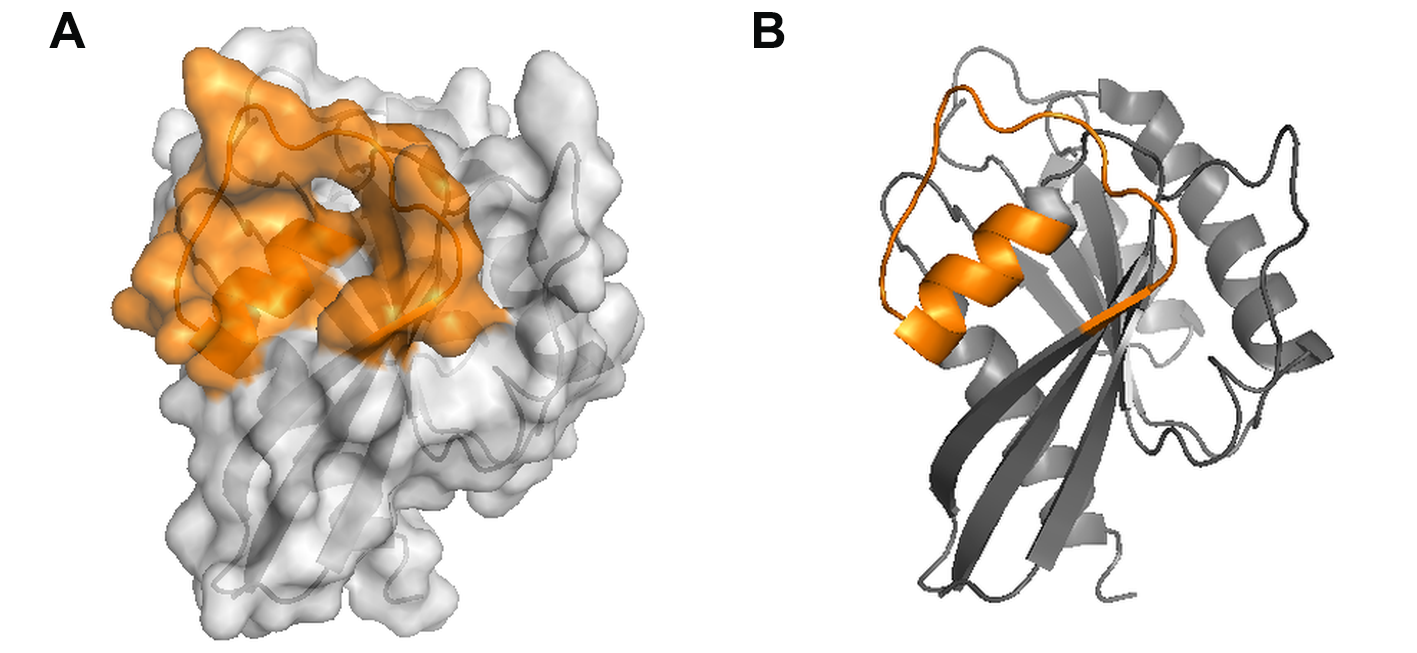

Supplement: S20 Fig — In (A), transparent surface of 4UJ5_B is shown and in (B), cartoon representation of 4UJ5_B is shown. The binding site is colored in orange. The figures are aligned in the same orientation. (TIF) [file pone.0198632.s020.tif]

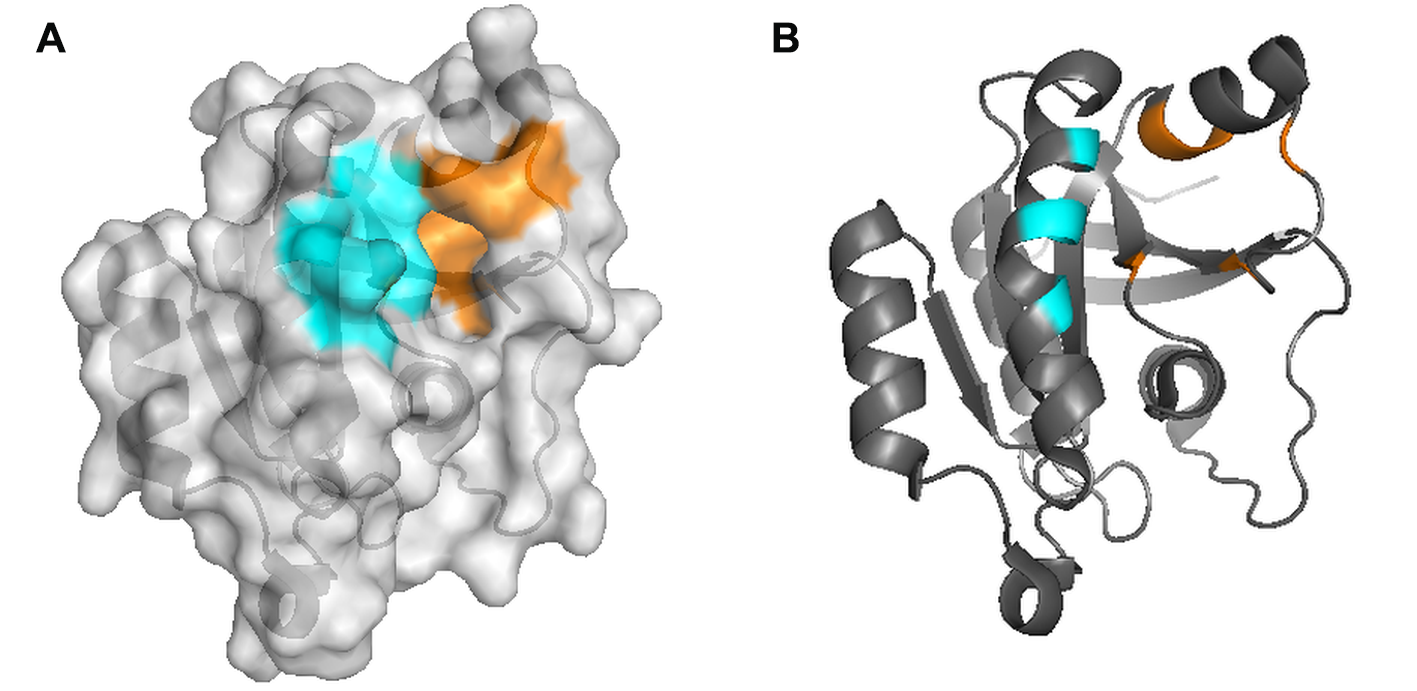

Supplement: S21 Fig — In (A), transparent surface of 1OIV_A is shown and in (B), cartoon representation of 1OIV_A is shown. In (A) and (B), the binding site formed by residues I17, T67, R72, Y80, Y81, W105, E108 and the binding site formed by residues N101, R104, W105 and E108 are colored in cyan and orange, respectively. (TIF) [file pone.0198632.s021.tif]

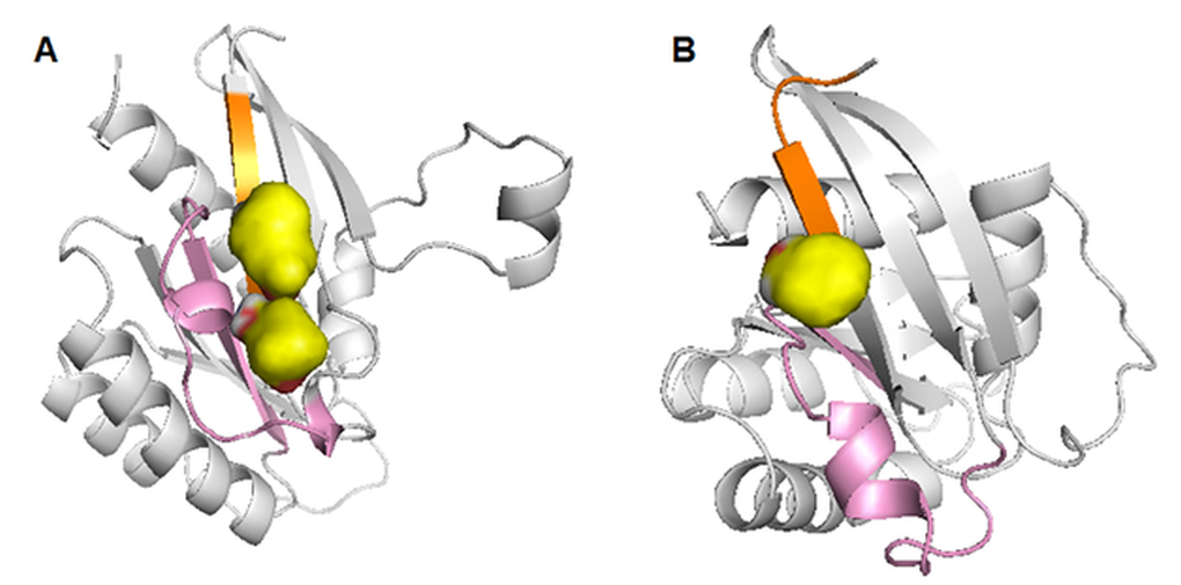

Supplement: S22 Fig — Binding sites formed by residues 10–17 in Rab1 and Rab11 structures are shown. RabSF1 (residues 8–13) and Switch 2 (Residues 68–79) are colored in orange and pink, respectively. Probes occupying identified binding sites are shown in surface and colored yellow, blue, and red for C, N, and O atoms, respectively. (TIF) [file pone.0198632.s022.tif]

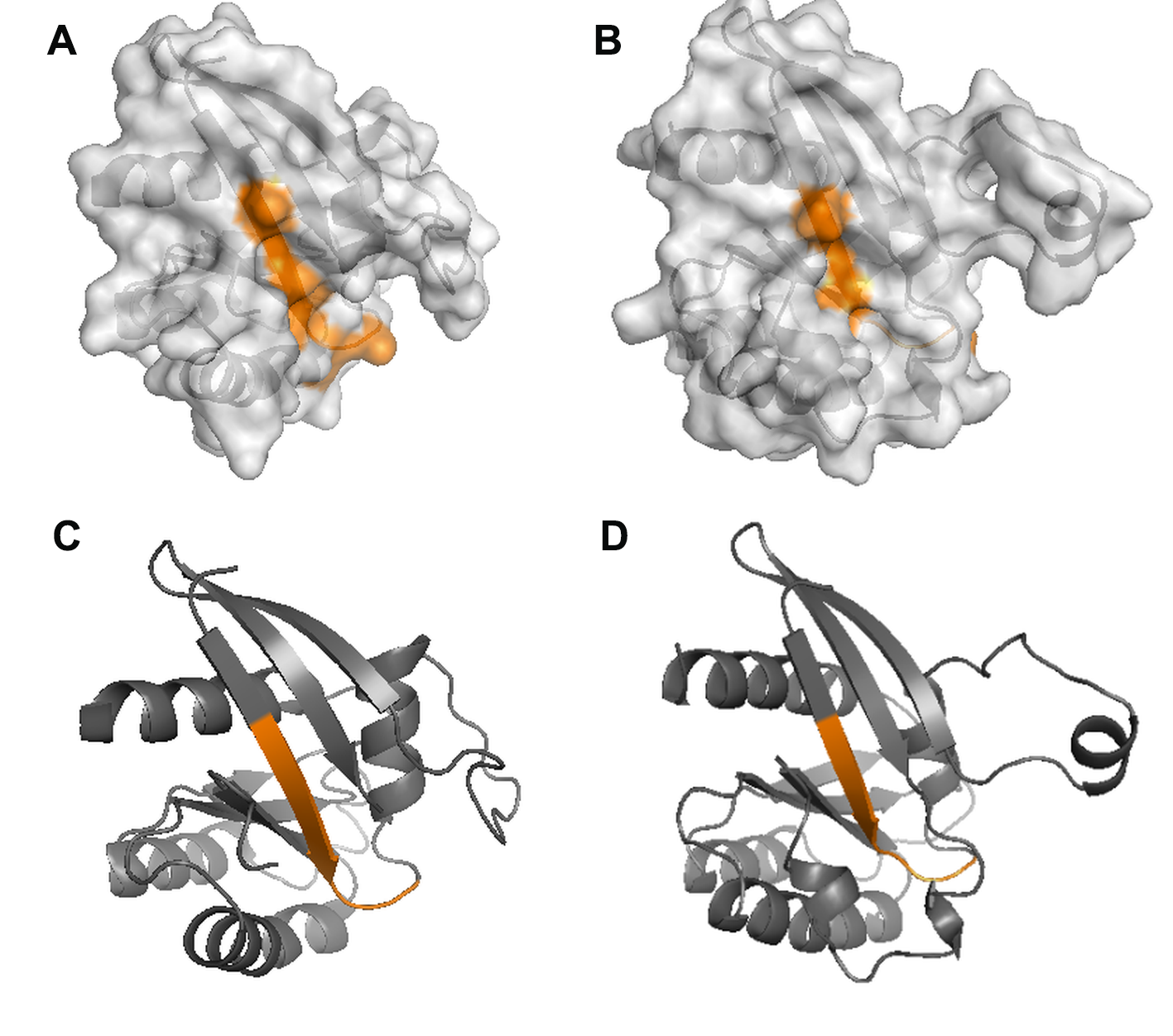

Supplement: S23 Fig — The transparent surface of PDB entries 2FOL_A and 2WWX_A are shown in (A) and (B), respectively. The cartoon representations of 2FOL_A and 2WWX_A are shown in (C) and (D), respectively. The binding site is colored in orange. The figures are aligned in the same orientation. (TIF) [file pone.0198632.s023.tif]

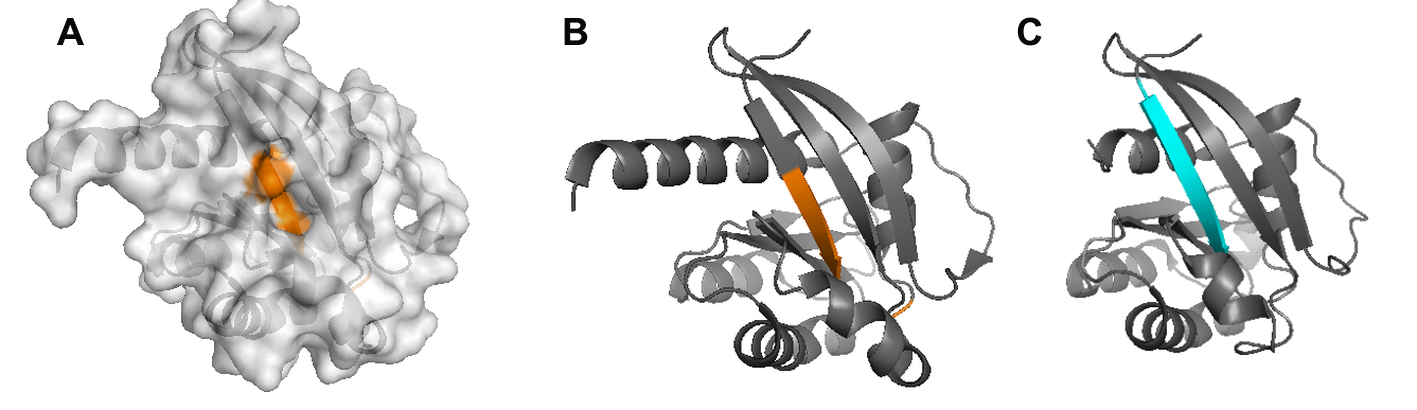

Supplement: S24 Fig — In (A), transparent surface of PDB entry 4I1O_E is shown and in (B), cartoon representation of the PDB entry 4I1O is shown. The binding site formed by residues K10-S17is colored in orange in (A) and (B). In (C), the similar binding site in PDB entry 4C4P_A formed by residues Y10-I17, is colored in cyan on its cartoon representation. The figures are aligned in the same orientation. The binding site in 4C4P_A is shown in its 3D structure in S15 Fig. (TIF) [file pone.0198632.s024.tif]

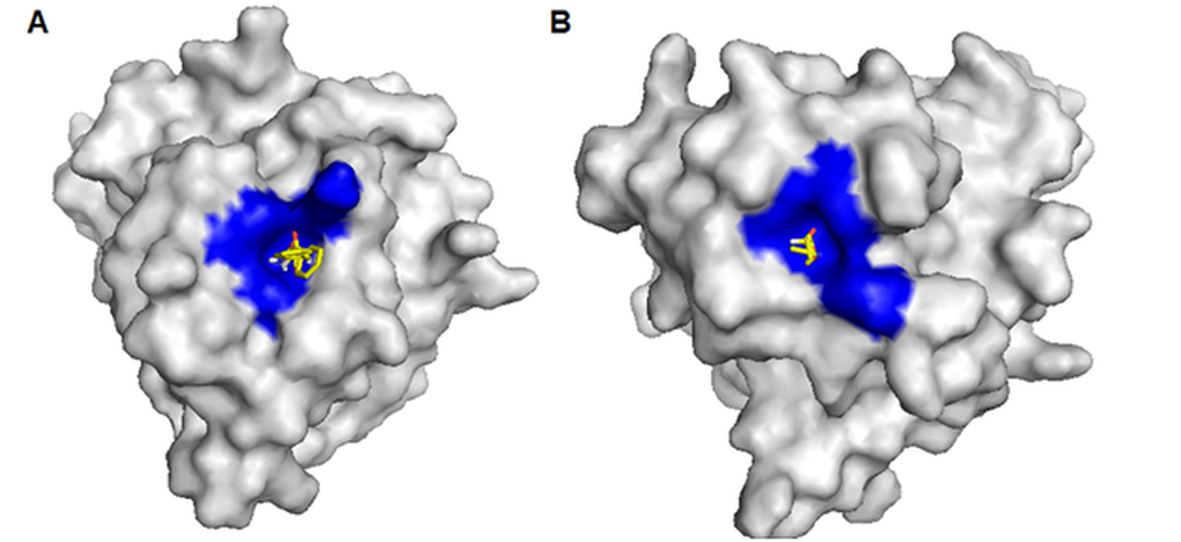

Supplement: S25 Fig — Pockets formed by residues 99, 102, 103 and 142 in Rab1 and Rab11 are colored in blue. Probes occupying identified binding sites are shown in sticks and colored yellow, blue, and red for C, N, and O atoms, respectively. (TIF) [file pone.0198632.s025.tif]

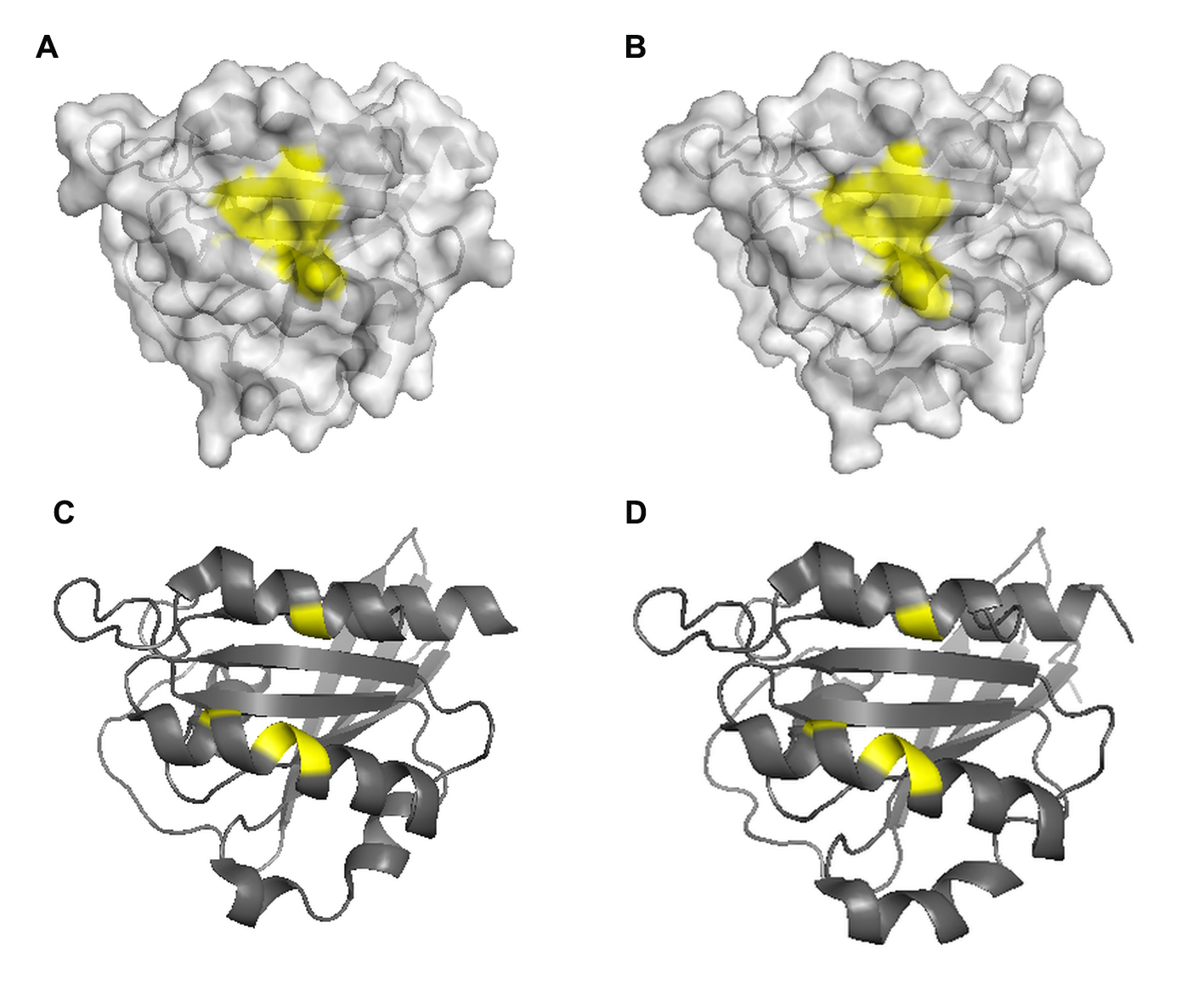

Supplement: S26 Fig — The transparent surface of PDB entries 3TKL_A and 4FMB_D are shown in (A) and (B), respectively. The cartoon representations of 3TKL_A and 4FMB_D are shown in (C) and (D), respectively. The binding site is colored in yellow. The figures are aligned in the same orientation. (TIF) [file pone.0198632.s026.tif]

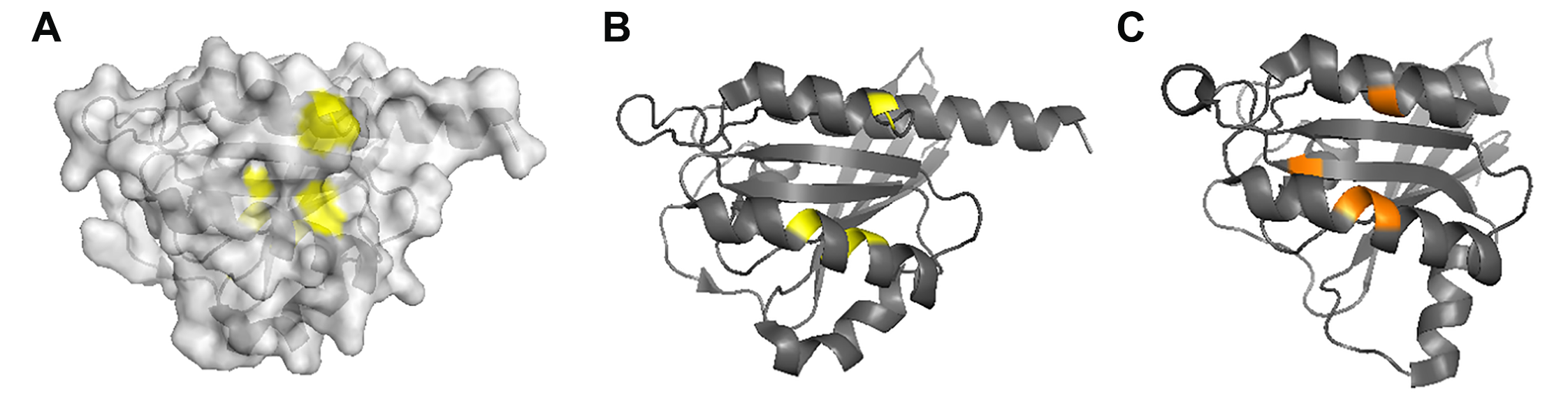

Supplement: S27 Fig — In (A), transparent surface of PDB entry 4I1O_E is shown and in (B), cartoon representation of the PDB entry is shown. The binding site formed by residues V99, W102, L103 and S142 in 4I1O_E is colored in yellow in (A) and (B). In (C), the similar binding site in PDB entry 1OIV_A formed by residues Y99, V102, E103 and F142, is colored in orange on its cartoon representation. The figures are aligned in the same orientation. The binding site is shown in the 3D representation of 1OIV_A in S13 Fig. (TIF) [file pone.0198632.s027.tif]

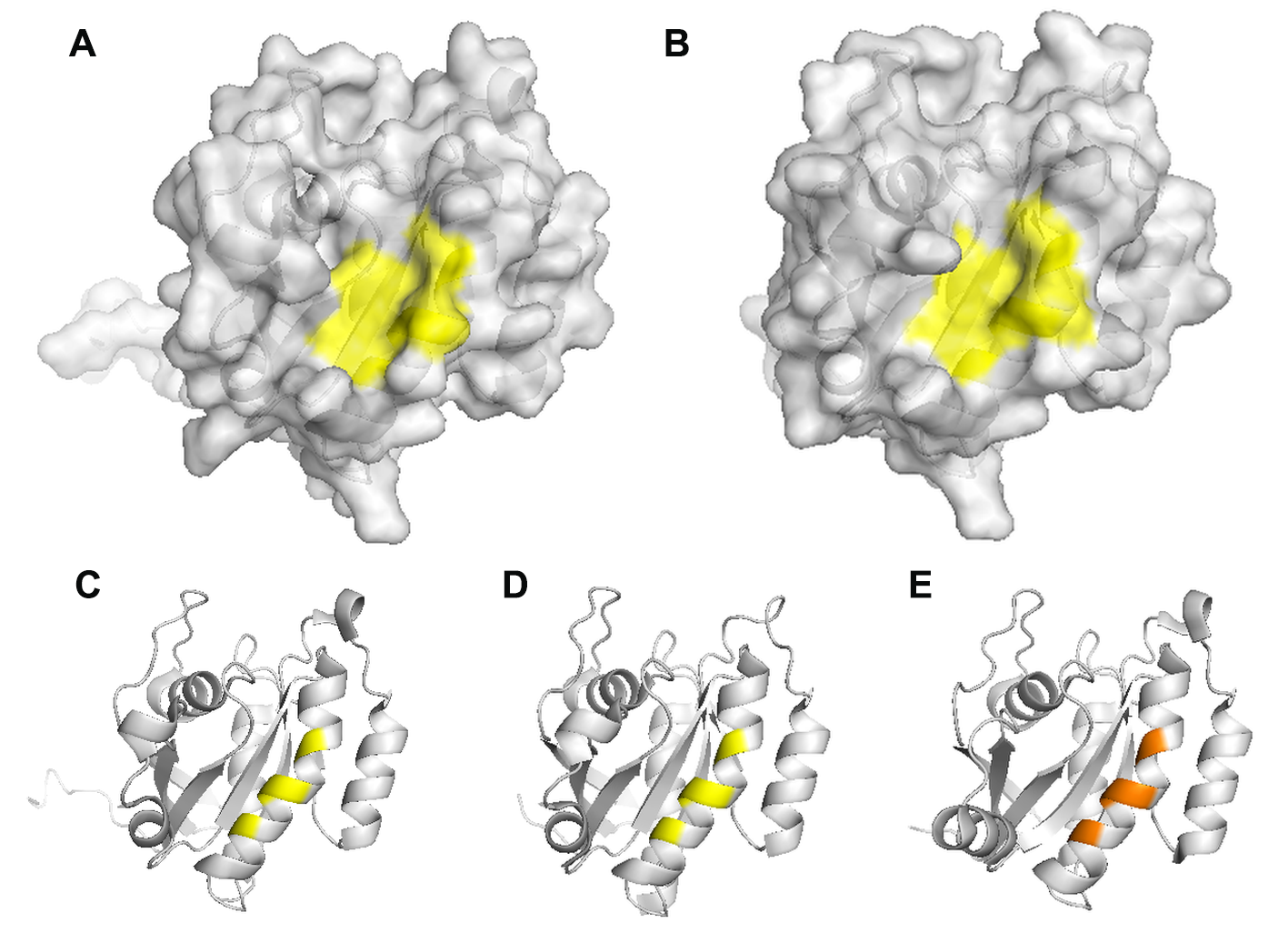

Supplement: S28 Fig — The transparent surface of PDB entries 3SFV_A (Rab1) and 3TKL_A (Rab1) are shown over their secondary structures in (A) and (B), respectively. The cartoon representations of 3SFV_A and 3TKL_A are shown in (C) and (D), respectively. The binding site formed by residues N101, Q104, W105 and E108 is colored in yellow. In (E), the similar binding site formed by residues N101, R104, W105 and E108 in PDB entry 1OIV_A (Rab11) is colored in orange on its cartoon representation. The figures are aligned in the same orientation. The binding site is shown in the 3D representation of 1OIV_A in S21 Fig. (TIF) [file pone.0198632.s028.tif]

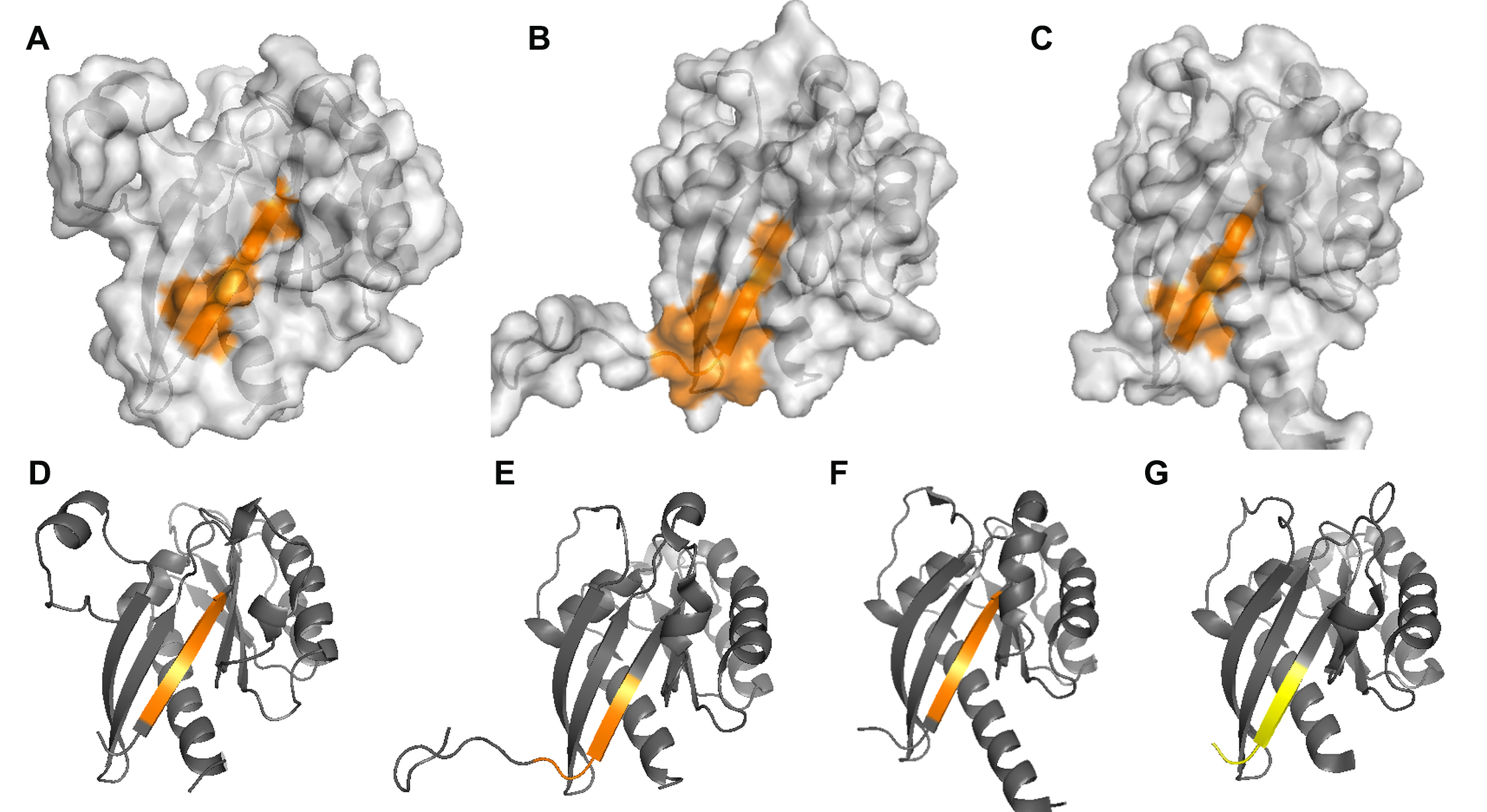

Supplement: S29 Fig — The transparent surface of PDB entries 2WWX_A (Rab1), 3SFV_A (Rab1) and, 4I1O_E (Rab1) are shown in (A), (B) and (C), respectively. The cartoon representations of 2WWX_A, 3SFV_A and 4I1O_E are shown in (D), (E) and (F), respectively. Residues 8–13 in Rab1 is colored in orange in (A)-(F). In (G), residues Y8-K13 in PDB entry 1YZK_A (Rab11) is colored in yellow on its cartoon representation. The figures are aligned in the same orientation. The binding site is shown in the 3D representation of 1YZK_A in S18 Fig. (TIF) [file pone.0198632.s029.tif]

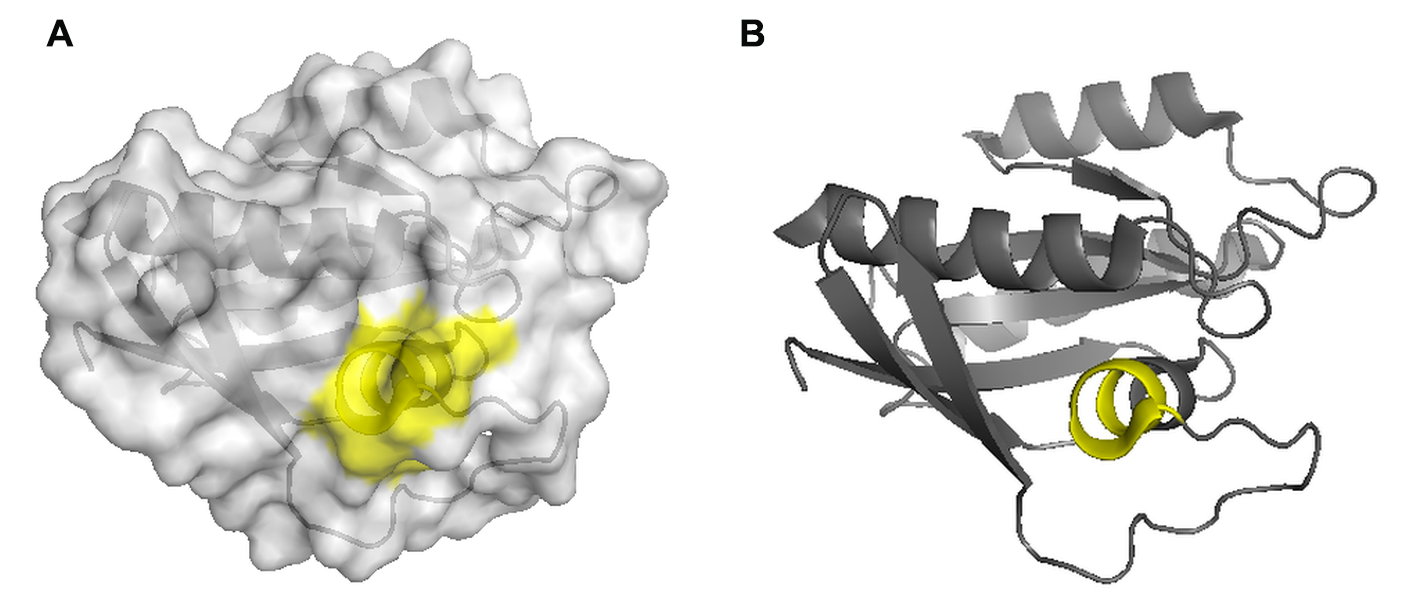

Supplement: S30 Fig — The binding site in Rab1 (PDB entry 2FOL_A) formed by residues L25-D31, which is not observed in any Rab11 representatives, is colored in yellow in its (A) transparent surface and (B) cartoon representation. (TIF) [file pone.0198632.s030.tif]

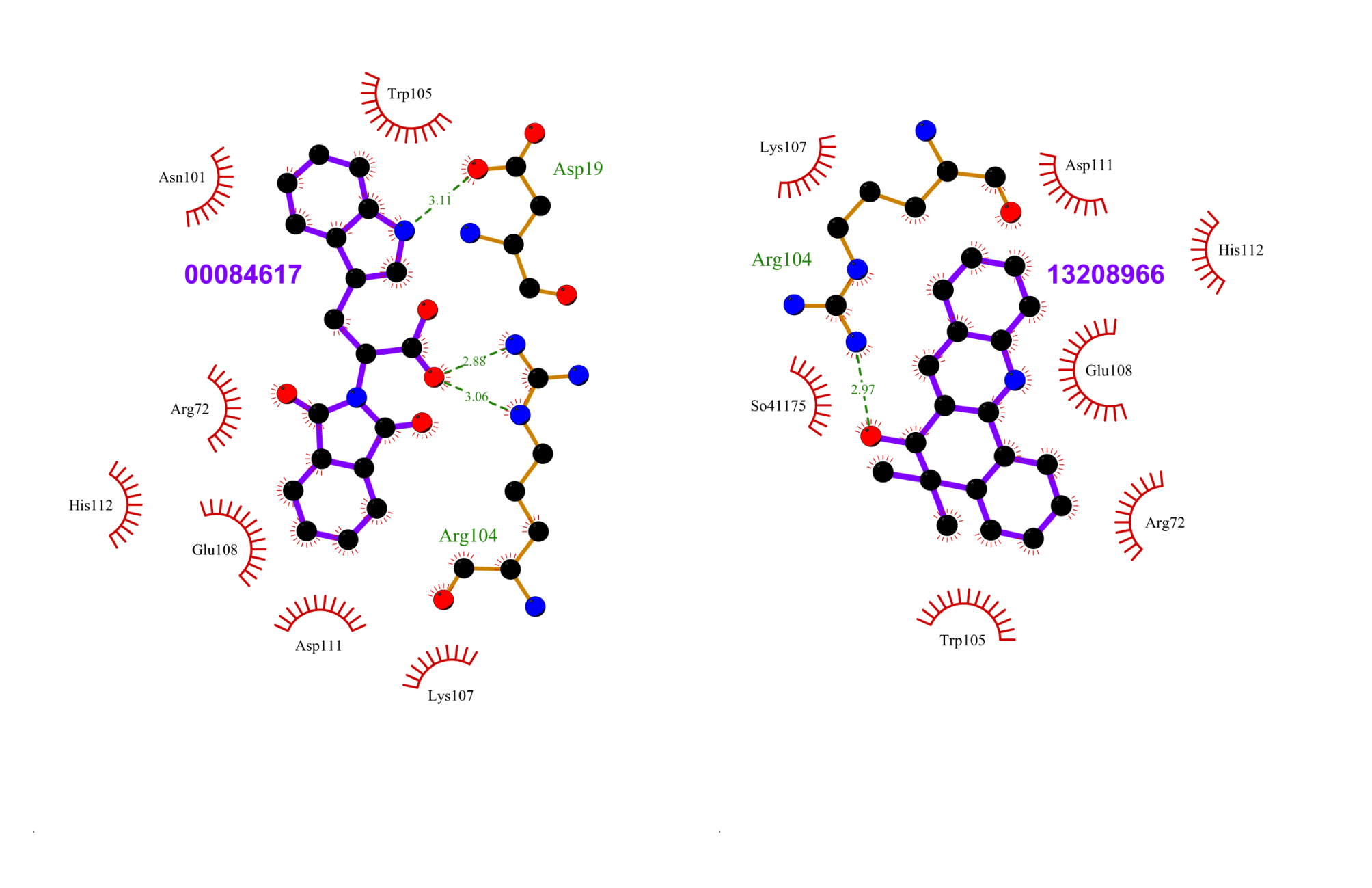

Supplement: S31 Fig — The figure shows the interactions of the ligands (labeled) with the residues of PDB entry 1OIV_A. The ligand and Rab11 side chains are shown in ball-and-stick representation. Black circles denote carbon atoms, red circles denote oxygen atoms, and blue circles denote nitrogen atoms. The ligand bonds are colored in purple. Residues in Rab11 interacting with the ligand are labeled. Hydrogen bonds are shown as green dotted lines. The Rab11 residues making nonbonded contacts with the ligand are shown as spoked arcs. Figures are generated using LigPlot+ [66][67]. (TIF) [file pone.0198632.s031.tif]

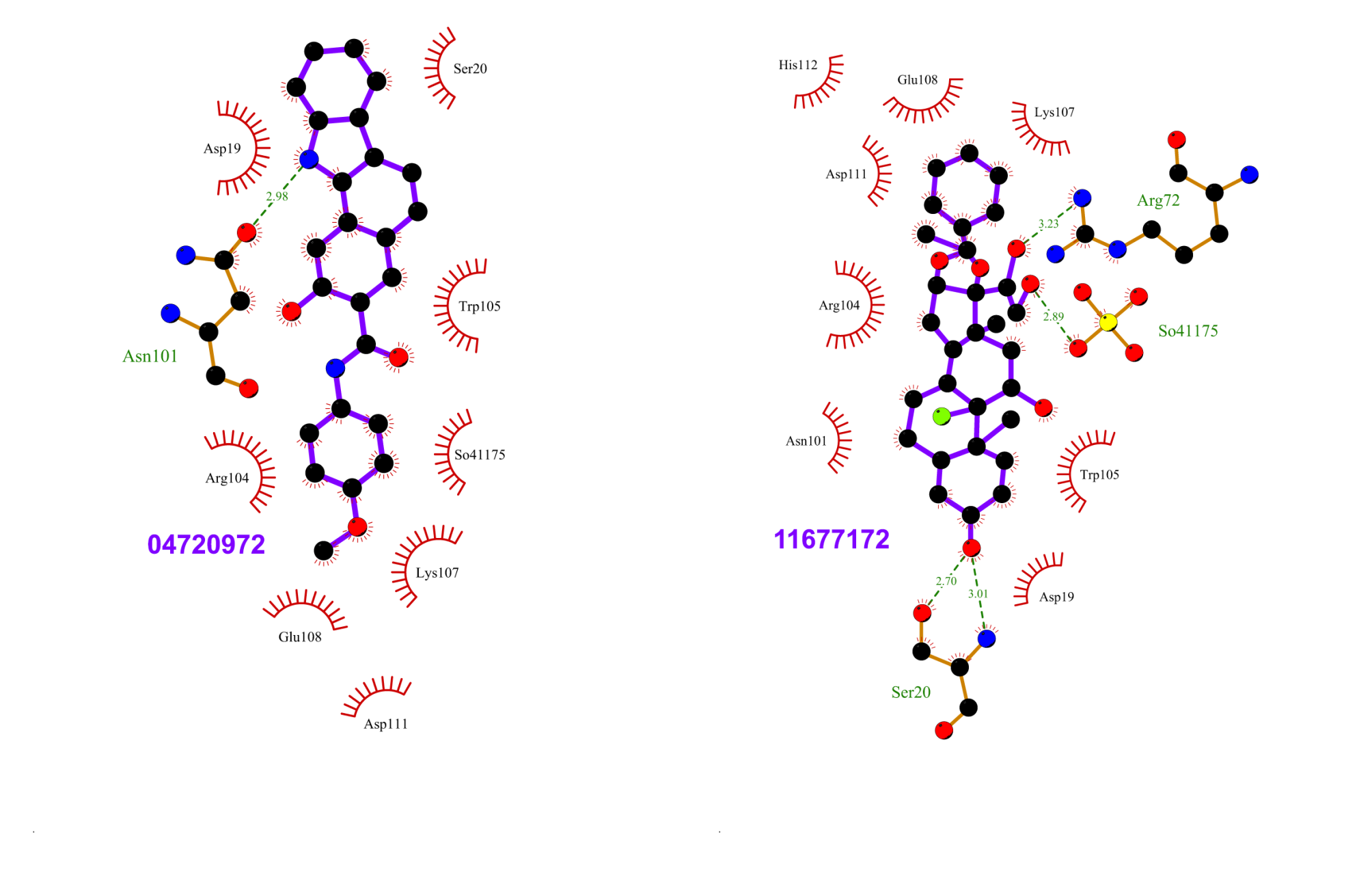

Supplement: S32 Fig — The figure shows the interactions of the ligands (labeled) with the residues of PDB entry 1OIV_A. ZINC11677172 also has interactions with a sulfate ion present near its binding site in the target structure. The ligand and Rab11 side chains are shown in ball-and-stick representation. Black circles denote carbon atoms, red circles denote oxygen atoms, yellow circle denotes sulfur atom, green circle denotes fluorine atom and blue circles denote nitrogen atoms. The ligand bonds are colored in purple. Residues in Rab11 interacting with the ligand are labeled. Hydrogen bonds are shown as green dotted lines. The Rab11 residues making nonbonded contacts with the ligand are shown as spoked arcs. Figures are generated using LigPlot+ [66][67]. (TIF) [file pone.0198632.s032.tif]

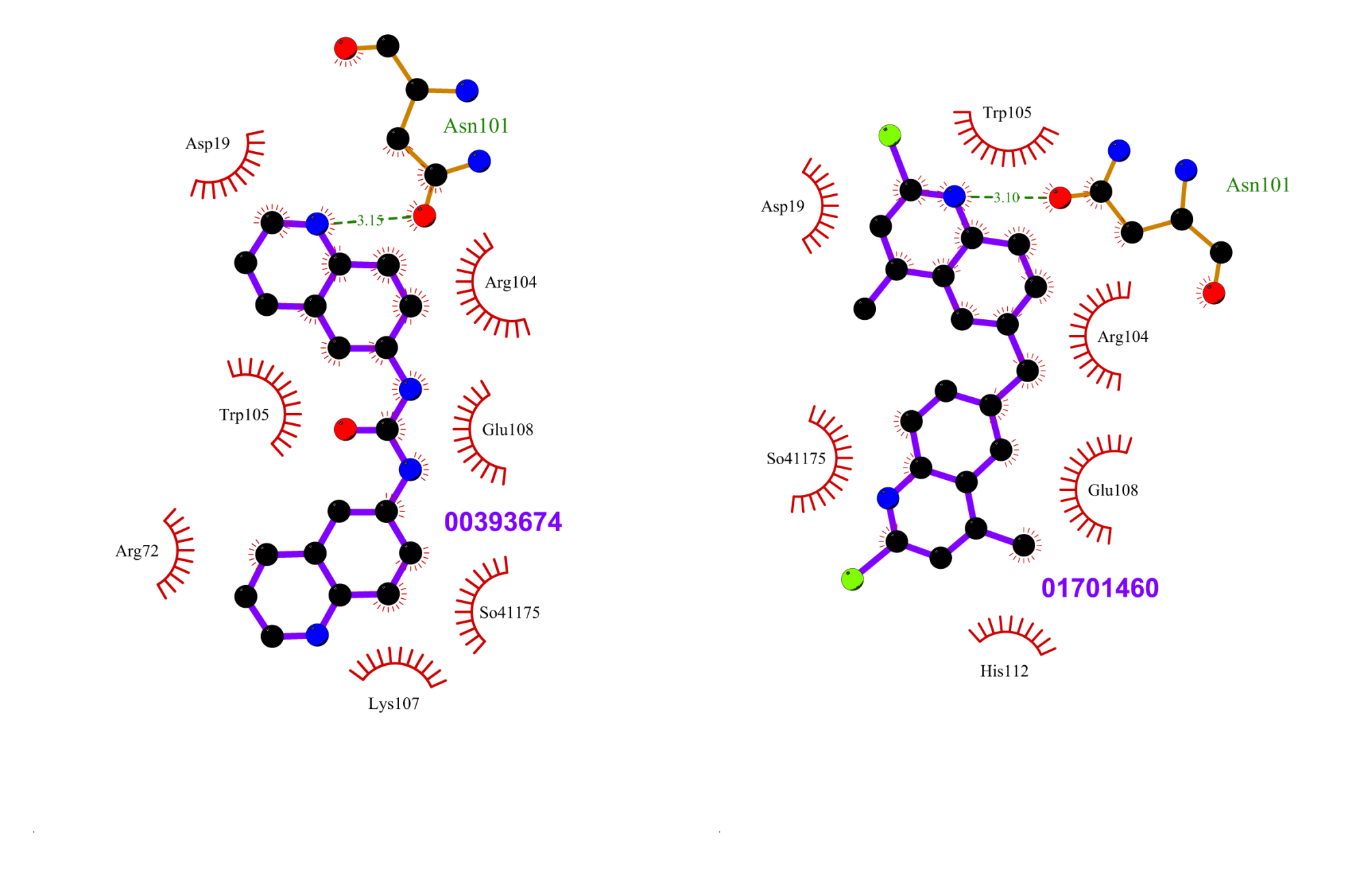

Supplement: S33 Fig — The figure shows the interactions of the ligands (labeled) with the residues of PDB entry 1OIV_A. The ligand and Rab11 side chains are shown in ball-and-stick representation. Black circles denote carbon atoms, red circles denote oxygen atoms, green circles denote chlorine atoms and blue circles denote nitrogen atoms. The ligand bonds are colored in purple. Residues in Rab11 interacting with the ligand are labeled. Hydrogen bonds are shown as green dotted lines. The Rab11 residues making nonbonded contacts with the ligand are shown as spoked arcs. Figures are generated using LigPlot+ [66][67]. (TIF) [file pone.0198632.s033.tif]

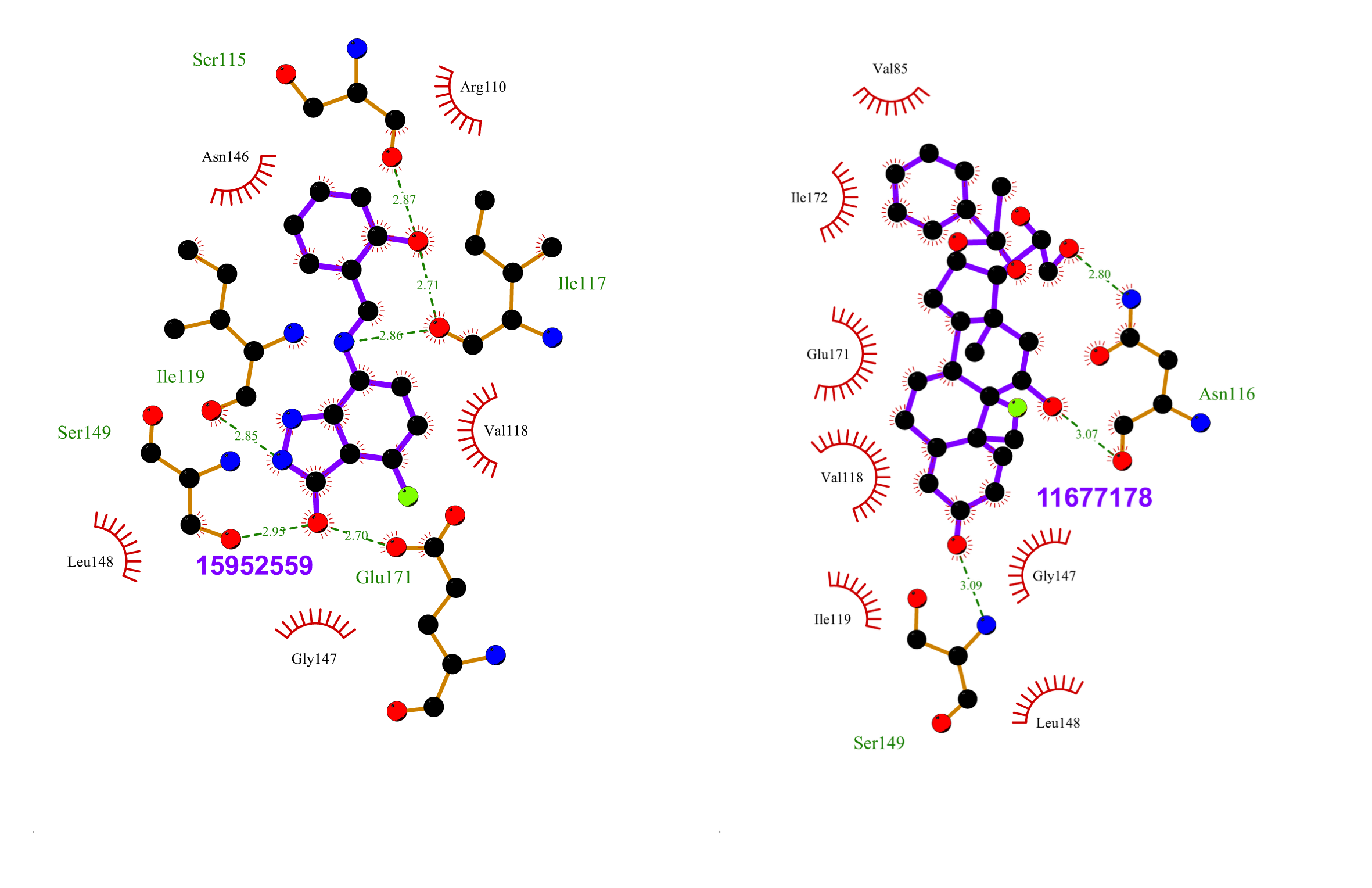

Supplement: S34 Fig — The figure shows the interactions of the ligands (labeled) with the residues of the Rab11 structures in which they scored the best. The ligand and Rab11 side chains are shown in ball-and-stick representation. Black circles denote carbon atoms, red circles denote oxygen atoms, green circles denote fluorine atoms and blue circles denote nitrogen atoms. The ligand bonds are colored in purple. Residues in Rab11 interacting with the ligand are labeled. Hydrogen bonds are shown as green dotted lines. The Rab11 residues making nonbonded contacts with the ligand are shown as spoked arcs. Figures are generated using LigPlot+ [66][67]. (TIF) [file pone.0198632.s034.tif]

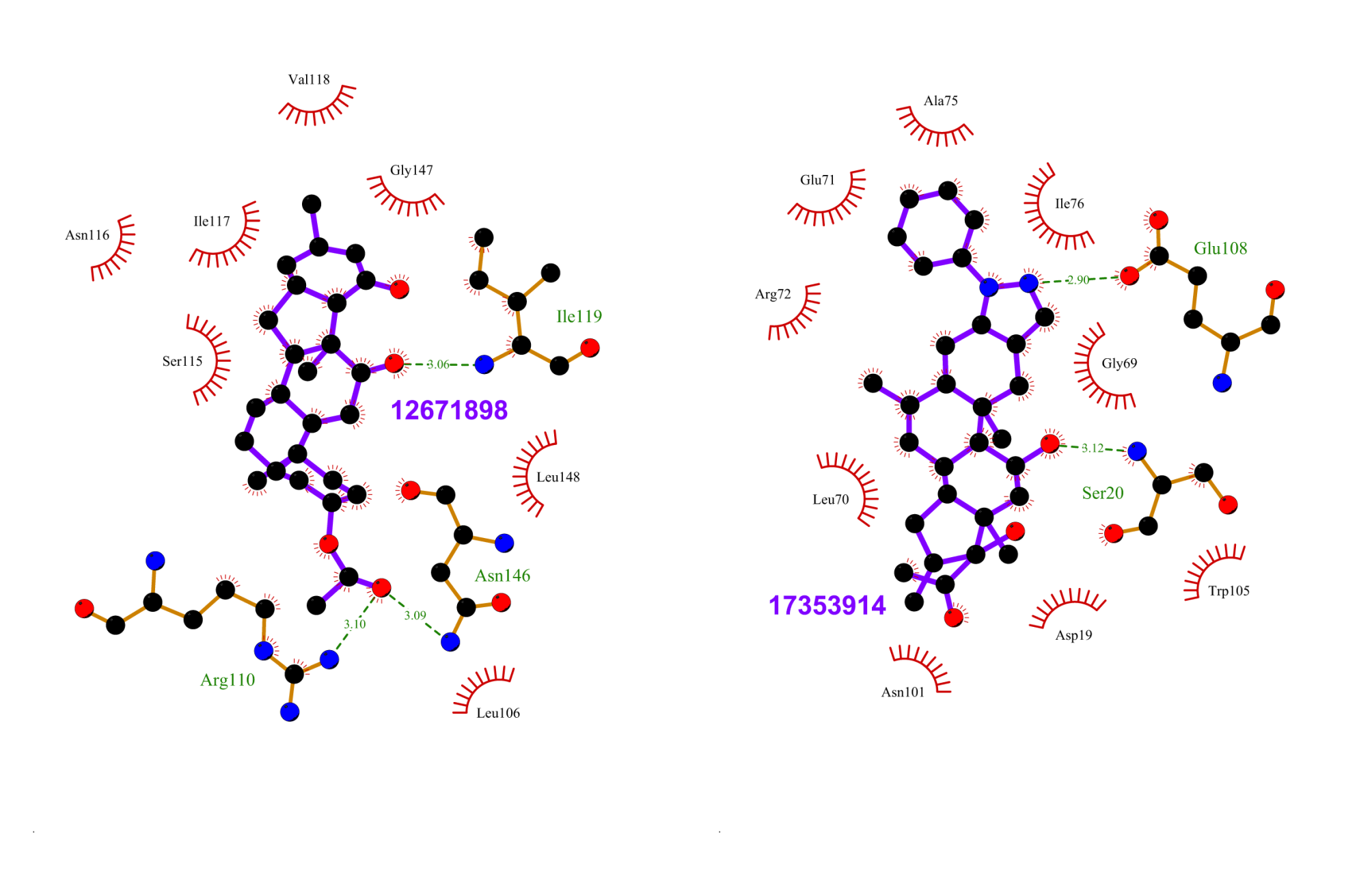

Supplement: S35 Fig — The figure shows the interactions of the ligands (labeled) with the residues of the Rab11 structures in which they scored the best (see Table 3). The ligand and Rab11 side chains are shown in ball-and-stick representation. Black circles denote carbon atoms, red circles denote oxygen atoms and blue circles denote nitrogen atoms. The ligand bonds are colored in purple. Residues in Rab11 interacting with the ligand are labeled. Hydrogen bonds are shown as green dotted lines. The Rab11 residues making nonbonded contacts with the ligand are shown as spoked arcs. Figures are generated using LigPlot+ [66][67]. (TIF) [file pone.0198632.s035.tif]

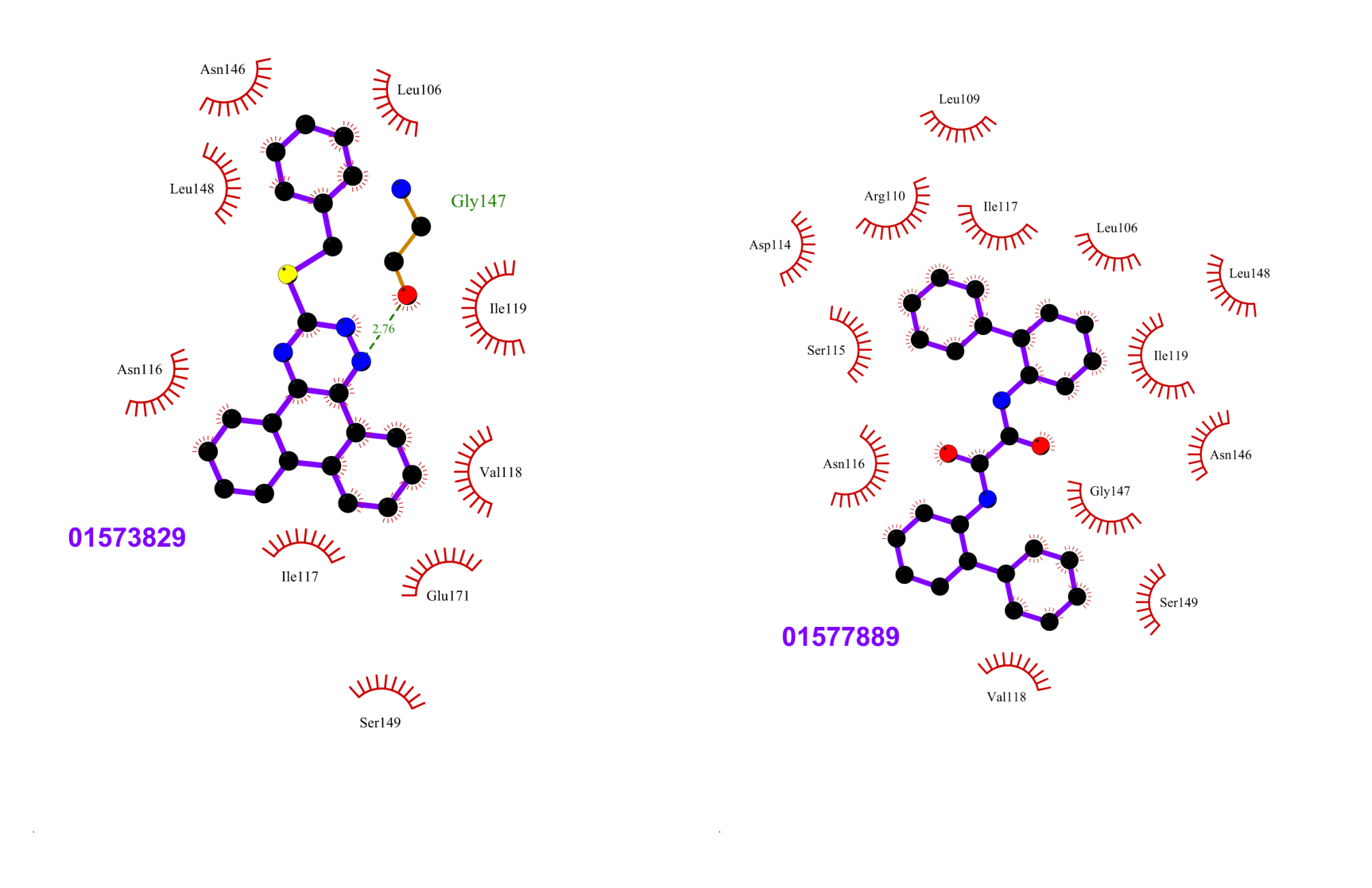

Supplement: S36 Fig — The figure shows the interactions of the ligands (labeled) with the residues of the Rab11 structures in which they scored the best (see Table 3). The ligand and Rab11 side chains are shown in ball-and-stick representation. Black circles denote carbon atoms, red circles denote oxygen atoms, yellow circle denote sulfur atom and blue circles denote nitrogen atoms. The ligand bonds are colored in purple. Residues in Rab11 interacting with the ligand are labeled. Hydrogen bonds are shown as green dotted lines. The Rab11 residues making nonbonded contacts with the ligand are shown as spoked arcs. Figures are generated using LigPlot+ [66][67]. (TIF) [file pone.0198632.s036.tif]

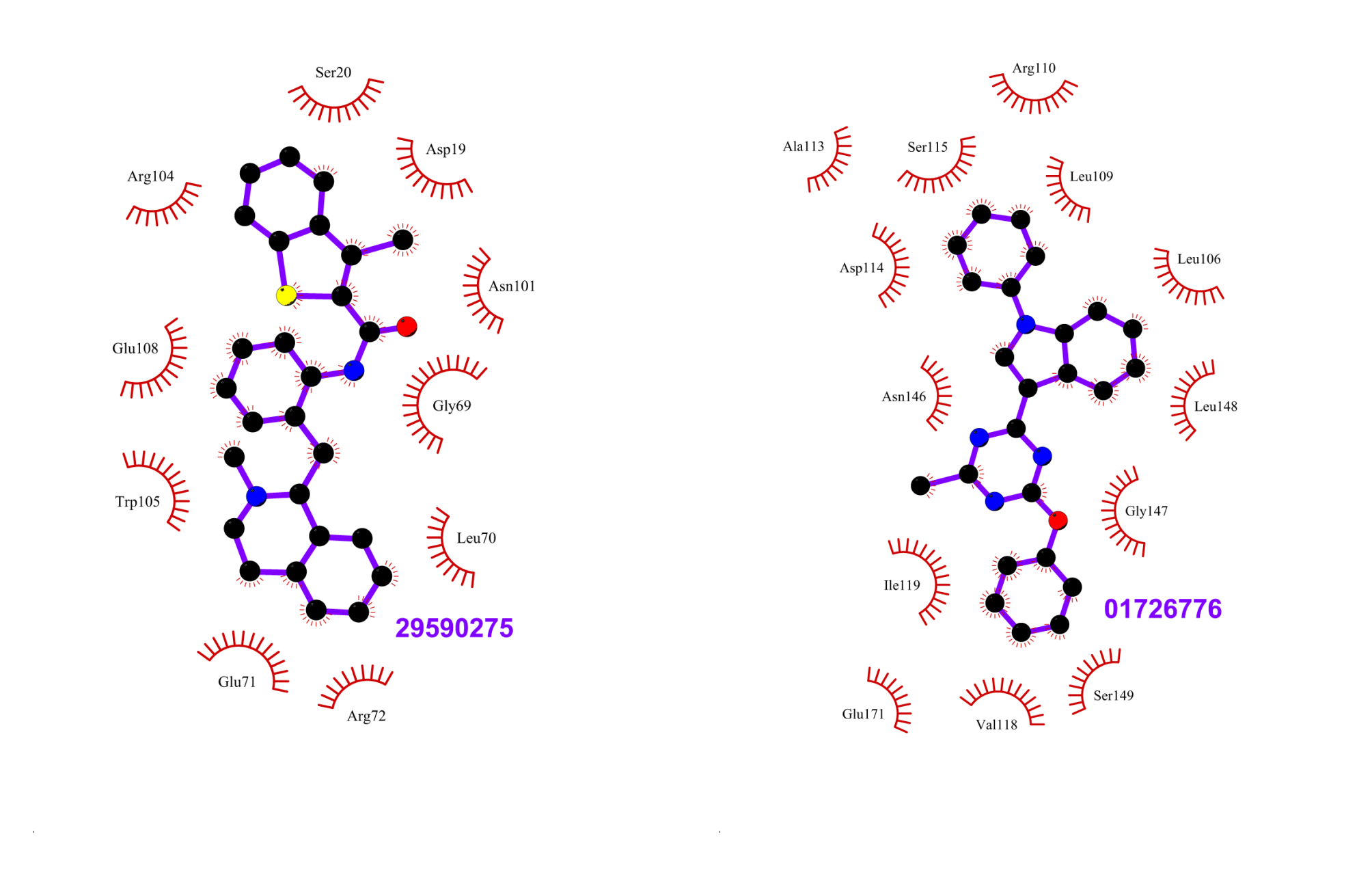

Supplement: S37 Fig — The figure shows the interactions of the ligands (labeled) with the residues of the Rab11 structures in which they scored the best. The ligand and Rab11 side chains are shown in ball-and-stick representation. Black circles denote carbon atoms, red circles denote oxygen atoms, yellow circle denote sulfur atom and blue circles denote nitrogen atoms. The ligand bonds are colored in purple. Residues in Rab11 interacting with the ligand are labeled. Hydrogen bonds are shown as green dotted lines. The Rab11 residues making nonbonded contacts with the ligand are shown as spoked arcs. Figures are generated using LigPlot+ [66][67]. (TIF) [file pone.0198632.s037.tif]

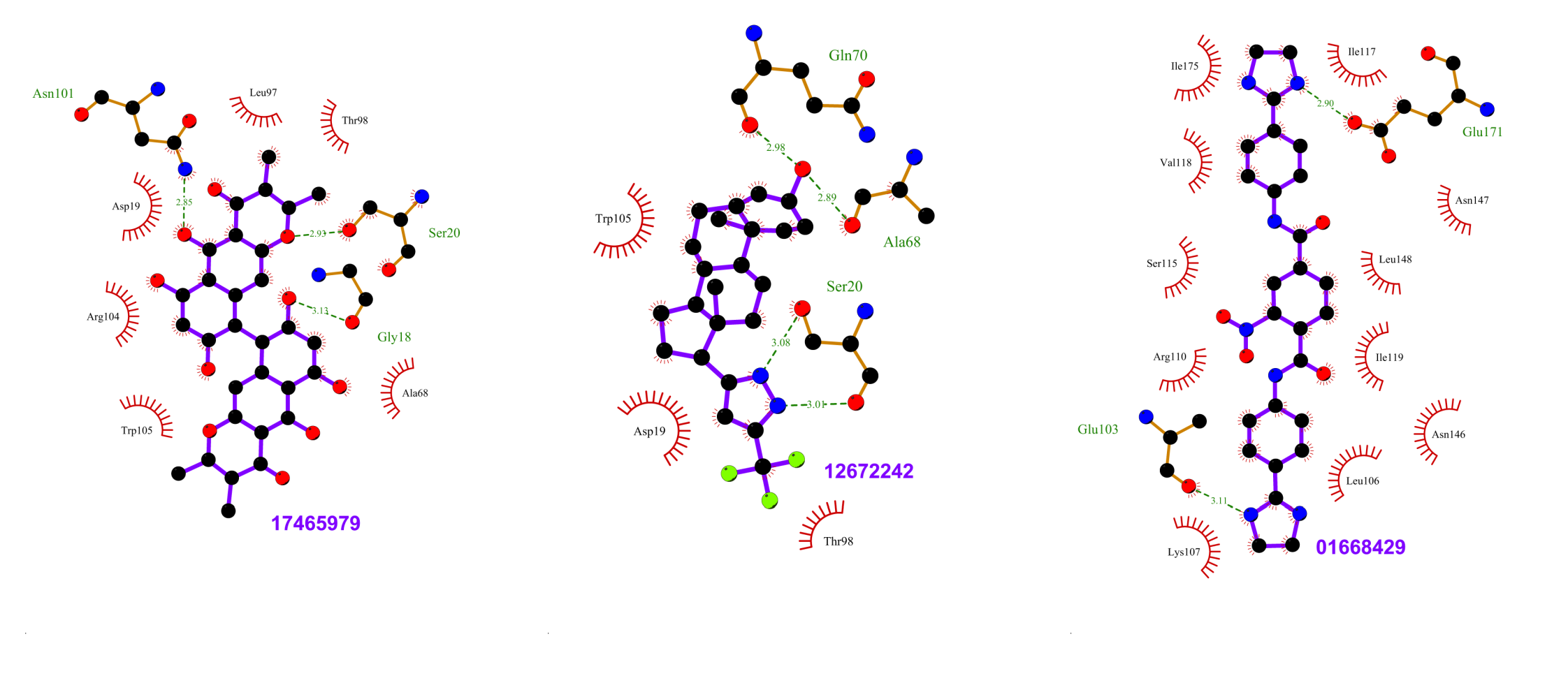

Supplement: S38 Fig — The figure shows the interactions of the structurally diverse ligands (labeled) that prefer PDB entry 4OJK_A and their interactions. ZINC17465979, ZINC17465983, ZINC05462674 and ZINC05462670 are isomers. The ligand and Rab11 side chains are shown in ball-and-stick representation. Black circles denote carbon atoms, red circles denote oxygen atoms, green circles denote fluorine atoms and blue circles denote nitrogen atoms. The ligand bonds are colored in purple. Residues in Rab11 interacting with the ligand are labeled. Hydrogen bonds are shown as green dotted lines. The Rab11 residues making nonbonded contacts with the ligand are shown as spoked arcs. Figures are generated using LigPlot+ [66][67]. (TIF) [file pone.0198632.s038.tif]

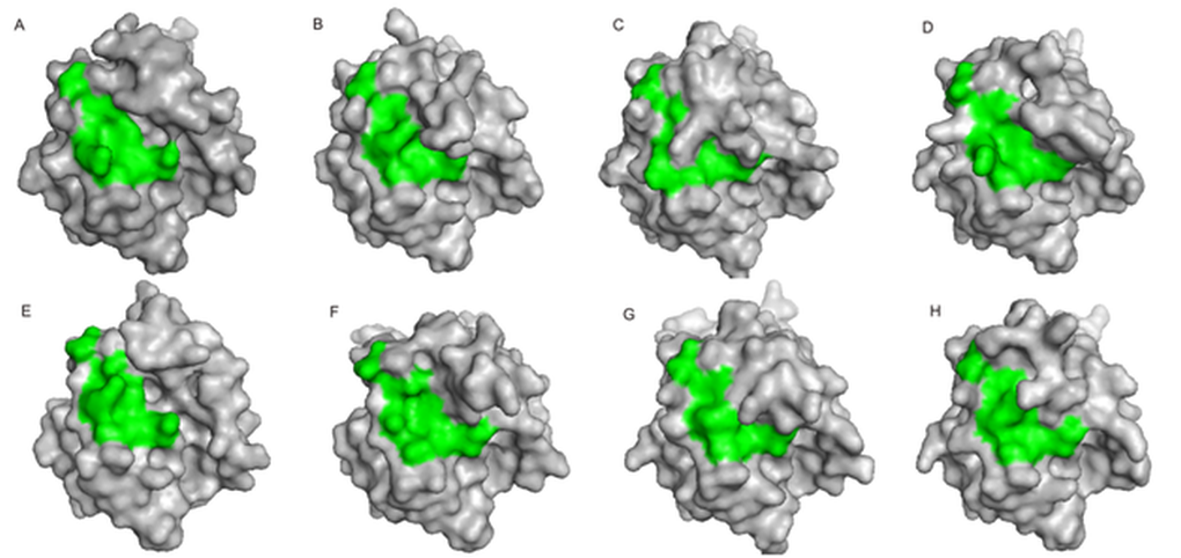

Supplement: S39 Fig — Surface representations of PDB entries 1OIV_A, 1YZK_A, 4C4P_A, 4LX0_C, 4OJK_A, 4UJ5_B, 5C46_F, and 5JCZ_D are shown in (A), (B), (C), (D), (E), (F), (G) and (H), respectively. Residues 19, 20, 101, 104, 105, 108 and 111 forming site 1 are colored in green. (TIF) [file pone.0198632.s039.tif]

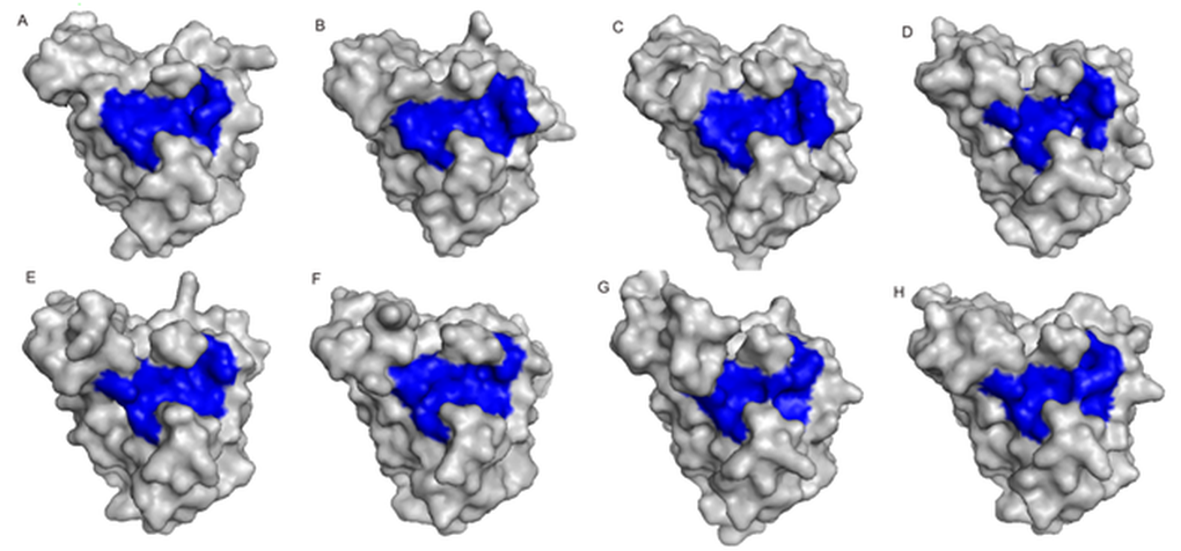

Supplement: S40 Fig — Surface representations of PDB entries 1OIV_A, 1YZK_A, 4C4P_A, 4LX0_C, 4OJK_A, 4UJ5_B, 5C46_F and 5JCZ_D are shown in (A), (B), (C), (D), (E), (F), (G) and (H), respectively. Residues 106, 109, 110, 113, 117, 118, 119, 148, 149 and 171 forming site 2 are colored in blue. (TIF) [file pone.0198632.s040.tif]

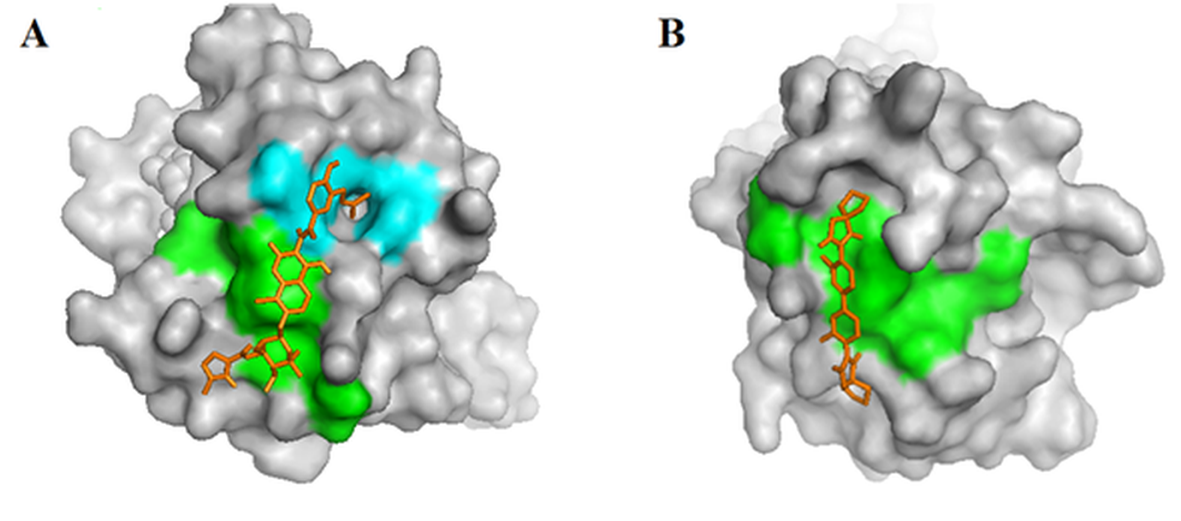

Supplement: S41 Fig — Ligands docked at PDB entries (A) 5C46_F and (B) 5JCZ_D. Ligands (A) ZINC29590257 and (B) ZINC01568793 are shown in brown sticks. Residues of site 1 are colored in green and ligands are shown as brown sticks. In 5C46_F, ligands interact with a small cavity formed by residues H99, L100 and A142 which are colored in cyan. (TIF) [file pone.0198632.s041.tif]

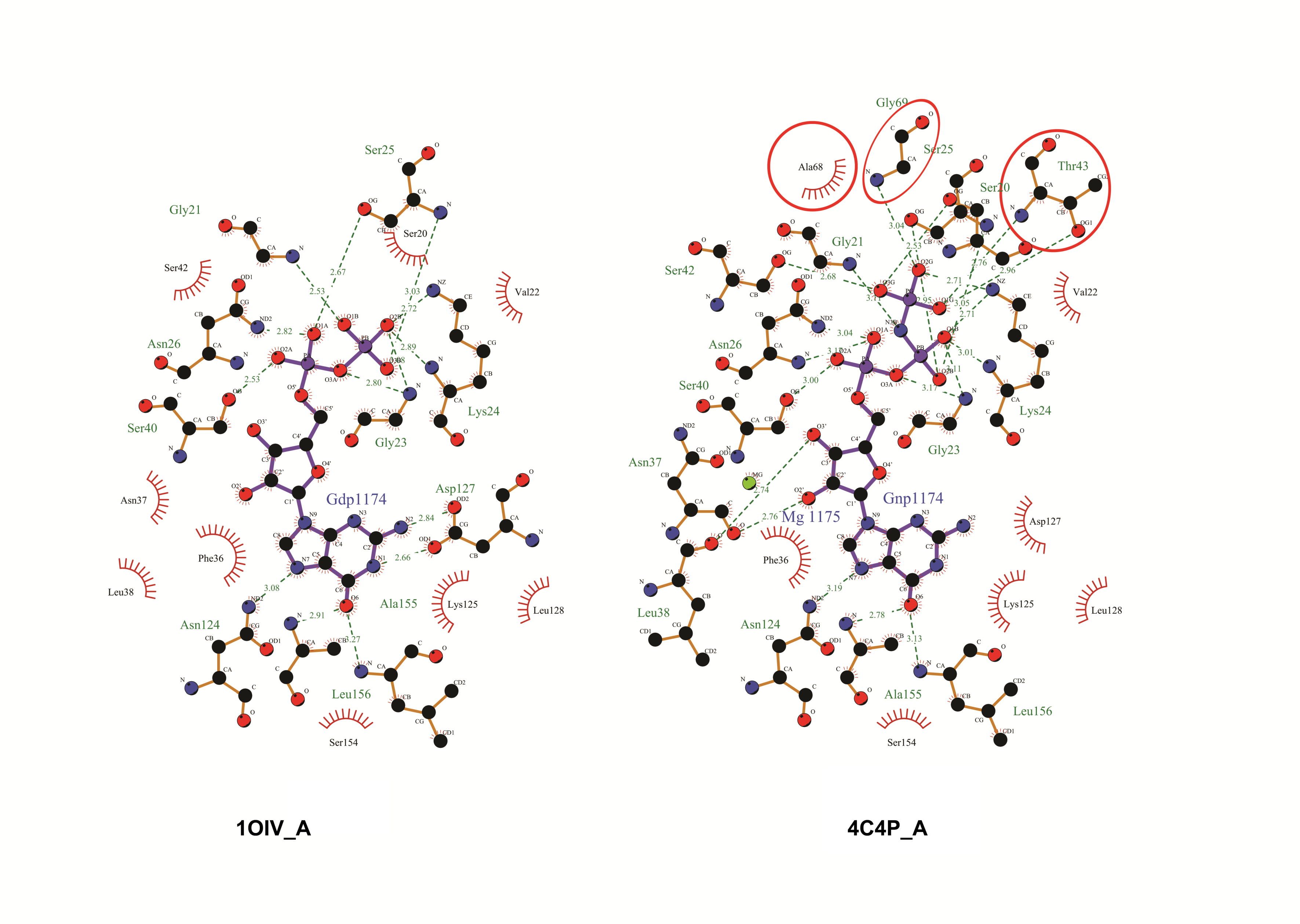

Supplement: S42 Fig — The ligand and Rab11 side chains are shown in ball-and-stick representation. The ligand bonds are colored in purple. Hydrogen bonds are shown as green dotted lines. The Rab11 residues making nonbonded contacts with the ligand are shown as spoked arcs. The red circle and ellipses indicate protein residues that are in non-equivalent positions when the two structures are superposed. The figure shows that compared to 1OIV_A, there are two extra Rab11 residues (Thr43 from switch 1, and GLY69 from switch 2) that have hydrogen bonds with the ligand. The Figures are generated using LigPlot+ [66][67]. (TIF) [file pone.0198632.s042.tif]

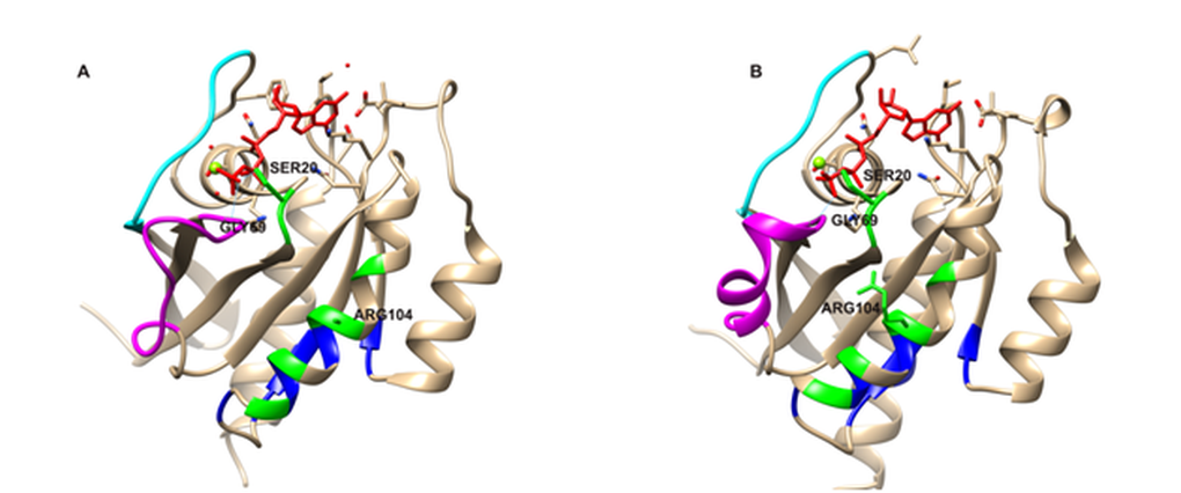

Supplement: S43 Fig — Conformation of switch regions in (A) PDB entry 4UJ5_B and (B) PDB entry 5C46_F. Switch 1 and switch 2 of the PDB entries are colored in cyan and magenta, respectively. The ligands at the active sites of the structures are colored in red. The magnesium ion at the active site of the structures is shown as green spheres. Water molecules are shown as red spheres. The site 1 and site 2 residues are colored in green and blue, respectively. Residues GLY69 (magenta; from switch 1 region), SER20 (green; from site 1) and ARG104 (green, from site 1) are labeled in both structures. GLY69 and SER20 have hydrogen bond interactions with the ligand at the active site. The switch 2 region and ARG104 adopt different conformations in them. Figures are generated using UCSF Chimera. (TIF) [file pone.0198632.s043.tif]

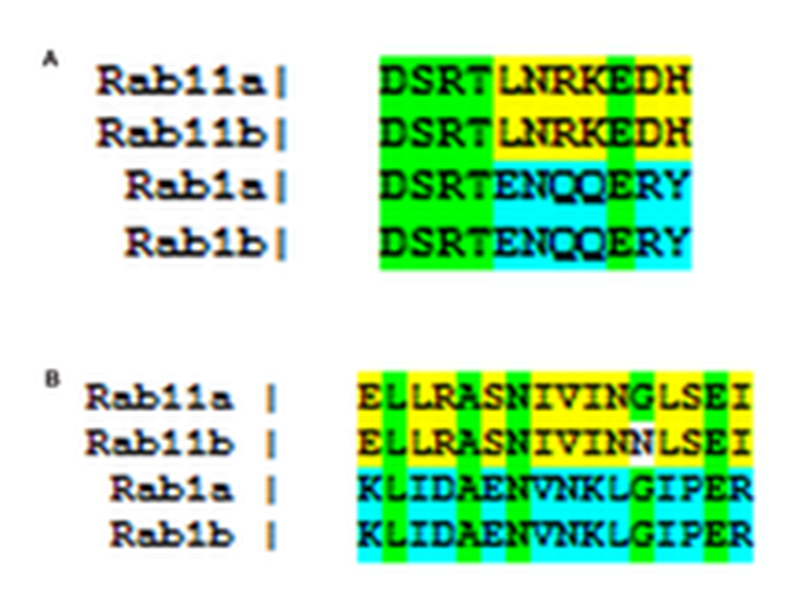

Supplement: S44 Fig — Multiple sequence alignment of (A) site 1 and (B) site 2 in Rab1 and Rab11. Residues conserved in three or more sequences are highlighted in green. Residues conserved only in Rab11 are highlighted in yellow. Residues conserved only in Rab1 are highlighted in cyan. (TIF) [file pone.0198632.s044.tif]

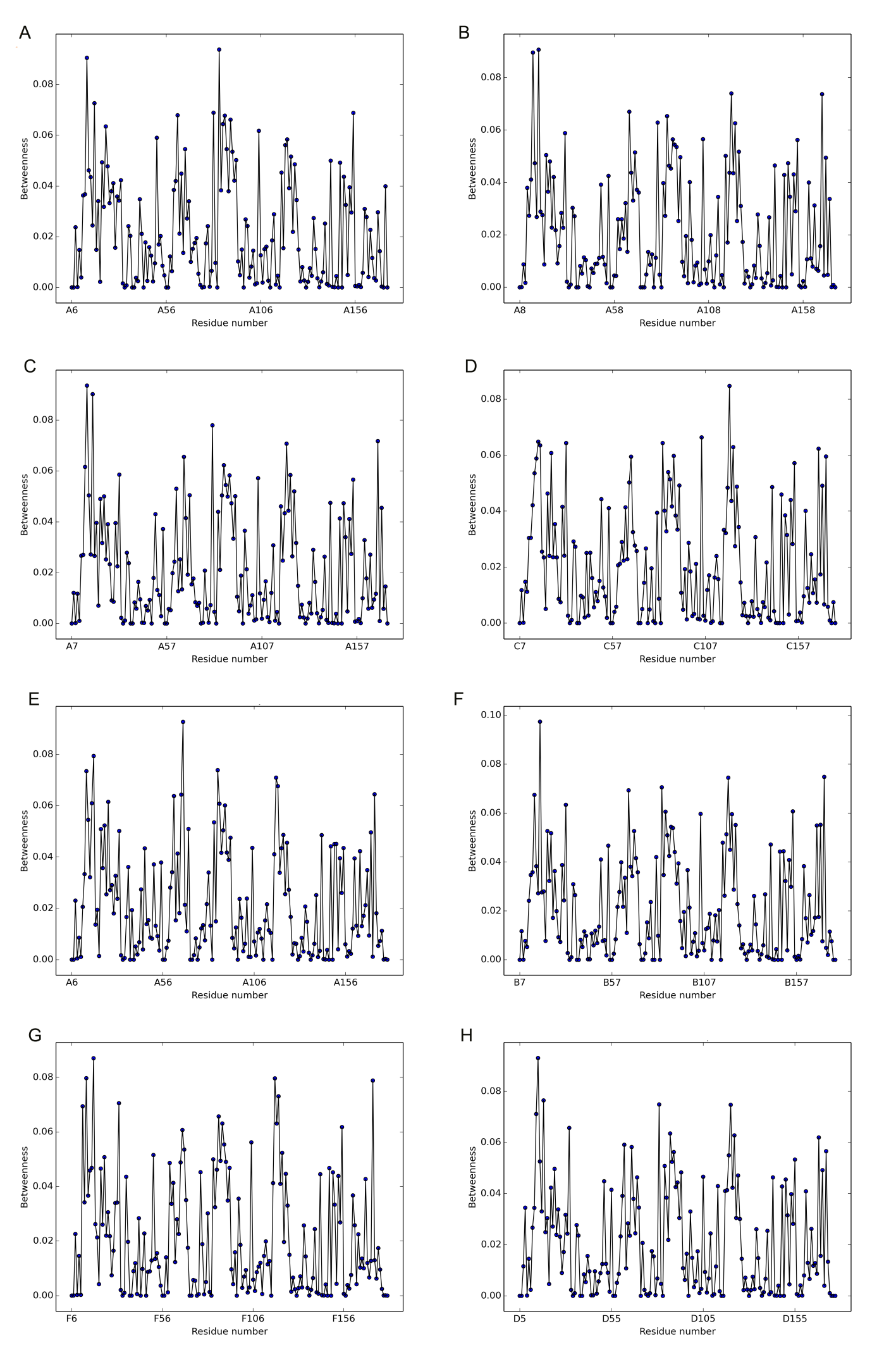

Supplement: S45 Fig — Plots generated by NAPS server showing betweenness centrality values of residues in (A) 1OIV_A, (B) 1YZK_A, (C) 4C4P_A, (D) 4LX0_C, (E) 4OJK_A, (F) 4UJ5_B, (G) 5C46_F and (H) 5JCZ_D. X axis shows residue number and Y axis shows betweenness centrality values of residues. (TIF) [file pone.0198632.s045.tif]

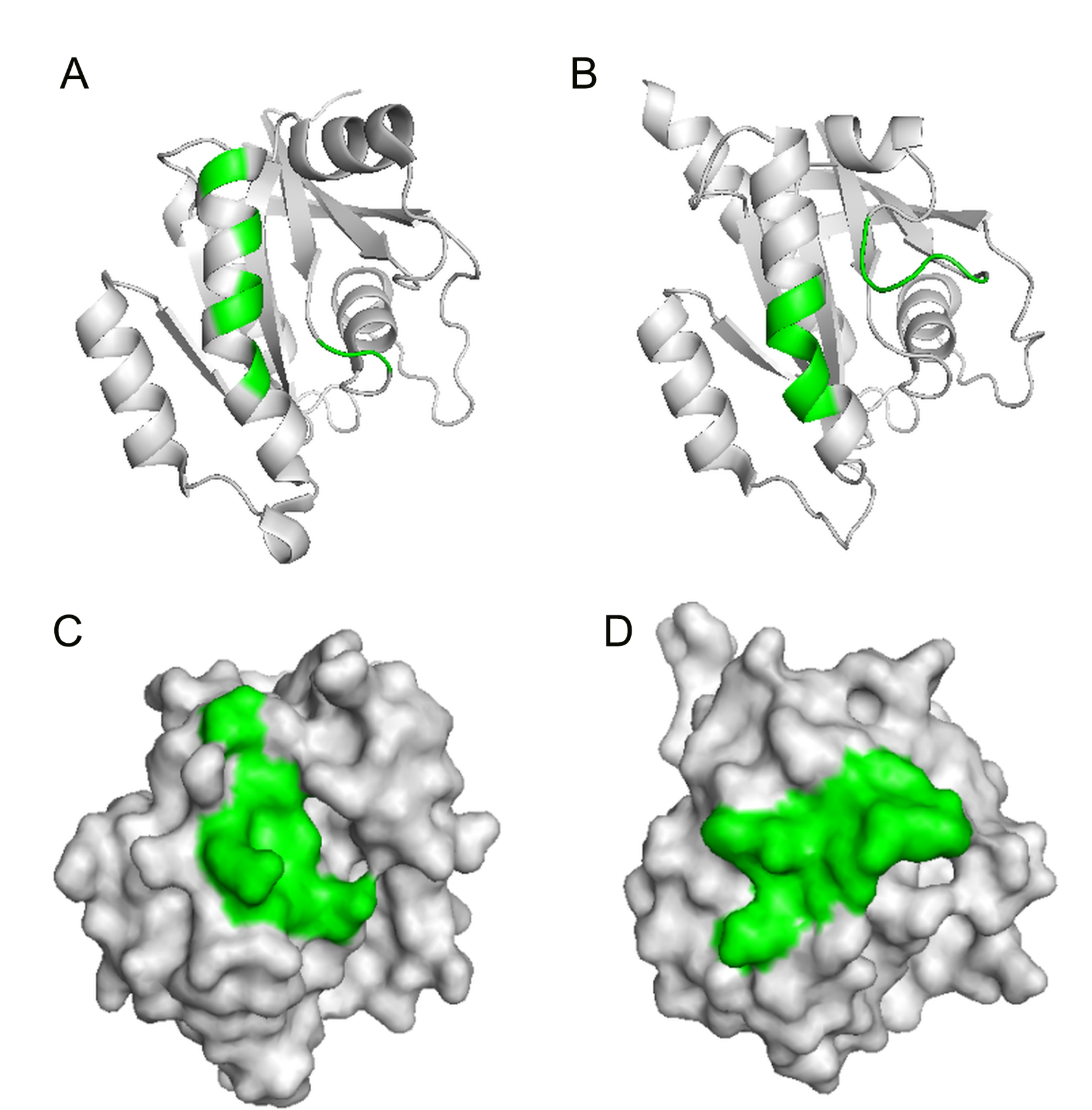

Supplement: S46 Fig — The figure shows Rab11 (PDB entry 1OIV_A) and H-Ras (PDB entry 2Q21_A) structures aligned in the same orientation. (A) and (B) show site 1 (colored in green) in cartoon representations of Rab11 and H-Ras. (C) and (D) show site 1 (colored in green) in surface representations of Rab11 and H-ras. Residues 19, 20, 101, 104, 105, 108 and 111 of PDB entry 1OIV_A are colored in green. Residues 61–65 and 90–95 of PDB entry 2Q21_A are colored in green. (TIF) [file pone.0198632.s046.tif]

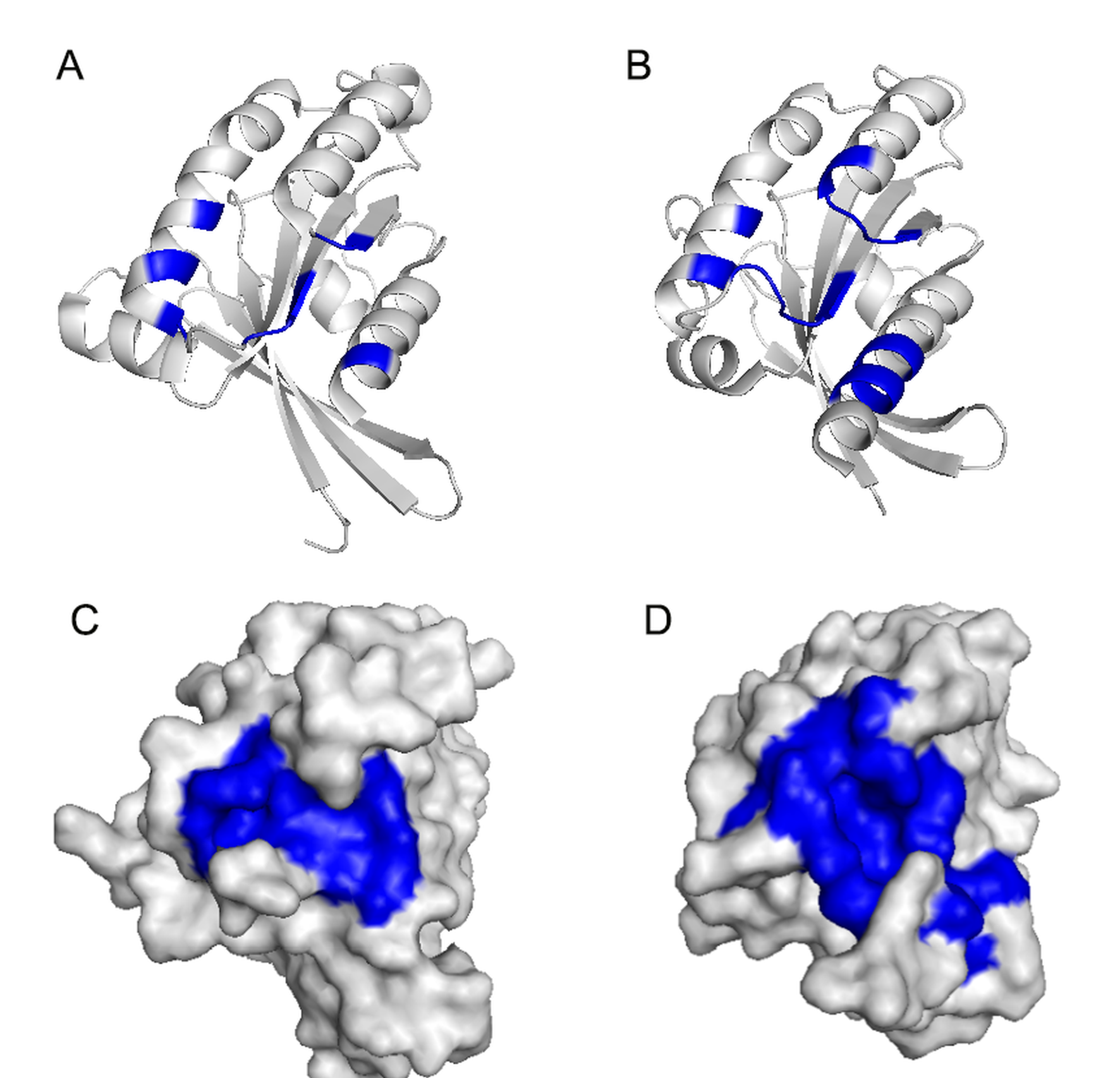

Supplement: S47 Fig — The figure shows Rab11 (PDB entry 1OIV_A) and H-Ras (PDB entry 2Q21_A) structures aligned in the same orientation. (A) and (B) show site 2 (colored in blue) in cartoon representations of Rab11 and H-Ras. (C) and (D) show site 2 (colored in blue) in surface representations of Rab11 and H-Ras. Residues 110, 117–119, 148, 149 and 171 of PDB entry 1OIV_A are colored in blue. Residues 97, 101, 107–111, 136–140 and 161–166 of PDB entry 2Q21_A are colored in blue. (TIF) [file pone.0198632.s047.tif]

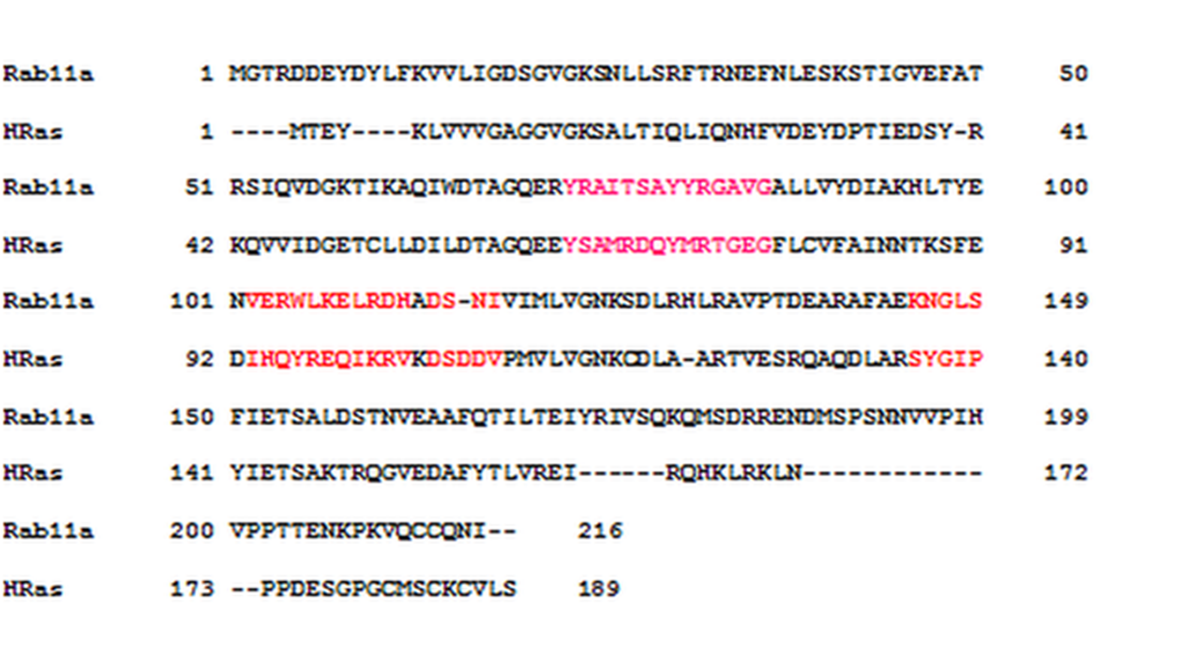

Supplement: S48 Fig — Helix α2 (residues Y64-G77) in H-Ras and the corresponding amino acid sequences in the PSA are indicated in magenta color. Residues of helix α3 (I93-V103) and residues D105-V109 and residues S136-P140 that line pocket p3 in H-Ras and the corresponding residues in the PSA that line sites 1 and 2 in Rab11 are indicated in red. (TIF) [file pone.0198632.s048.tif]

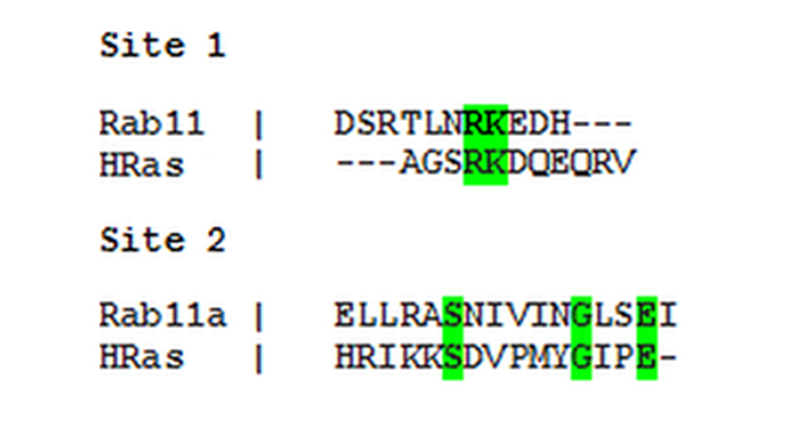

Supplement: S49 Fig — The figure shows the sequence alignment of site 1 and site 2 residues that are contacted by small molecules during virtual screening, and the corresponding residues in H-Ras pockets p2 and p3, respectively. The residues conserved between the binding sites are highlighted in green shade. (TIF) [file pone.0198632.s049.tif]

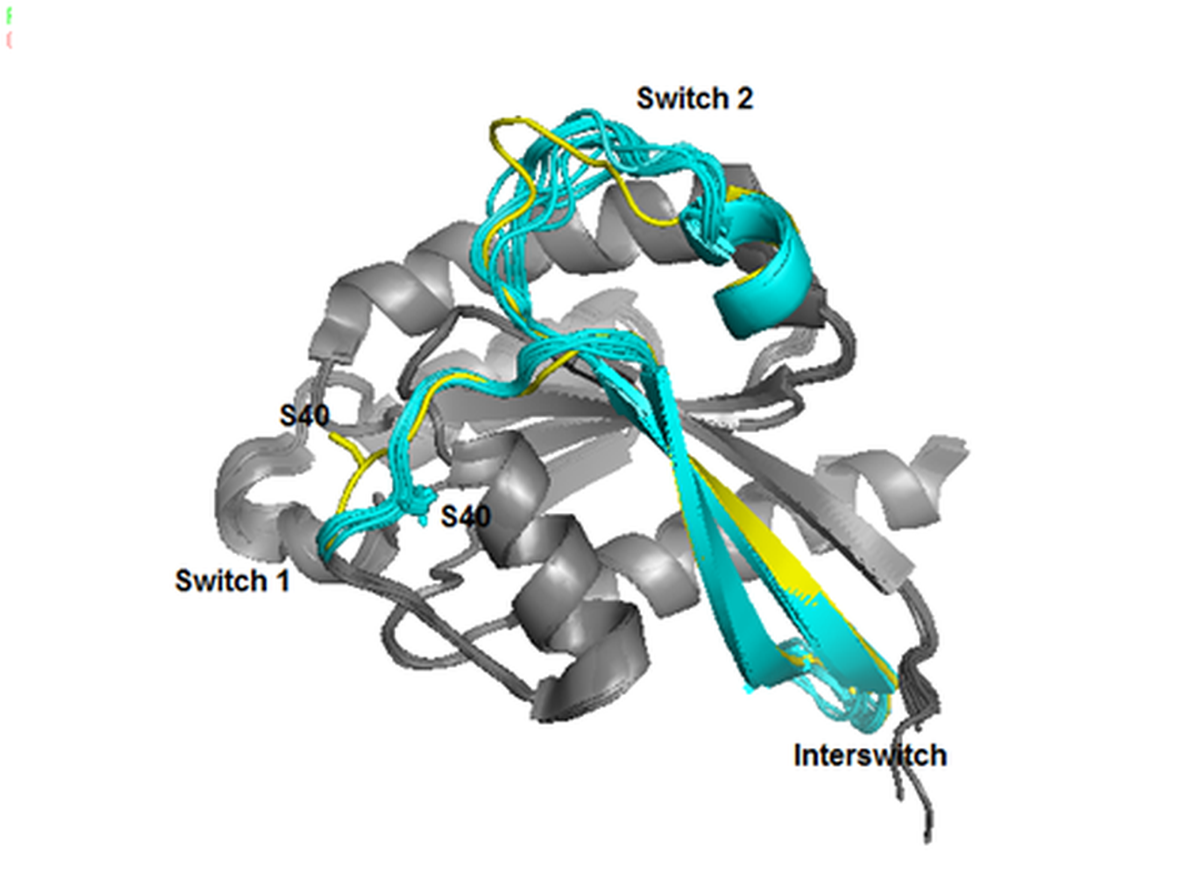

Supplement: S50 Fig — Residue S40 that adopts different orientations in the structures is labeled. Switch 1 region of these Rab11a-FIP complexes are similar and switch 2 adopts different conformations. (TIF) [file pone.0198632.s050.tif]

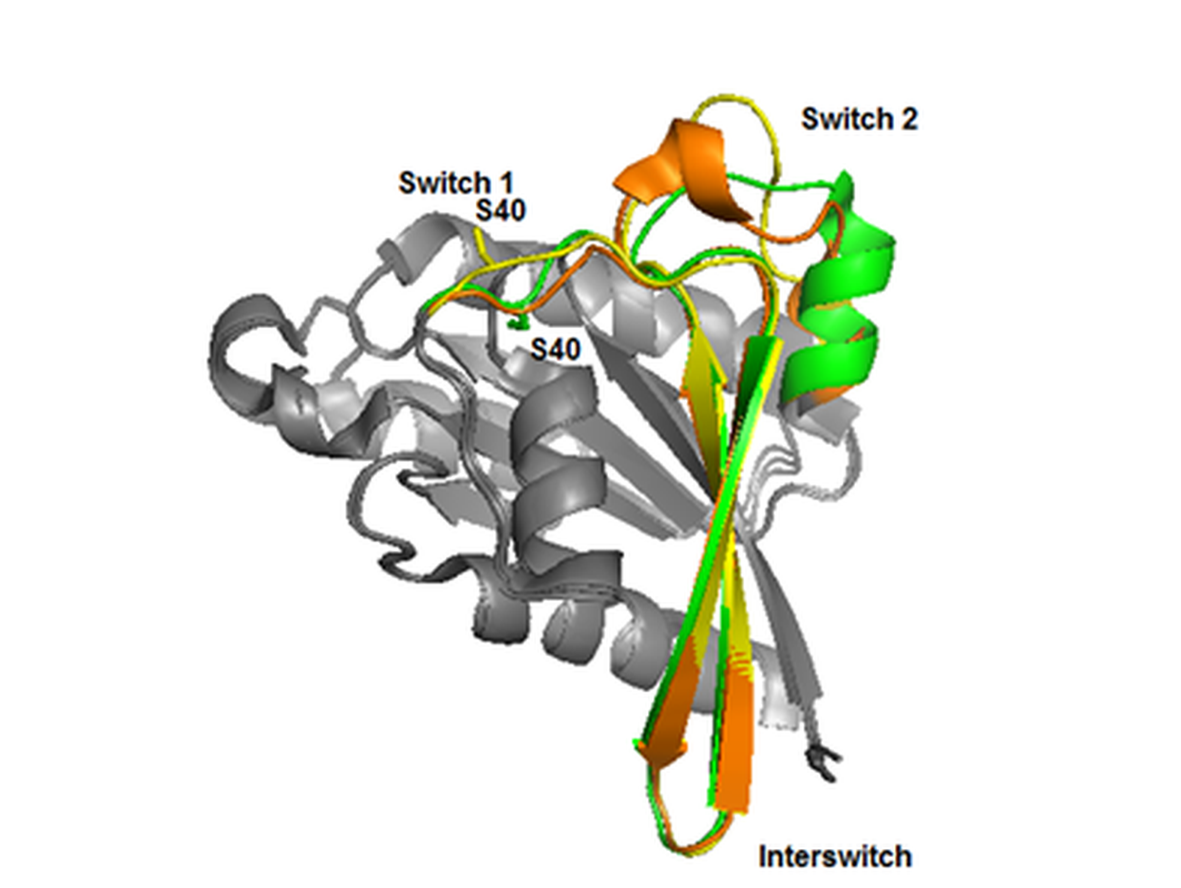

Supplement: S51 Fig — Residue S40 adopts different orientations in the structures. Switch 1 and switch 2 adopt different conformations from those of unbound-Rab11a. (TIF) [file pone.0198632.s051.tif]

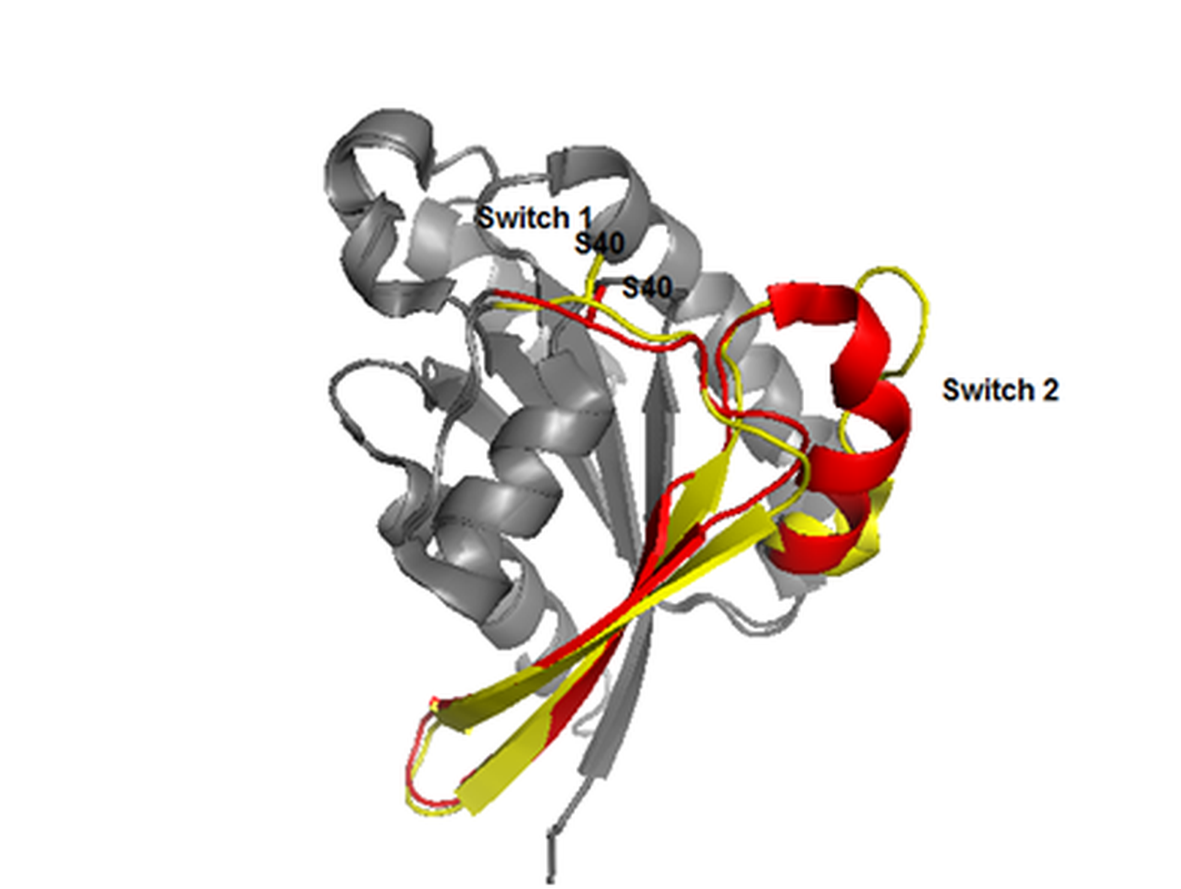

Supplement: S52 Fig — P14KB induces conformational changes in the switch regions of Rab11a. Unlike the observations on residue S40 in other protein complexes used in our study, residue S40 adopts a conformation similar to that in unbound-Rab11a-GTP. (TIF) [file pone.0198632.s052.tif]

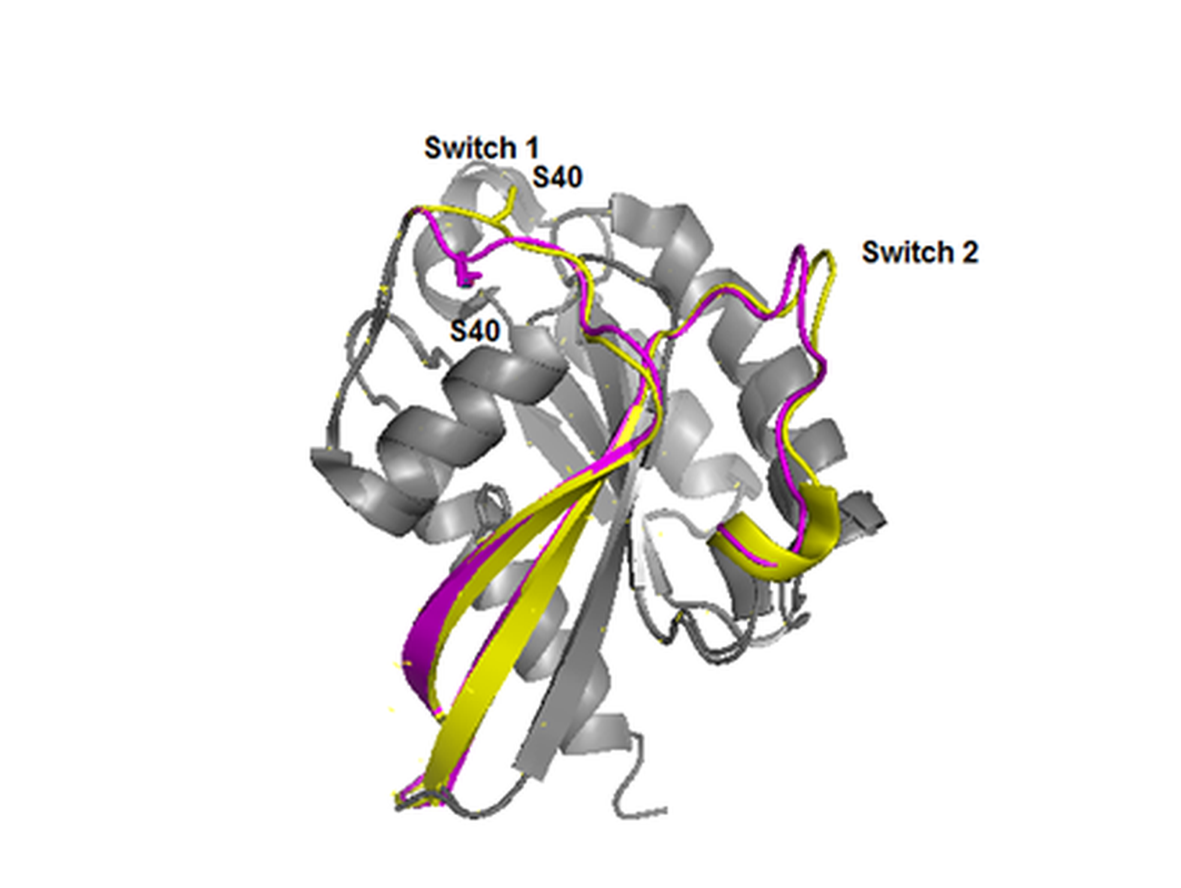

Supplement: S53 Fig — Rabin8 induces conformational changes in the switch regions of Rab11a. (TIF) [file pone.0198632.s053.tif]
