# Supplementary material for: Allosteric binding sites in Rab11 for potential drug candidates
Source: PLoS One. 2018 Jun 6;13(6):e0198632. doi: 10.1371/journal.pone.0198632 (PMC5991966; doi:10.1371/journal.pone.0198632)
Supplement: S1 Table — For each Rab11 structure in the ensemble, the experimental technique used for determining the structure, its resolution and its ligands are listed. GSP stands for 5'-Guanosine-Diphosphate-Monothiophosphate, MG stands for Magnesium ion, GNP stands for Phosphoaminophosphonic acid-guanylate ester, SO4 stands for sulfate ion, 2ME stands for Methoxyethane, MES stands for 2-(N-morpholino)-ethanesulfonic acid, GTP stands for Guanosine-5'-Triphosphate, GDP stands for Guanosine-5'-Diphosphate, CL stands for Chloride ion, PO4 stands for phosphate ion, GOL stands for glycerol, BEF stands for Beryllium trifluoride ion, EDO stands for Ethylene glycol, ACT stands for Acetate ion, MSE stands for Selenomethionine and NI stands for Nickel (ii) ion. (DOCX) [file pone.0198632.s054.docx]

| **PDB ID** | **Experimental Technique** | **Resolution** | **Ligand Id** |
| --- | --- | --- | --- |
| 4D0L_B | X-RAY DIFFRACTION | 2.94 | GSP,MG |
| 4D0L_D | X-RAY DIFFRACTION | 2.94 | GSP,MG |
| 4D0L_F | X-RAY DIFFRACTION | 2.94 | GSP,MG |
| 5C46_F | X-RAY DIFFRACTION | 2.65 | GSP,MG |
| 4UJ5_B | X-RAY DIFFRACTION | 2.6 | GNP,MG |
| 4UJ3_A | X-RAY DIFFRACTION | 3 | GNP,MG |
| 4UJ3_G | X-RAY DIFFRACTION | 3 | GNP,MG |
| 2HV8_A | X-RAY DIFFRACTION | 1.86 | GTP,MG,SO4 |
| 2HV8_B | X-RAY DIFFRACTION | 1.86 | 2ME,GTP,MES,MG,SO4 |
| 2HV8_C | X-RAY DIFFRACTION | 1.86 | GTP,MG,SO4 |
| 1OIW_A | X-RAY DIFFRACTION | 2.05 | GSP,MG |
| 1OIX_A | X-RAY DIFFRACTION | 1.7 | CL,GDP,MG,PO4 |
| 4LX0_A | X-RAY DIFFRACTION | 2.19 | BEF,GDP,GOL,MG |
| 4LX0_C | X-RAY DIFFRACTION | 2.19 | BEF,GDP,MG |
| 5JCZ_A | X-RAY DIFFRACTION | 2.06 | ACT,BEF,EDO,GDP,GOL,MG |
| 5JCZ_D | X-RAY DIFFRACTION | 2.06 | BEF,EDO,GDP,MG |
| 1OIV_A | X-RAY DIFFRACTION | 1.98 | GDP,SO4 |
| 1OIV_B | X-RAY DIFFRACTION | 1.98 | EDO,GDP |
| 2GZD_A | X-RAY DIFFRACTION | 2.44 | GTP,MG,MSE |
| 2GZD_B | X-RAY DIFFRACTION | 2.44 | GTP,MG,MSE |
| 2GZH_A | X-RAY DIFFRACTION | 2.47 | GTP,MG,MSE,PO4 |
| 4C4P_A | X-RAY DIFFRACTION | 2 | GNP,MG |
| 2D7C_A | X-RAY DIFFRACTION | 1.75 | GTP,MES,MG,MSE |
| 2D7C_B | X-RAY DIFFRACTION | 1.75 | GTP,MG,MSE |
| 1YZK_A | X-RAY DIFFRACTION | 2 | GNP,MG |
| 4OJK_A | X-RAY DIFFRACTION | 2.66 | GDP |
| 2F9M_A | X-RAY DIFFRACTION | 1.95 | GNP,MG,NI |
