# Supplementary material for: Allosteric binding sites in Rab11 for potential drug candidates
Source: PLoS One. 2018 Jun 6;13(6):e0198632. doi: 10.1371/journal.pone.0198632 (PMC5991966; doi:10.1371/journal.pone.0198632)
Supplement: S2 Table — Residues in Rab11 that are not conserved in Rab1 are highlighted in cyan. (DOCX) [file pone.0198632.s055.docx]

| **Rab11 structures** | **Interacting partners** | **Binding sites** |
| --- | --- | --- |
| 4DOL,5C46 (Rab11a) | P14KB | F36, L38, E39, S40, L128, H130, L131 |
| 4UJ3, 4UJ5 (Rab11a) | Rabin8 | L38, E39, H130, L131 |
| 4UJ3, 4UJ5 (Rab11a) | FIP3 | S42, E47, I44, Y80, R82 |
| 2HV8 (Rab11a) | FIP3 | R33, K41, S42, I44, G45, V46, E47, F48, A49, T50, W65, R72, R74, I76, Y80, R82 |
| 5JCZ (Rab11a) | Myosin 5a | T32, R33, E35, I44, G45, V46, E47, F48, A49, T50, K61, Q63, W65, Q70, R72, Y73, A75, I76, A79, R82 |
| 4LX0 (Rab11a) | Myosin 5b | K13, R33, I44, V46, F48, T50, Q63, W65, E71, Y73, A75, I76, T77, Y80 |
| 4C4P,2GZD,2GZH (Rab11a) | FIP2 | R33, K41, G45, V46, E47, F48, A49, T50, K58, W65, R72, R74, A75, I76, A79, R82 |
| 2D7C (Rab11a) | FIP3 | I44, G45, V46, W65, R72, I76, A79, Y80 |
| 4OJK (Rab11b) | PKGII | D9,Y10,L11,F12, K41, F48, W65, Y73, I76, V85 |
