# Supplementary material for: Allosteric binding sites in Rab11 for potential drug candidates
Source: PLoS One. 2018 Jun 6;13(6):e0198632. doi: 10.1371/journal.pone.0198632 (PMC5991966; doi:10.1371/journal.pone.0198632)
Supplement: S3 Table — Residues in Rab1 that are not conserved in Rab11 are highlighted in cyan. (DOCX) [file pone.0198632.s056.docx]

| **Rab1 structures** | **Interacting partners** | **Binding sites** |
| --- | --- | --- |
| 3L0I (Rab1a) | GEF/GDF domain of sidM/DrrA from *Legionella pneumophila* | Y36, E38, S39, Y40, I41, S42, T43, Q63, W65, Q70, E71 |
| 3SFV (Rab1a) | LidA from *Legionella pneumophila* | Y8, G21, Y40, I44, V46, F48, K61,W65,Q70, F73, Y80, R82 |
| 3TKL (Rab1a) GTP | LidA from *Legionella pneumophila* | Y8, G18, S20, G21, V22, G23, K24, S25, C26, L27, Y36, T37, E38, S39, Y40, I41, S42, T43, K61, Q63, W65, T67, A68, G69, Q70, E71, R72, F73, R74, T75,S79, R82 |
| 4FMB (Rab1a) | VirA from *Shigella flexneri* (acts as GAP for Rab1) | S20, G21, L29, D33, E38, S39, Y40, I41, S42,T43, I44, G45, V46, D47, F48, K49, I50, K61, Q63, W65, A68, G69, Q70, E71, R72, F73, T75, I76, Y80, E97, K125, L128, K131 |
| 4FMC, 4FMD, 4FME (Rab1a) | ESPG from *Escherichia coli*  (acts as GAP for Rab1) | S20, G21, E38, S39, Y40, I41, S42,T43, I44, G45, V46, D47,F48, K49, I50, K61, Q63, W65, A68, G69, Q70,E71,R72,F73, I76, Y80, D92, T94, K125, L128 |
| 4IRU (Rab1a) | LepB from *Legionella pneumophila* (acts as GAP for Rab1) | K13, Y40, S42, I44, V46, D47, F48, E71, R72, F73, I76, Y80, R82 |
| 4JVS (Rab1a) | LepB from *Legionella drancourtii*  (acts as GAP for Rab1) | S39, Y40, I41, S42, T43, I44, G45,V46, D47, F48, K49, I50, Q63, W65, Q70, E71, R72, F73, I76, Y80 |
| 3JZA (Rab1b) | GEF/GDF domain of sidM/DrrA from *Legionella pneumophila* | Y5, Y7, L8, K10, K21, S22, D31, T32, Y33,T34,E35,S36,Y37,I38, T40, I41, G42, V43, D44, K58, Q60, W62, D63,T64, A65, E68, R69, F70, T72, Y77 |
| 4HLQ (Rab1b) | TBC1D20 (GAP protein) | S17, G18, T34, S36,Y37,I38, T40,I41,G42,V43,D44, F45, Q60, W62, A65, G66, Q67, R69, I73, Y77, E94 |
| 4I1O (Rab1b) | LepB from *Legionella pneumophila* (acts as GAP for Rab1) | Y37, I38, I41, ,V43,D44, F45, I47, Q70, E68, R69, F70 |
