# Supplementary material for: Allosteric binding sites in Rab11 for potential drug candidates
Source: PLoS One. 2018 Jun 6;13(6):e0198632. doi: 10.1371/journal.pone.0198632 (PMC5991966; doi:10.1371/journal.pone.0198632)
Supplement: S4 Table — Residues of switches 1 and 2 (residues 39–46 and 68–79) are shown in bold. RabF and RabSF regions are highlighted in magenta and orange, respectively. (DOCX) [file pone.0198632.s057.docx]

| **Representative structures** | **Residues forming binding sites via hydrophobic interactions** | **Residues forming binding sites via electrostatic interactions** | **References** | **DOI of references** |
| --- | --- | --- | --- | --- |
| 1OIV_A | 16, 19, 20, 21, 22, 23, 25, 26, 28, 29, 36, 67, **72**, **76**, **77**, 80, 81, 99, 101, 102, 103, 104, 105, 108, 116, 117, 118, 119, 140, 142, 150, 151 | 32, 67, 81, 104,108, 117, 119, 124, 147, 152 | Pasqualato *et al.* J.Biol.Chem. (2004) | 10.1074/jbc.M310558200 |
| 1YZK_A | 10, 12, 19, 20, 21, 22, 23, 24, 25, 26, **40**, **43**, **45**, 67, **68**, **69**, **70**, **71**, **74**, **75**, **76**,  99, 100, 102, 104, 106, 109, 110, 115, 116, 117, 118, 119 | 23, 24, 25, 26, 40, 67, **68**, 110, 117, 119 | Eathiraj *et al.* Nature. (2005) | 10.1038/nature03798 |
| 4C4P_A | 10, 13, 19, 20, 21, 22, 23, 24, 25, 26, 36, 37, 38, **40**, **41**, **42**, **43**, 67, **68**, **69**, **70**, **71**, **72**, **77**, 80, 81, 99, 100, 101, 102, 103, 105, 106,  109, 110, 115, 117, 118, 119, 147, 148, 150, 151 | 23, 24, 25, 26, **40**, **69**, **72**, 110, 117, 119, 146, 147, 150, 152 | Lall *et al.* Biochim.Biophys.Acta. (2013) | 10.1016/j.bbapap.2013.09.005 |
| 4LX0_C | 10, 11, 19, 20, 21, 22, 23, 24, 25, 26, 36, **43**, 67, **69**, **74**, 101, 104, 105, 109, 110, 113, 114, 115, 116, 117, 118, 119 | 11, 19, 20, 23, 24, 25, 26, **43**, **69**, **71**, 117, 119, 147, 149 | Pylypenko *et al.* Proc.Natl.Acad.Sci.USA. (2013) | 10.1073/pnas.1314329110 |
| 4OJK_A | 10, 11, 12, 13, 18, 19, 20, 24, 25, 26, 28, **41**, **42**, **43**, **44**, **45**, **46**, 47, 66, **68**, 106, 110, 115, 116, 117, 118, 119, 147 | 11, 13, 18, 24, 25, 26, **41**, 47, 110, 117, 119, 146, 147 | Reger *et al.* J.Biol.Chem. (2014) | 10.1074/jbc.M114.575894 |
| 4UJ5_B | 10, 11, 19, 20, 21, 22, 23, 24, 25, 26, 28, **40**, **43**, **45**, 66, 67, **68**, **69**, **70**, **71**, **74**, **75**, **76**, 110,116, 117, 118, 119, 147, 148, 171 | 11, 13, 21, 22, 23, 24, 25, 26, **40**, **43**, 117, 119, 171 | Vetter *et al.* Nat.Struct.Mol.Biol. (2015) | 10.1038/nsmb.3065 |
| 5C46_F | 10, 11, 12, 19, 20, 21, 22, 23, 24, 25, 26, **43**, 85, 99, 110, 116, 117, 118, 119 | 11, 20, 24, 25, 117, 119, 149, 171 | Fowler *et al.* Protein Sci. (2016) | 10.1002/pro.2879 |
| 5JCZ_D | 10, 11, 12, 13, 19, 20, 21, 22, 23, 24, 25, 26, 36, 37, 38, **40**, **41**, **42**, **43**, **46**, 71, **72**, 105, 110, 115, 116, 117, 118, 119, 120, 125, 147, 148, 149, 150, 151, 167, 171 | 11, 13, 19, 21, 23, 24, 25, 26, 37, 38, **40**, **43**, 69, **72**, **77**, 104, 110, 117, 119, 124, 147, 150 | Pylypenko *et al.* Elife. (2016) | 10.7554/eLife.17523 |
