# Supplementary material for: Allosteric binding sites in Rab11 for potential drug candidates
Source: PLoS One. 2018 Jun 6;13(6):e0198632. doi: 10.1371/journal.pone.0198632 (PMC5991966; doi:10.1371/journal.pone.0198632)
Supplement: S5 Table — The table lists the free energy values computed by Vinardo for the top scoring ligands when docked at different sites in Rab11 representative structures. (DOCX) [file pone.0198632.s058.docx]

| **Ligand** | **Target structure** | **Site** | **Free energy (Kcal/mol)** |
| --- | --- | --- | --- |
| ZINC29590259 | 4LX0_C | Site 1 | -10.5 |
|  | 5C46_F | Site 1 | -9.8 |
|  | 1OIV_A | Site 1 | -10.4 |
|  | 4OJK_A | Site 2 | -8.7 |
| ZINC29590263 | 4LX0_C | Site 1 | -9.0 |
| ZINC18141294 | 1OIV_A | Site 1 | -10.9 |
|  | 4OJK_A | Site 2 | -9.1 |
| ZINC01690699 | 4LX0_C | Site 1 | -10.5 |
|  | 5C46_F | Site 1 | -9.3 |
|  | 4UJ5_B | Site 2 | -9.0 |
|  | 1YZK_A | Site 2 | -9.3 |
|  | 5JCZ_D | Site 1 | -9.0 |
| ZINC04773602 | 1YZK_A | Site 2 | -7.1 |
| ZINC29590257 | 5C46_F | Site 1 | -9.8 |
|  | 4LX0_C | Site 1 | -10.1 |
|  | 4OJK_A | Site 2 | -8.7 |
| ZINC13099051 | 4LX0_C | Site 1 | -8.4 |
|  | 1OIV_A | Site 1 | -7.8 |
| ZINC01639634 | 4LX0_C | Site 1 | -8.4 |
| ZINC18057104 | 1OIV_A | Site 1 | -10.2 |
|  | 4LX0_C | Site 1 | -8.6 |
|  | 4UJ5_B | Site 2 | -7.8 |
| ZINC04783229 | 4LX0_C | Site 1 | -8.3 |
|  | 4OJK_A | Site 2 | -7.8 |
|  | 4UJ5_B | Site 1 | -8.5 |
| ZINC01694053 | 4LX0_C | Site 1 | -9.3 |
|  | 4C4P_A | Site 2 | -7.9 |
| ZINC01572309 | 1YZK_A | Site 2 | -8.7 |
|  | 4C4P_A | Site 2 | -8.2 |
|  | 4OJK_A | Site 2 | -8.8 |
|  | 1OIV_A | Site 1 | -8.4 |
| ZINC01707130 | 4LX0_C | Site 1 | -7.0 |
| ZINC01568793 | 4LX0_C | Site 1 | -8.0 |
|  | 5JCZ_D | Site 1 | -7.0 |
|  | 1OIV_A | Site 1 | -8.5 |
|  | 4C4P_A | Site 2 | -7.2 |
|  | 4UJ5_B | Site 2 | -6.5 |
| ZINC13152284 | 4LX0_C | Site 1 | -9.2 |
|  | 1OIV_A | Site 1 | -9.2 |
|  | 4UJ5_B | Site 2 | -7.8 |
