# Supplementary material for: Allosteric binding sites in Rab11 for potential drug candidates
Source: PLoS One. 2018 Jun 6;13(6):e0198632. doi: 10.1371/journal.pone.0198632 (PMC5991966; doi:10.1371/journal.pone.0198632)
Supplement: S6 Table — The target sites of ligands in 1OIV_A and their free energy of binding computed by Vinardo are listed. GDP stands for Guanosine-5'-Diphosphate. (DOCX) [file pone.0198632.s059.docx]

| **Ligand** | **Site** | **Free energy computed by (Kcal/mol)** |
| --- | --- | --- |
| ZINC00084617 | Site 1 | -9.4 |
| ZINC01578333 | Site 1 | -9.3 |
| ZINC13208966 | Site 1 | -8.1 |
| ZINC04720972 | Site 1 | -8.8 |
| ZINC11677172 | Site 1 | -7.7 |
| ZINC00393674 | Site 1 | -7.9 |
| ZINC01701460 | Site 1 | -8.7 |
