# Supplementary material for: Allosteric binding sites in Rab11 for potential drug candidates
Source: PLoS One. 2018 Jun 6;13(6):e0198632. doi: 10.1371/journal.pone.0198632 (PMC5991966; doi:10.1371/journal.pone.0198632)
Supplement: S7 Table — The target sites of ligands, and their free energy of binding computed by Vinardo are listed. GNP stands for Phosphoaminophosphonic acid-guanylate ester. (DOCX) [file pone.0198632.s060.docx]

| **Ligands** | **Targets** | **Site** | **Free energy (Kcal/mol)** |
| --- | --- | --- | --- |
| ZINC04773602 | 1YZK_A | Site 2 | -7.1 |
| ZINC15952559 | 4C4P_A | Site 2 | -8.7 |
| ZINC11677178 | 4C4P_A | Site 2 | -7.2 |
| ZINC12671898 | 4C4P_A | Site 2 | -6.5 |
| ZINC17353914 | 4UJ5_B | Site 1 | -8.0 |
| ZINC01573829 | 1YZK_A | Site 2 | -9.0 |
|  | 4C4P_A | Site 2 | -8.2 |
| ZINC01577889 | 1YZK_A | Site 2 | -9.2 |
| ZINC29590275 | 4UJ5_B | Site 1 | -8.5 |
| ZINC01726776 | 1YZK_A | Site 2 | -9.0 |
