# Supplementary material for: Allosteric binding sites in Rab11 for potential drug candidates
Source: PLoS One. 2018 Jun 6;13(6):e0198632. doi: 10.1371/journal.pone.0198632 (PMC5991966; doi:10.1371/journal.pone.0198632)
Supplement: S8 Table — The target sites of ligands and their free energy of binding computed by Vinardo are listed. GDP stands for Guanosine-5'-Diphosphate. (DOCX) [file pone.0198632.s061.docx]

| **Ligands** | **Site** | **Free energy (Kcal/mol)** |
| --- | --- | --- |
| ZINC17465979 | Site 1 | -7.5 |
| ZINC05462670 | Site 1 | -7.5 |
| ZINC05462674 | Site 1 | -7.6 |
| ZINC12672242 | Site 1 | -7.9 |
| ZINC17465983 | Site 1 | -7.5 |
| ZINC01668429 | Site 2 | -7.2 |
