# Supplementary material for: Allosteric binding sites in Rab11 for potential drug candidates
Source: PLoS One. 2018 Jun 6;13(6):e0198632. doi: 10.1371/journal.pone.0198632 (PMC5991966; doi:10.1371/journal.pone.0198632)
Supplement: S9 Table — Sequence identities of Rab1a, Rab1b, Rab11a and Rab11b as computed by Clustal Omega are shown above. (DOCX) [file pone.0198632.s062.docx]

|  | **Rab1a** | **Rab1b** | **Rab11a** | **Rab11b** |
| --- | --- | --- | --- | --- |
| **Rab1a** | 100.00 | 93.53 | 47.78 | 47.32 |
| **Rab1b** | 93.53 | 100.00 | 49.75 | 48.26 |
| **Rab11a** | 47.78 | 49.75 | 100.00 | 91.20 |
| **Rab11b** | 47.32 | 48.26 | 91.20 | 100.00 |
