# Supplementary material for: Allosteric binding sites in Rab11 for potential drug candidates
Source: PLoS One. 2018 Jun 6;13(6):e0198632. doi: 10.1371/journal.pone.0198632 (PMC5991966; doi:10.1371/journal.pone.0198632)
Supplement: S10 Table — The table above lists the shortest paths from site 1 and site 2 to the nucleotide binding site, that are observed to have minimal length during network analysis. The second column of the table shows the PDB entries for which such shortest paths are observed and the third column lists the shortest paths. (DOCX) [file pone.0198632.s063.docx]

| **Binding site** | **PDB entries** | **Shortest paths** |
| --- | --- | --- |
| Site 1 | 1OIV_A, 1YZK_A, 4C4P_A, 4LX0_C, 4OJK_A, 4UJ5_B, 5C46_F, 5JCZ_D | N101-Y98-D19-G21  N101Y-98-D91-V22 |
| Site 2 | 1OIV_A | V118-A87-L16-K24  I119-L88-V90-V22  I119-A87-L16-K24 |
| Site 2 | 1YZK_A, 4C4P_A, 4UJ5_B, 5C46_F, 5JCZ_D | E103-W105-G18-V22  E103-W105-G18-G69  E106-W105-G18-V22  L106-W105-G18-G69  L109-W105-G18-V22  L109-W105-G18-G69  V118-A87-L16-K24  I119-L89-V90-V22  I119-A87-L16-K24  I119-L89-G18-G69 |
| Site 2 | 4OJK_A | E103-V102-G18-V22  L106-W105-G18-V22  L109-W105-G18-V22  V118-A87-L16-K24  I119-L89-V90-V22  I119-A87-L16-K24 |
| Site 2 | 1OIV_A, 1YZK_A, 4C4P_A, 4LX0_C, 4OJK_A, 4UJ5_B, 5C46_F, 5JCZ_D | L148-L121-V90-V22  L148-L121-V90-K24  S149-L121-V90-V22  S149-L121-V90-K24 |
