# Supplementary material for: Allosteric binding sites in Rab11 for potential drug candidates
Source: PLoS One. 2018 Jun 6;13(6):e0198632. doi: 10.1371/journal.pone.0198632 (PMC5991966; doi:10.1371/journal.pone.0198632)
Supplement: S11 Table — (DOCX) [file pone.0198632.s064.docx]

| **Residues** | **1OIV_A** | **1YZK_A** | **4C4P_A** | **4LX0_C** | **4OJK_A** | **4UJ5_B** | **5C46_F** | **5JCZ_D** |
| --- | --- | --- | --- | --- | --- | --- | --- | --- |
| A87 | 0.067744 | 0.046389 | 0.062286 | 0.053951 | 0.060761 | 0.050912 | 0.06571 | 0.063511 |
| L89 | 0.037948 | 0.056417 | 0.049945 | 0.041699 | 0.05041 | 0.054352 | 0.063119 | 0.056236 |
| W105 | 0.061756 | 0.056489 | 0.057174 | 0.066342 | 0.043554 | 0.059649 | 0.056209 | 0.04658 |
| I119 | 0.056129 | 0.04372 | 0.043373 | 0.048381 | 0.067617 | 0.051324 | 0.063119 | 0.054944 |
