# Supplementary material for: Allosteric binding sites in Rab11 for potential drug candidates
Source: PLoS One. 2018 Jun 6;13(6):e0198632. doi: 10.1371/journal.pone.0198632 (PMC5991966; doi:10.1371/journal.pone.0198632)
Supplement: S1 File — (PDF) [file pone.0198632.s065.pdf]

Subcellular localizations and molecular functions of Rab11 proteins

Rab11a localizes to the endocytic recycling compartment (ERC)/recycling endosome [1][2][3] and trans-Golgi network (TGN) compartment [4] to control the vesicle trafficking through and from these compartments [5]. Rab11a controls tissue homeostasis during both embryonic development and postnatal periods [6][5]. Recently, Yan *et al.* showed that Rab11a regulates vascular endothelial-cadherin protein recycling to endothelial cell plasma membrane [7]. Intestinal Rab11a is also required for the apical protein localization [6].

Rab11b also localizes to ERC [5] and regulates the recycling of transferrin receptor to the plasma membrane [8]. Rab11b regulates exocytosis in neurons and neuroendocrine cells [9]. However, little is known about the functional differences between Rab11a and Rab11b. They localize to different vesicle compartments in gastric parietal cells [10]. Silvis *et al*. have shown that Rab11b, but not Rab11a, specifically regulates the recycling of the intracellular cystic fibrosis transmembrane conductance regulator (CFTR) in polarized epithelial cells in the intestines [11][5]. Later, Haugsten *et al.* have shown that Rab11a and Rab11b may play slightly different roles in fibroblast growth factor receptor 4 (FGFR4) recycling [12]. While knockdown of Rab11a, Rab11b or Rab11a/b simultaneously reduced FGFR4 transport out of the ERC, knockdown of Rab11b alone but not Rab11a, accumulated FGFR4 in a perinuclear compartment.

Ras and Rab1 proteins

Previously, we have identified novel binding sites in Ras and Rab1 proteins [13][14]. Ras is a family of proteins in the Ras superfamily of proteins that regulates signaling pathways that control gene expression of cell growth, differentiation and survival. Three members of this family: K-Ras, H-Ras and N-Ras, are frequently mutated in cancer and hyper-proliferative

developmental disorders [15]. K-Ras localizes to cytosol and plasma membrane. H-Ras and N-Ras localizes to golgi apparatus and plasma membrane. These isoforms share more than 85% identity. Through computational and experimental methods, we have previously identified three allosteric pockets and inhibitors for Ras [14]. Rab1 is a member of the Rab GTPase family that regulates membrane trafficking pathways that are related to transport between endoplasmic reticulum and golgi apparatus, and autophagy [16]. Rab1 localizes to ER, GA and early endosome [16][17]. It has two isoforms, Rab1a and Rab1b, that share 92% of sequence identity [18]. Rab1 is associated with various human cancers including prostate cancer [19], triple-negative breast cancer (TNBC) [20], colorectal cancer [21] and tongue cancer [22]. Aberrant expression of Rab1 is also associated with diseases such as cardiac hypertrophy [23] and Parkinson's disease [24].

Principal Component Analysis (PCA), Independent Component Analysis (ICA) and Locally Linear Embedding (LLE)

PCA and ICA are linear dimensionality reduction techniques. PCA projects data from high dimensional space to low dimensional space such that the variance of data is maximized, assuming that the direction with the biggest variance is the most important [25][26]. ICA performs dimensionality reduction by deriving independent components from the high dimensional data in such a way that maximizes non-Gaussianity [26]. While PCA minimizes co-variance of data, ICA minimizes mutual information of data [25]. LLE is a manifold learning algorithm [27] which is used for non-linear dimensionality reduction. LLE can identify the underlying structure of the manifold better than PCA and ICA [28].

PCA and Dynamical Cross Correlation Analysis (DCCM) analysis on the ensemble of 28 Rab11 structures

We first performed PCA on the ensemble of 28 structures. More than 80% of the variance is captured in the first three principal components (PCs) (S1.1 Fig).

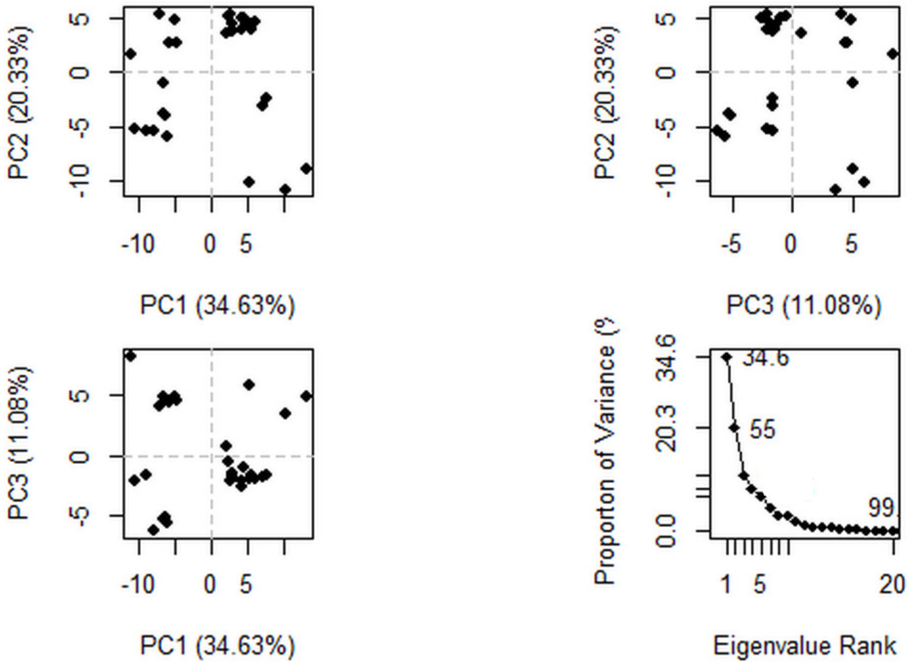

S1.1 Fig. Results of PCA on the ensemble of 28 Rab11 structures.

We projected the structures in the Rab11 ensemble onto the first two PCs and, the first and third PCs. More than 60% of the variance is captured in these PCs (S1.2 Fig). We observed that 2F9L_A is separated from other structures.

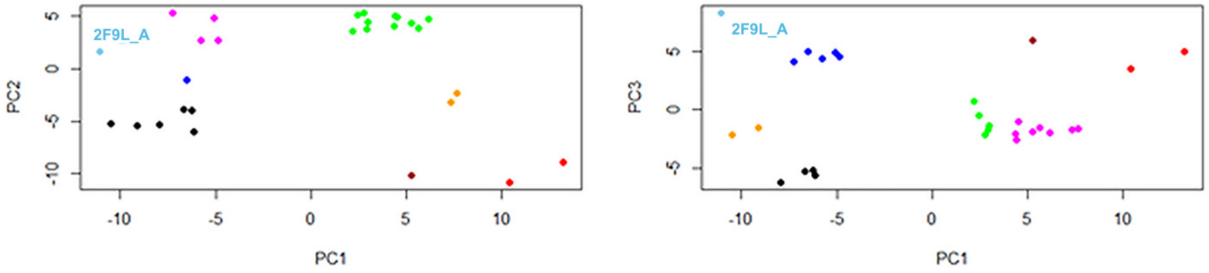

S1.2 Fig. Projection of structures from the ensemble of 28 structures onto the PC space. PDB entry 2F9L_A (labeled) that lacks residues E39-K41 is separated from all other structures.

On examining the contribution of each residue to the first three principal components, we observed that the largest contributions are made by residues in switch 1 (E39-V46), switch 2 (A68-A79) and interswitch (E47-T67) regions of Rab11 (S1.3 Fig).

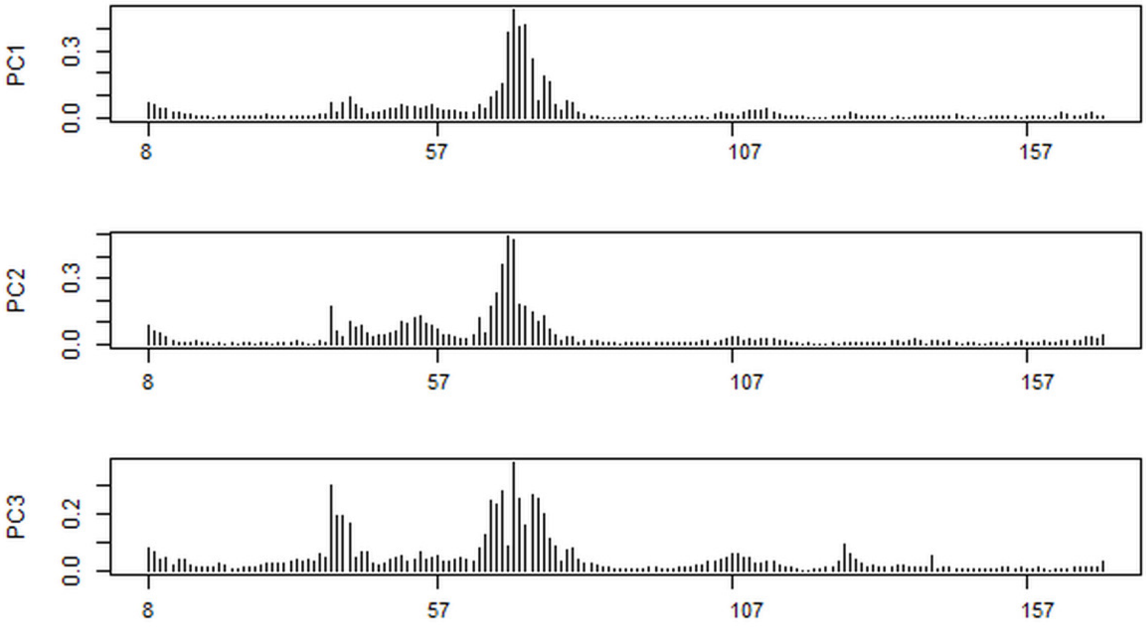

S1.3 Fig. Residual contributions of Rab11 structures in PC1, PC2 and PC3.

Furthermore, Dynamical Cross-Correlation Matrix (DCCM) analysis [29] of the superposed Cartesian coordinates of C_α atoms of Rab11 structures shown that there are correlated motions within these regions (S1.4 Fig). Since switch 1 region is found to be important in Rab11, we excluded 2F9L_A which lacks coordinates of this region from further analyses.

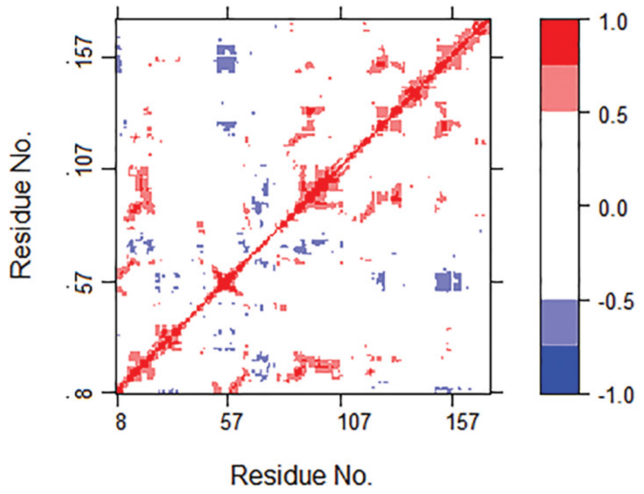

S1.4 Fig. Cross-correlated motions in Rab11. Red and blue colors represent positive and negative correlations, respectively. Motion occurring along the same direction is represented by positive correlation (red), whereas motion occurring along opposite directions is represented by negative (anti-) correlation (blue). Positive correlations with values greater than 0.5 and negative correlations with values less than -0.5 are shown.

References

1. Rab11 regulates recycling through the pericentriolar recycling endosome. J Cell Biol. 1996;135: 913–924.

2. Maxfield FR, McGraw TE. Endocytic recycling. Nat Rev Mol Cell Biol. 2004;5: 121–132. doi:10.1038/nrm1315

3. Grant BD, Donaldson JG. Pathways and mechanisms of endocytic recycling. Nat Rev Mol Cell Biol. 2009;10: 597–608. doi:10.1038/nrm2755

4. Chen W, Feng Y, Chen D, Wandinger-Ness A. Rab11 is required for trans-golgi network-to-plasma membrane transport and a preferential target for GDP dissociation inhibitor. Mol Biol Cell. 1998;9: 3241–3257.

5. Campa CC, Hirsch E. Rab11 and phosphoinositides: A synergy of signal transducers in the control of vesicular trafficking. Adv Biol Regul. 2017;63: 132–139. doi:10.1016/j.jbior.2016.09.002

6. Sobajima T, Yoshimura S, Iwano T, Kunii M, Watanabe M, Atik N, et al. Rab11a is required for apical protein localisation in the intestine. Biol Open. 2014;4: 86–94. doi:10.1242/bio.20148532

7. Yan Z, Wang Z-G, Segev N, Hu S, Minshall RD, Dull RO, et al. Rab11a Mediates Vascular Endothelial-Cadherin Recycling and Controls Endothelial Barrier FunctionSignificance. Arterioscler Thromb Vasc Biol. 2016;36: 339–349. doi:10.1161/ATVBAHA.115.306549

8. Schlierf B, Fey GH, Hauber J, Hocke GM, Rosorius O. Rab11b is essential for recycling of transferrin to the plasma membrane. Exp Cell Res. 2000;259: 257–265. doi:10.1006/excr.2000.4947

9. Khvotchev MV, Ren M, Takamori S, Jahn R, Südhof TC. Divergent Functions of Neuronal Rab11b in Ca2+-Regulated versus Constitutive Exocytosis. J Neurosci. 2003;23: 10531–10539.

10. Lapierre LA, Dorn MC, Zimmerman CF, Navarre J, Burnette JO, Goldenring JR. Rab11b resides in a vesicular compartment distinct from Rab11a in parietal cells and other epithelial cells. Exp Cell Res. 2003;290: 322–331.

11. Silvis MR, Bertrand CA, Ameen N, Golin-Bisello F, Butterworth MB, Frizzell RA, et al. Rab11b regulates the apical recycling of the cystic fibrosis transmembrane conductance regulator in polarized intestinal epithelial cells. Mol Biol Cell. 2009;20: 2337–2350. doi:10.1091/mbc.E08-01-0084

12. Haugsten EM, Brech A, Liestøl K, Norman JC, Wesche J. Photoactivation Approaches Reveal a Role for Rab11 in FGFR4 Recycling and Signalling: FGFR4 Recycling is Dependent on Rab11. Traffic. 2014;15: 665–683. doi:10.1111/tra.12168

13. Lukman S, Nguyen MN, Sim K, Teo JCM. Discovery of Rab1 binding sites using an ensemble of clustering methods. Proteins. 2017;85: 859–871. doi:10.1002/prot.25254

14. Grant BJ, Lukman S, Hocker HJ, Sayyah J, Brown JH, McCammon JA, et al. Novel Allosteric Sites on Ras for Lead Generation. PLOS ONE. 2011;6: e25711. doi:10.1371/journal.pone.0025711

15. Quinlan MP, Settleman J. Isoform-specific ras functions in development and cancer. Future Oncol. 2009;5: 105–116. doi:10.2217/14796694.5.1.105

16. Dong N, Zhu Y, Lu Q, Hu L, Zheng Y, Shao F. Structurally distinct bacterial TBC-like GAPs link Arf GTPase to Rab1 inactivation to counteract host defenses. Cell. 2012;150: 1029–1041. doi:10.1016/j.cell.2012.06.050

17. Mukhopadhyay A, Nieves E, Che F-Y, Wang J, Jin L, Murray JW, et al. Proteomic analysis of endocytic vesicles: Rab1a regulates motility of early endocytic vesicles. J Cell Sci. 2011;124: 765–775. doi:10.1242/jcs.079020

18. Touchot N, Chardin P, Tavitian A. Four additional members of the ras gene superfamily isolated by an oligonucleotide strategy: molecular cloning of YPT-related cDNAs from a rat brain library. Proc Natl Acad Sci U S A. 1987;84: 8210–8214.

19. Abd Elmageed ZY, Yang Y, Thomas R, Ranjan M, Mondal D, Moroz K, et al. Neoplastic Reprogramming of Patient-Derived Adipose Stem Cells by Prostate Cancer Cell-Associated Exosomes. Stem Cells Dayt Ohio. 2014;32: 983–997. doi:10.1002/stem.1619

20. Jiang H-L, Sun H-F, Gao S-P, Li L-D, Hu X, Wu J, et al. Loss of RAB1B promotes triple-negative breast cancer metastasis by activating TGF-β/SMAD signaling. Oncotarget. 2015;6: 16352–16365. doi:10.18632/oncotarget.3877

21. Thomas JD, Zhang Y, Wei Y, Cho J-H, Morris LE, Wang H-Y, et al. Rab1A Is an mTORC1 Activator and a Colorectal Oncogene. Cancer Cell. 2014;26: 754–769. doi:10.1016/j.ccell.2014.09.008

22. Shimada K, Uzawa K, Kato M, Endo Y, Shiiba M, Bukawa H, et al. Aberrant expression of RAB1A in human tongue cancer. Br J Cancer. 2005;92: 1915–1921. doi:10.1038/sj.bjc.6602594

23. Wu G, Yussman MG, Barrett TJ, Hahn HS, Osinska H, Hilliard GM, et al. Increased Myocardial Rab GTPase Expression: A Consequence and Cause of Cardiomyopathy. Circ Res. 2001;89: 1130–1137. doi:10.1161/hh2401.100427

24. Cooper AA, Gitler AD, Cashikar A, Haynes CM, Hill KJ, Bhullar B, et al. α-Synuclein Blocks ER-Golgi Traffic and Rab1 Rescues Neuron Loss in Parkinson's Models. Science. 2006;313: 324–328. doi:10.1126/science.1129462

25. Delac K, Grgic M, Grgic S. Independent comparative study of PCA, ICA, and LDA on the FERET data set. Int J Imaging Syst Technol. 2005;15: 252–260. doi:10.1002/ima.20059

26. Šafránková J, editor. 19th Annual Conference of Doctoral Students, WDS'10 "Week of Doctoral Students 2010", Charles University, Faculty of Mathematics and Physics, Prague, Czech Republic, June 1, 2010 to June 4, 2010: proceedings of contributed papers. Pt. 1: Mathematics and computer sciences. Praha: Matfyzpress; 2010.

27. Roweis ST, Saul LK. Nonlinear dimensionality reduction by locally linear embedding. Science. 2000;290: 2323–2326. doi:10.1126/science.290.5500.2323

28. Karbauskaitė R, Kurasova O, Dzemyda G. SELECTION OF THE NUMBER OF NEIGHBOURS OF EACH DATA POINT FOR THE LOCALLY LINEAR EMBEDDING ALGORITHM. Inf Technol Control. 2015;36. doi:10.5755/j01.itc.36.4.11890

29. Grant BJ, Rodrigues APC, ElSawy KM, McCammon JA, Caves LSD. Bio3d: an R package for the comparative analysis of protein structures. Bioinformatics. 2006;22: 2695–2696. doi:10.1093/bioinformatics/btl461
