# Supplementary material for: Allosteric binding sites in Rab11 for potential drug candidates
Source: PLoS One. 2018 Jun 6;13(6):e0198632. doi: 10.1371/journal.pone.0198632 (PMC5991966; doi:10.1371/journal.pone.0198632)
Supplement: S2 File — (PDF) [file pone.0198632.s066.pdf]

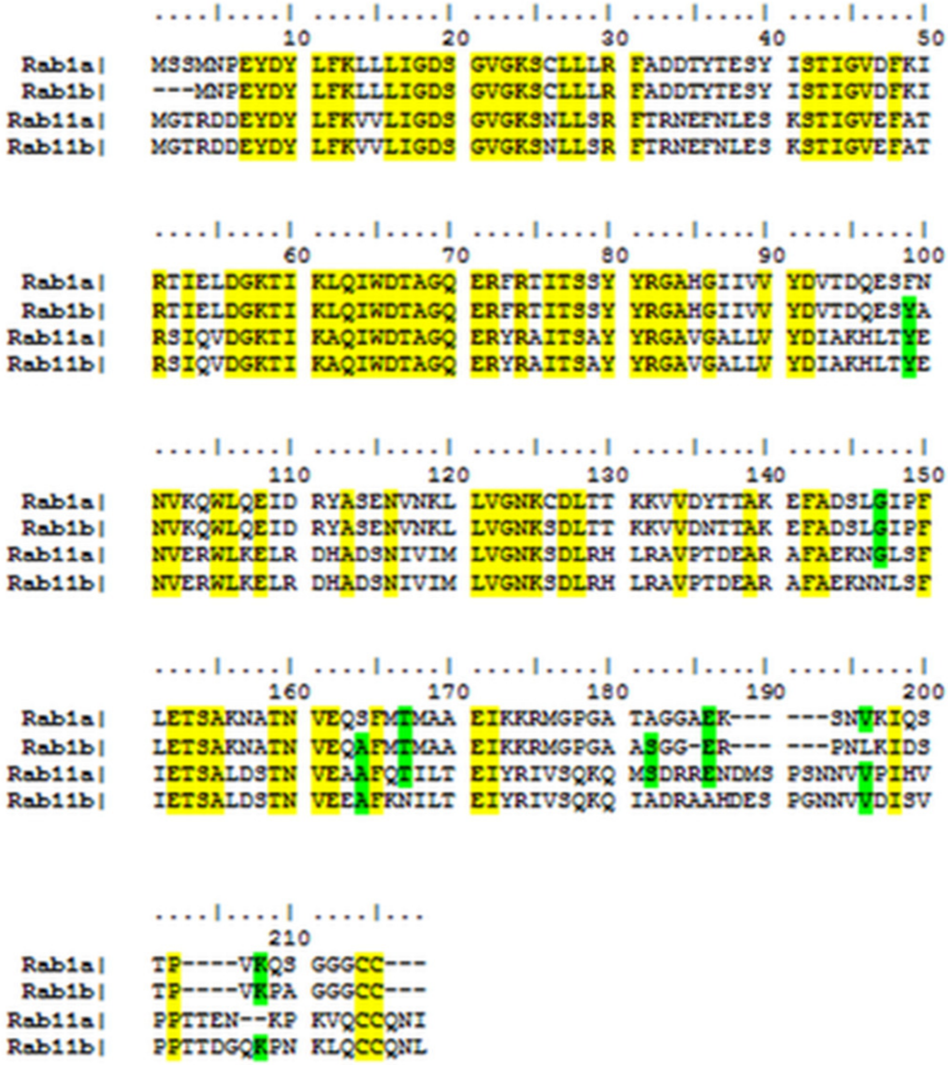

S2.1 Fig. Multiple sequence alignment of Rab1 and Rab11 sequences. Residues that are conserved in all sequences are highlighted in yellow and residues that are conserved in two or more sequences are highlighted in green.

```
                         G1                        G2
               10         20         30         40         50
Rab11a|   MGTRDDEYDY LFKVVLIGDS GVGKSNLLSR FTRNEFNLES KSTIGVEFAT
Rab11b|   MGTRDDEYDY LFKVVLIGDS GVGKSNLLSR FTRNEFNLES KSTIGVEFAT

                      G3
               60         70         80         90        100
Rab11a|   RSIQVDGKTI KAQIWDTAGQ ERYRAITSAY YRGAVGALLV YDIAKHLTYE
Rab11b|   RSIQVDGKTI KAQIWDTAGQ ERYRAITSAY YRGAVGALLV YDIAKHLTYE

                            G4
              110        120        130        140        150
Rab11a|   NVERWLKELR DHADSN-IVI MLVGNKSDLR HLRAVPTDEA RAFAEKNGLS
Rab11b|   NVERWLKELR DHADSN-IVI MLVGNKSDLR HLRAVPTDEA RAFAEKNNLS

             G5
              160        170        180        190        200
Rab11a|   FIETSALDST NVEAAFQTIL TEIYRIVSQK QMSDRRENDM SPSNNVVPIH
Rab11b|   FIETSALDST NVEEAFKNIL TEIYRIVSQK QIADRAAHDE SPGNNVVDIS

              210
Rab11a|   VPPTTENKP- -KVQCCQNI
Rab11b|   VPPTTDGQKP NKLQCCQNL
```

S2.2 Fig. Pairwise sequence alignment of Rab11a and Rab11b. The G loops are colored in red. The amino acid variations in Rab11b sequence compared to those of Rab11a are highlighted in green.
