# Supplementary material for: Allosteric binding sites in Rab11 for potential drug candidates
Source: PLoS One. 2018 Jun 6;13(6):e0198632. doi: 10.1371/journal.pone.0198632 (PMC5991966; doi:10.1371/journal.pone.0198632)
Supplement: S3 File — (PDF) [file pone.0198632.s067.pdf]

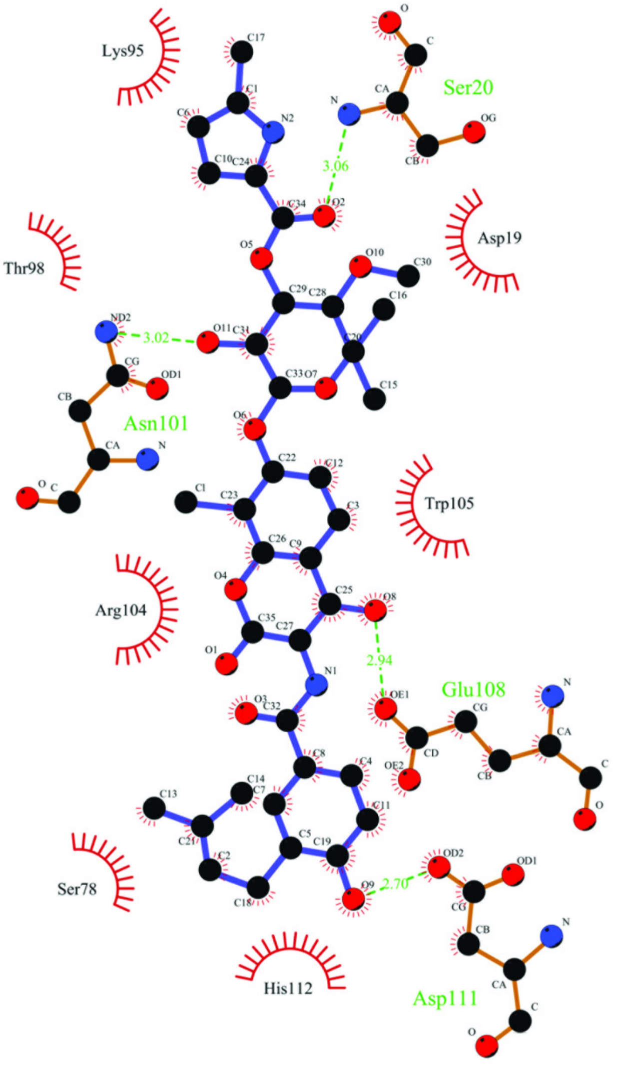

4LX0_C

S3.1 Fig. Ligand ZINC29590259 docked at site 1 of PDB entry 4LX0_C. The ligand and Rab11 side chains are shown in ball-and-stick representation. The ligand bonds are colored in purple. Hydrogen bonds are shown as green dotted lines. The Rab11 residues making nonbonded contacts with the ligand are shown as spoked arcs. The figure is generated using LigPlot+.

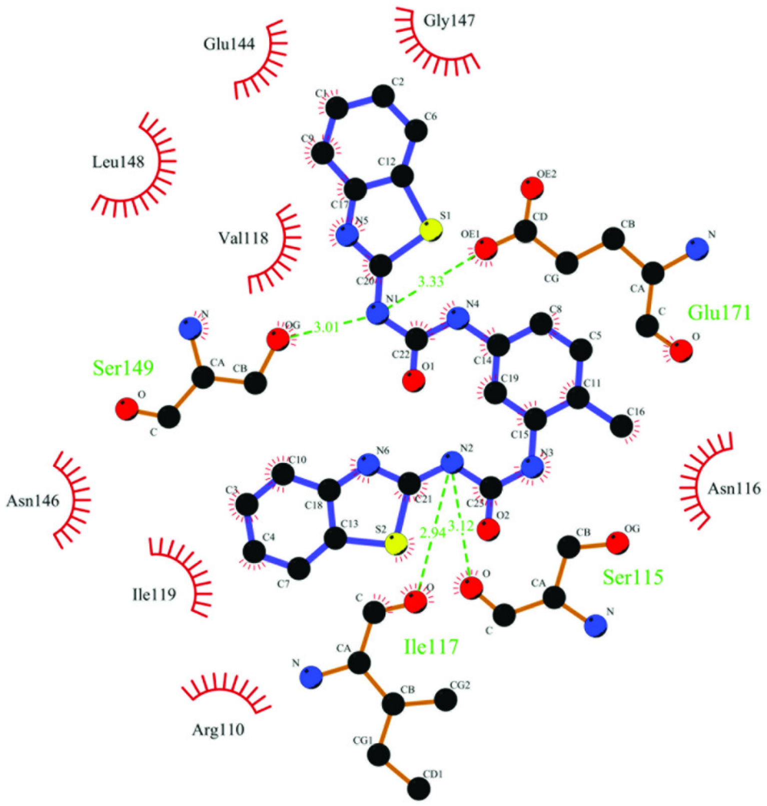

4C4P_A

S3.2. Ligand ZINC01572309 docked at site 2 of PDB entry 4C4P_A. The ligand and Rab11 side chains are shown in ball-and-stick representation. The ligand bonds are colored in purple. Hydrogen bonds are shown as green dotted lines. The Rab11 residues making nonbonded contacts with the ligand are shown as spoked arcs. The figure is generated using LigPlot+.

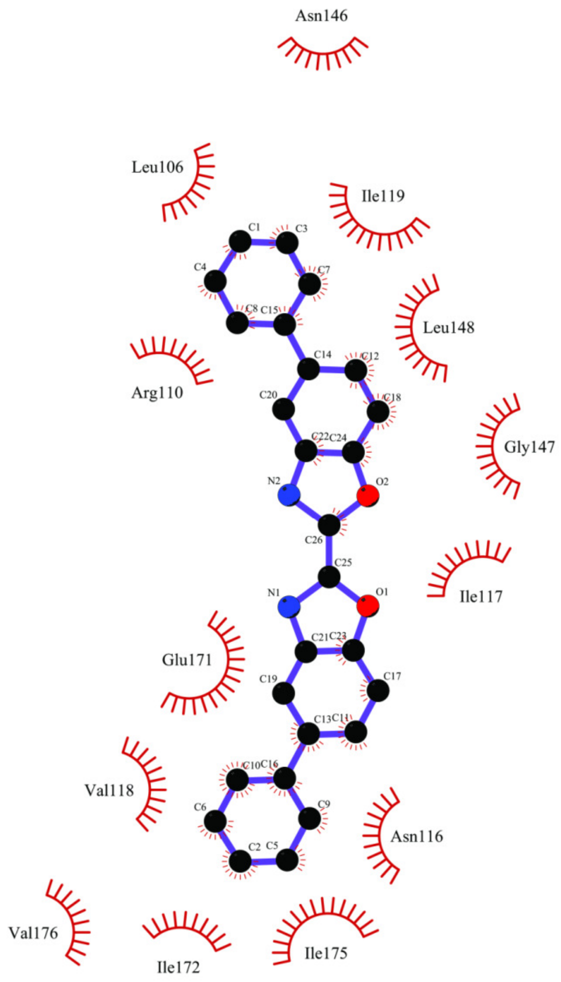

S3.3. Ligand ZINC13152284 docked at site 2 of PDB entry 4UJ5_B. The ligand and Rab11 side chains are shown in ball-and-stick representation. The ligand bonds are colored in purple. Hydrogen bonds are shown as green dotted lines. The Rab11 residues making nonbonded contacts with the ligand are shown as spoked arcs. The figure is generated using LigPlot+.

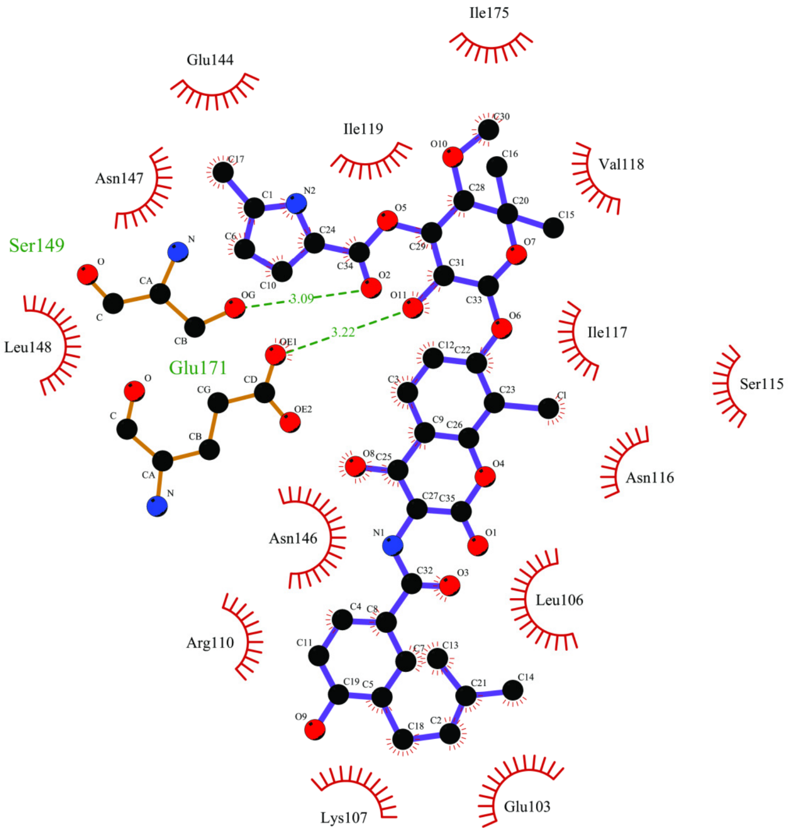

4OJK_A

S3.4 Fig. Ligand ZINC29590263 docked at site 2 of PDB entry 4OJK_A. The ligand and Rab11 side chains are shown in ball-and-stick representation. The ligand bonds are colored in purple. Hydrogen bonds are shown as green dotted lines. The Rab11 residues making nonbonded contacts with the ligand are shown as spoked arcs. The figure is generated using LigPlot+ .
